# Replicated analysis of the genetic architecture of quantitative traits in two wild great tit populations

**DOI:** 10.1111/mec.13452

**Published:** 2015-12-10

**Authors:** Anna W. Santure, Jocelyn Poissant, Isabelle De Cauwer, Kees van Oers, Matthew R. Robinson, John L. Quinn, Martien A. M. Groenen, Marcel E. Visser, Ben C. Sheldon, Jon Slate

**Affiliations:** ^1^ School of Biological SciencesUniversity of AucklandAuckland1010New Zealand; ^2^ Department of Animal and Plant SciencesUniversity of SheffieldSheffieldS10 2TNUK; ^3^ Centre for Ecology and ConservationUniversity of ExeterPenryn CampusPenrynTR10 9FEUK; ^4^ Unité Evolution, Ecologie et PaléontologieUMR 8198Université de Lille – Sciences et Technologies59655 CedexVilleneuve d'AscqFrance; ^5^ Department of Animal EcologyNetherlands Institute of Ecology (NIOO‐KNAW)6708 PBWageningenThe Netherlands; ^6^ Queensland Brain InstituteUniversity of QueenslandBrisbaneQld4072Australia; ^7^ School of Biological, Earth and Environmental SciencesUniversity College CorkDistillery Fields, North Mall, CorkIreland; ^8^ Department of ZoologyEdward Grey InstituteUniversity of OxfordOxfordOX1 3PSUK; ^9^ Animal Breeding and Genomics CentreWageningen UniversityDe Elst 1WageningenThe Netherlands

**Keywords:** chromosome partitioning, genome‐wide association, genomics, GWAS, QTL mapping, quantitative genetics

## Abstract

Currently, there is much debate on the genetic architecture of quantitative traits in wild populations. Is trait variation influenced by many genes of small effect or by a few genes of major effect? Where is additive genetic variation located in the genome? Do the same loci cause similar phenotypic variation in different populations? Great tits (*Parus major*) have been studied extensively in long‐term studies across Europe and consequently are considered an ecological ‘model organism’. Recently, genomic resources have been developed for the great tit, including a custom SNP chip and genetic linkage map. In this study, we used a suite of approaches to investigate the genetic architecture of eight quantitative traits in two long‐term study populations of great tits—one in the Netherlands and the other in the United Kingdom. Overall, we found little evidence for the presence of genes of large effects in either population. Instead, traits appeared to be influenced by many genes of small effect, with conservative estimates of the number of contributing loci ranging from 31 to 310. Despite concordance between population‐specific heritabilities, we found no evidence for the presence of loci having similar effects in both populations. While population‐specific genetic architectures are possible, an undetected shared architecture cannot be rejected because of limited power to map loci of small and moderate effects. This study is one of few examples of genetic architecture analysis in replicated wild populations and highlights some of the challenges and limitations researchers will face when attempting similar molecular quantitative genetic studies in free‐living populations.

## Introduction

Studying genetic architecture in natural populations is essential for understanding the evolutionary history, and the adaptive potential, of quantitative traits in the wild (Clutton‐Brock & Sheldon 2010; Teplitsky *et al*. 2014). Considerable progress has been made in understanding the genetics of quantitative trait variation in genetic model species (Flint & Mackay 2009), in humans (Yang *et al*. 2011) and in species of agricultural and horticultural importance (Hill 2009). However, the lack of genetic resources and the difficulties of controlling environmental influences have made the molecular dissection of quantitative traits considerably more challenging in wild vertebrate populations (Slate *et al*. 2010; Jensen *et al*. 2014; Schielzeth & Husby 2014).

Much of the research on the genetic architecture of quantitative traits in wild populations has focused on long‐term studies of populations with known pedigree relationships, but with small population size and often limited migration (Slate *et al*. 2010; Schielzeth & Husby 2014). Quantitative trait locus (QTL) mapping in these populations has revealed a number of loci potentially influencing quantitative traits and provided some support for genes of major effect (see, for example, Slate *et al*. 2002; Tarka *et al*. 2010; Poissant *et al*. 2012; Johnston *et al*. 2013). However, estimated QTL effect sizes from studies in natural populations to date are almost certain to be overestimated. This is because overestimated effect sizes are more likely than underestimated effect sizes to reach statistical significance (Beavis 1994; Xu 2003; Slate 2013). More recently, much larger sets of markers have enabled genome‐wide association studies in wild populations (Santure *et al*. 2013; Bérénos *et al*. 2015; Husby *et al*. 2015). These more recent studies suggest that while some regions of the genome may explain a significant proportion of the overall heritability, there is likely a very large number of small‐effect loci that together contribute the majority of heritability for quantitative traits. Overall, whether traits are influenced by many genes of (mostly) small effect (i.e. have a polygenic basis) or few genes of large effect (i.e. have an oligogenic basis) will influence how populations in the wild will be able to adapt to changing environmental conditions. If many loci contribute to a quantitative trait, linkage disequilibrium (LD) between loci will influence the response to selection (Hill & Robertson 1966). For example, deleterious alleles may be fixed as a consequence of selection on a linked beneficial allele, reducing the total response to selection had beneficial alleles been fixed at both loci (Hill & Robertson 1966; Hospital & Chevalet 1996). Similarly, these small‐effect loci will likely contribute to other traits through pleiotropic effects (either directly or via LD between loci contributing to the traits), which may be subject to conflicting selection pressures (Lande 1982). In contrast, the simpler architecture of oligogenic traits, with QTL less likely to be linked to each other or to QTL for other traits, is likely to result in fewer constraints on the response to selection.

Great tits (*Parus major*) are considered an ecological model organism and have been used to investigate the evolution and ecology of a wide range of phenotypes, for example, life history, morphological, behavioural and physiological traits, as well as phenotypic responses to climate change across their range (see, for example, Lack 1964; Drent *et al*. 2003; Visser *et al*. 2003; Garant *et al*. 2004; Gienapp *et al*. 2006; Quinn *et al*. 2009; Bouwhuis *et al*. 2010; Chapman & Sheldon 2011). Their extensive Eurasian distribution, abundance and amenity to using nest boxes lend great tit populations to detailed field study (Gosler 1993). Two of the longest running great tit studies have been ongoing since the 1950s and are located in the Hoge Veluwe National Park (Netherlands, NL; van Balen 1973) and Wytham Woods (United Kingdom, UK; see, for example, McCleery *et al*. 2004 and references therein). In the late 1980s, a subpopulation study was established adjoining Hoge Veluwe at Westerheide, near Arnhem, NL (Dingemanse *et al*. 2002). In both NL and UK populations, trait data and social pedigree relationships have been recorded for most individuals. Maternal, morphological and personality traits have been the focus of pedigree‐based quantitative genetic studies, and in both populations these traits have moderate heritabilities (see, for example, Garnett 1981; van Noordwijk *et al*. 1988; Dingemanse *et al*. 2002; McCleery *et al*. 2004; Quinn *et al*. 2009; Husby *et al*. 2010), offering the opportunity to dissect the genetic basis of multiple quantitative traits in two independent populations.

Genomic resources have recently been developed in the great tit, including a single nucleotide polymorphism (SNP) array with 9193 SNPs (van Bers *et al*. 2012) and an associated genetic linkage map based on these SNPs (van Oers *et al*. 2014). The availability of markers distributed throughout the great tit genome offers the opportunity to determine whether traits are polygenic or oligogenic (Jensen *et al*. 2014). Recent research on the genetic architecture of clutch size, egg mass and wing length in the UK population (Robinson *et al*. 2013; Santure *et al*. 2013) lends support to the hypothesis that the majority of quantitative traits are influenced by many genes of small effect distributed throughout the genome (Mackay *et al*. 2009; Yang *et al*. 2011). Whether this conclusion can be extended to other quantitative traits measured in this species and to different populations remains to be determined.

In this study, a panel of SNP markers was used to dissect the genetic architecture of three maternal traits (clutch size, egg mass and offspring weight at fledging), four morphological traits (adult weight, weight at fledging, tarsus length and wing length) and one behavioural trait (exploratory behaviour) in the NL and UK great tit populations. Four different marker‐based approaches were applied in both populations: chromosome partitioning (Visscher *et al*. 2007; Robinson *et al*. 2013), quantitative trait locus mapping (Lynch & Walsh 1998; Slate 2005), genome‐wide association (Amin *et al*. 2007; Aulchenko *et al*. 2007a) and estimating the number of loci contributing to variance (Guan & Stephens 2011). By using different and somewhat independent yet complementary approaches, all available marker and phenotype information can be exploited to explore the genetic basis of traits in the two populations, in particular, to determine whether there is evidence for genes of large effect and to test whether the trait architectures are concordant between the two populations.

## Methods

### Study populations

The long‐term study populations of great tits are located at the Hoge Veluwe National Park and the nearby Westerheide, the Netherlands (NL) (52°02′N, 5°51′E and 52°01′N, 5°50′E, respectively; given the proximity of Westerheide and Hoge Veluwe (~5 km), and known migration between the two regions, individuals were merged into a single NL population in all analyses), and Wytham Woods, United Kingdom (UK) (51°46′N, 1°20′W). Both populations have been intensively studied (see Appendix S1, Supporting information for representative variables measured in the two populations) (van Balen 1973; Dingemanse *et al*. 2002; McCleery *et al*. 2004), and blood samples have been taken from most birds in the populations since 2005.

### Genotypes and genetic maps

A total of 1490 NL and 2644 UK wild individuals were successfully genotyped on an Illumina iSelect BeadChip (‘SNP chip’) (van Bers *et al*. 2012). Following pedigree and identity checking (Appendix S2, Supporting information; Santure *et al*. 2013; van Oers *et al*. 2014), a total of 1407 NL and 2497 UK individuals of confirmed identity were included in further analyses. A subsample of related individuals from the UK population and a captive population derived from the NL population were used to construct two independent linkage maps (van Oers *et al*. 2014). The linkage maps cover 32 of the 39 great tit chromosomes: 1–15, 17–24, 26–28, 1A, 4A, 25A, 25B, LGE22 and Z; a number of very small microchromosomes, including chromosome 16, could not be mapped as no SNPs on these chromosomes were genotyped. The maps were almost identical between the two populations; therefore in the subsequent analyses, the UK map distances were used. The different downstream analyses required linkage maps with different marker densities. For QTL mapping, a ‘framework without LD’ linkage map of 1524 markers was used, containing the majority of markers from the framework linkage map (Santure *et al*. 2013; van Oers *et al*. 2014). Markers on the framework map were placed at map positions where the best order was 1000 times more likely than any other order. Note that a small number of marker pairs with high linkage disequilibrium between them appeared to contribute to convergence problems during QTL mapping. Therefore, one of the two markers was excluded from the framework map before analysis to give the ‘framework without LD’ map, which covers 1.893 cM, with an average intermarker distance of 1.209 cM. For the genome‐wide association study (GWAS) and chromosome partitioning analyses, a set of 5591 ‘chromosome‐assigned’ markers was used. The chromosome‐assigned marker set included 4878 markers placed in a ‘parsimonious’ linkage map (where markers are added and the order with the highest likelihood is chosen) plus an additional 713 markers that were linked to markers in the parsimonious map and could be assigned a putative mapping position (in cM) based on comparative genomics with their predicted zebra finch genome location (Santure *et al*. 2013).

### Phenotypes

A number of quantitative traits were measured on the genotyped individuals in the two populations (Table 1). Egg mass was only available for individuals from the UK population. Repeated measures were available for all traits except exploratory behaviour and fledgling weight (of individual) (Table 1). Important fixed and random effects were identified to account for sources of variation in addition to the additive genetic variance (Appendix S1, Supporting information).

**Table 1:** Number of individuals [records available] for GWAS, chromosome (chr) partitioning and QTL mapping for quantitative traits in the NL and UK populations

Trait		NL GWAS/chr partitioning	NL QTL mapping	UK GWAS/chr partitioning	UK QTL mapping
Maternal	Clutch size	943 [1589]	403 [745]	1026 [1794]	722 [1362]
	Egg mass	—	—	960 [1619]	678 [1224]
	Fledgling weight (of offspring)	744 [8569]	327 [4146]	441 [4221]	328 [3167]
Morphological	Adult weight	477 [1547]	408 [1365]	1872 [3904]	1360 [2937]
	Fledgling weight (of individual)	416 [416]	357 [357]	1222 [1222]	1183 [1183]
	Tarsus length	1378 [2586]	653 [1415]	872 [1921]	626 [1379]
	Wing length	1275 [1908]	590 [901]	1949 [4293]	1410 [3221]
Behaviour	Exploratory behaviour	912 [912]	462 [462]	1046 [1046]	743 [743]

### Genetic analyses

#### Pedigree‐ and marker‐based heritabilities

Trait heritabilities were determined by two approaches, first (i) using pedigree information from each population and second (ii) using the genomic marker relatedness between individuals. For each trait in each population, heritabilities were estimated using a linear mixed model (the ‘animal model’, Henderson 1984; Kruuk 2004), where the relatedness between individuals was estimated from the pedigree (A matrix; approach i) or where the relatedness between individuals was estimated from the marker data (G matrix; approach ii).

For (i), for consistency with the quantitative trait locus analysis (see below), the pedigree was restricted to genotyped related individuals. By including all first‐ to fourth‐degree family links, 666 NL individuals and 1733 UK individuals (Santure *et al*. 2013) were included in a ‘QTL pedigree’ for the NL and UK, respectively. Variance components were estimated in a restricted maximum‐likelihood (REML) framework using asreml v3 (Gilmour *et al*. 2009), fitting random and fixed effects as described in Appendix S1 (Supporting information). The significance of the heritabilities was tested by comparing the log likelihood of a model where the heritability was set to zero (*L*_0_) to the log likelihood of the full model (*L*_1_) with a likelihood ratio test (LRT):

LRT = −2(*L*_0_−*L*_1_)

Under the null hypothesis, the LRT follows a 50:50 distribution of a chi‐square with zero degrees of freedom and a chi‐square with one degree of freedom (Almasy & Blangero 1998).

For (ii), the genomic relatedness between every pair of individuals within each population was calculated using an approach that scales by the actual variance in relatedness (approach 3; Robinson *et al*. 2013). These genomic estimates of relatedness were then adjusted by the known pedigree relationships between individuals, weighting the marker‐derived relatedness values towards their expected pedigree‐based values. This approach reduces the sampling error around the expected relatedness values and gives more accurate additive genetic variance estimates (Robinson *et al*. 2013). Variance components were estimated with asreml, fitting random and fixed effects as described in Appendix S1 (Supporting information).

Approach ii, in addition to allowing estimation of the population‐specific variances, also enables marker‐based additive genetic variances to be compared between the two populations. To do so, a global matrix of pairwise relatedness was calculated for all genotyped individuals across the two populations. Genotypes were first standardized by the allele frequencies within each population, and the relatedness between all individuals was then calculated following approach 3 in Robinson *et al*. (2013). As above, genomic relatedness estimates were adjusted by known pedigree relationships within the two populations to reduce sampling error. To test whether, for each trait, additive genetic variances differed between the NL and UK, the likelihood of a model where additive genetic variances and the covariance were free to vary was tested against a model where the additive variances and covariance were free to vary but the variances were forced to be equal, with significance tested using a chi‐square with one degree of freedom. For each trait, only fixed and random effects that were measured in both populations were included in the model.

#### Partitioning genetic variation across chromosomes

Chromosome partitioning is an approach to partition the additive genetic variance for complex traits across genomic regions such as individual chromosomes (Visscher *et al*. 2007) and has recently been adapted to data sets with complex pedigrees and close relatives (Robinson *et al*. 2013). A regression of the total variation explained by each chromosome on chromosome size or gene content provides a test for the trait architecture (Robinson *et al*. 2013). A positive regression indicates a polygenic trait architecture, because if many genes contribute to variation, then larger chromosomes with more genes will tend to explain more variation than small chromosomes with fewer genes. In contrast, no relationship between variance explained and chromosome size suggests that the trait is either influenced by a small number of genes of large effect, or that loci having small to moderate effects are not randomly distributed throughout the genome (Robinson *et al*. 2013; Schielzeth & Husby 2014).

Chromosome partitioning was performed as described in Robinson *et al*. (2013) and Santure *et al*. (2013). Given the small number of markers (<60) on some chromosomes, the 5591 ‘chromosome‐assigned’ SNPs were assigned to a total of 22 chromosomes or chromosome sets; chromosomes 1–15, 17–20, 1A and 4A were considered individually (*n* = 21), while a chromosome set was obtained by combining all markers from microchromosomes 21–28 and linkage group LGE22. The number of SNPs in the 22 sets ranged from 98 to 700 (Appendix S3, Supporting information). These regions contained a total of 14 722 genes, predicted from homology with the zebra finch genome (Santure *et al*. 2013; van Oers *et al*. 2014). Genomic relatedness between every pair of individuals was calculated using an approach that scales by the actual variance in relatedness (approach 3; Robinson *et al*. 2013). These genomic estimates of relatedness were then adjusted by the known pedigree relationships between individuals, reducing the sampling error around the expected relatedness values and giving more accurate additive genetic variance estimates (Robinson *et al*. 2013). Variance components were estimated with asreml, using the raw phenotypes and fitting random and fixed effects as described in Appendix S1 (Supporting information). For every chromosome, two mixed models were constructed:

1. With the matrix of marker relatedness between individuals (G matrix) constructed from all markers except those on the focal chromosome.
2. With G constructed from all markers except those on the focal chromosome, plus G constructed with only the markers on that chromosome.

Subsequently, a likelihood ratio test (LRT) was performed for each chromosome to test whether it explained significant variation in the trait, by comparing the log likelihood of the genome‐wide model with the log likelihood of the genome‐wide plus chromosome model.

Finally, linear regressions were fitted between the variance explained by each chromosome and the number of genes they contained, to test whether traits were influenced by a large number of loci distributed throughout the genome.

#### Quantitative trait locus analysis

A two‐step variance components analysis (George *et al*. 2000; Slate *et al*. 2002) was performed to map loci contributing to variance in the quantitative traits. Using the 1524 markers in the framework without LD map, the identical‐by‐descent (IBD) coefficients between all pairs of individuals in the two QTL pedigrees (see above) were derived at 5‐cM intervals across the genome using the software loki v2.4.5 (Heath 1997; Heath *et al*. 1997), with 100 000 iterations for each position. The statistical significance of the QTL effects was then tested using a likelihood ratio test comparing the log likelihood of a polygenic model with the log likelihood of a polygenic plus QTL model, fitting full mixed models to account for the effects of important random and fixed effects on the raw phenotypes (see Appendix S1, Supporting information). QTL scans were performed in the two populations for each trait. To account for the multiple tests performed genome‐wide, the approach of Lander & Kruglyak (1995) was used to adjust the significance thresholds based on the length of the linkage map (19.16 morgans) and number of mapped chromosomes (31). For these data sets, a logarithm of odds (LOD) score (where LOD = LRT/2*ln*(10)) of 1.620, corresponding to a nominal *P* value (*P*) of 0.003, is expected to occur once by chance in every genome scan and is termed ‘genome‐wide suggestive linkage’, while a LOD score of 3.062 (*P *=* *9 × 10^−5^) is expected with probability 0.05 every time a genome scan is performed and is termed ‘genome‐wide significant linkage’ (Lander & Kruglyak 1995; Nyholt 2000). Nominal significance (*P *<* *0.05) requires a LOD score of ≥0.588.

To test whether the same regions of the genome explain trait variation in the two populations, the correspondence between the genome‐wide test statistics across populations obtained from the QTL scans was tested using the permutation approach of Keightley & Knott (1999) (Appendix S4, Supporting information). In brief, the approach first calculates the correlation in test statistics between two QTL scans. Test statistics are then permuted while maintaining the autocorrelation between linked test statistic values, that is whole chromosomes (rather than individual sites) from one QTL scan are permuted across the other QTL scan. The significance of the observed correlation is tested against the distribution of correlations from the permuted data sets. Regions of the genome that were nominally significant in both populations were also identified.

#### QTL mapping power analysis

The ability to draw conclusions from a linkage mapping analysis depends on the power to detect QTL of various effect sizes, and knowledge about potential bias in reported effect sizes (Slate 2013). Following Slate (2013) and Santure *et al*. (2013), a simulation approach was used to determine the power to detect QTL explaining 0% (i.e. false detection of QTL), 5%, 10%, 20% and 40% of the overall phenotypic variance for each trait in each population (Appendix S5, Supporting information). The simulations were also used to assess the amount of bias in reported effect sizes that may be expected at various significance thresholds in the two populations.

#### Genome‐wide association study

To test whether individual SNPs explained significant variation in each of the traits, a genome‐wide association study was conducted. First, a univariate mixed model including all fixed and random effects but excluding additive genetic effects was fitted in asreml, to give an expected phenotypic value, or EPV, for each individual (Ekine *et al*. 2014). EPVs for each trait were then standardized to mean 0 and variance 1. This approach was employed to reduce phenotypes to a single value for each individual (rather than repeated measures on each individual), and to account for other important fixed and random effects (Appendix S1, Supporting information), except for the genetic differences between individuals that might associate with the phenotype.

Within each population, SNPs were tested for allelic association with each EPV (i.e. models did not include previously controlled for fixed and random effects from the model) using the ‘polygenic’ and ‘mmscore’ functions in genabel (Aulchenko *et al*. 2007b), adjusting for population stratification due to the presence of related individuals by fitting the internally calculated genome‐wide kinship matrix as a random effect (Amin *et al*. 2007). The significance of association was assessed using genabel *P* values, adjusted for multiple testing using a Bonferroni correction based on the effective number of independent tests. Taking into account LD between markers using the package Keffective (Moskvina & Schmidt 2008), the panel of chromosome‐assigned SNPs was determined to yield 5573 effective tests, giving a genome‐wide significant threshold of *P *=* *9.0 × 10^−6^.

The between‐population correlation of absolute estimated SNP effect sizes from the GWAS analyses was calculated in r (R Development Core Team 2012) for each trait. A significant correlation in SNP effect sizes between a NL and UK trait would indicate that many shared loci contribute to variation in the two populations. The significance of each correlation was tested by permuting effect sizes across SNPs 10 000 times.

Finally, for each trait, standardized EPVs and genotypes from each population were merged to test for association across both data sets. SNP association was tested by running a ‘polygenic’ mixed model in genabel, where the genome‐wide kinship matrix is fitted as a random effect, population fitted as a fixed effect and the first 10 principle components of the kinship matrix fitted as fixed effects to account for population‐level SNP relatedness differences. The significance of association was assessed from ‘mmscore’ *P* values after Bonferroni correction.

#### Concordance between GWAS, QTL mapping and chromosome partitioning

The QTL mapping and GWAS approaches described above exploit different marker and phenotype information, with different numbers of individuals and markers used. QTL mapping relies on recombination events within families to define the ‘boundaries’ around a causal locus. In contrast, GWAS analysis exploits ancestral recombination events, which have broken down associations between markers and phenotype for all loci except those in close physical linkage to causal loci. Because the power to detect QTL by linkage or association is dependent on the QTL magnitude (Lynch & Walsh 1998; Sham *et al*. 2000) and the power of GWAS is additionally dependent on the amount of LD between causal variants and the markers (Pritchard & Przeworski 2001), QTL mapping and GWAS results may not necessarily be concordant. Genome‐wide significance of both QTL mapping and GWAS at the same region of the genome would give clear support for a QTL at that location. However, if both approaches do not reach genome‐wide significance but, for example, a position was nominally significant in GWAS (*P *<* *0.05) and reached genome‐wide suggestive linkage in the QTL mapping (LOD > 1.620, *P *<* *0.003), this would add some support for the presence of a QTL at that location.

Within each population, the concordance between the (i) QTL mapping and GWAS analyses and (ii) QTL mapping and chromosome partitioning analysis was tested for each trait. First (i), the LOD score at the mapping position (in cM) of each SNP marker was predicted from a linear regression of the LOD scores of neighbouring QTL positions (e.g. LOD scores at 0 cM and 5 cM were used to predict the LOD score of a SNP mapped to 4.6 cM), and the *P* value for each inferred LOD score was calculated and compared to the *P* value from the GWAS. To determine whether the number of genome positions nominally significant in both analyses was greater than expected by chance, the observed and expected counts were compared with a chi‐square test. Second (ii), those chromosomes with nominally significant (*P *<* *0.05) QTL mapping peaks were compared with the nominally significant chromosomes from the partitioning approach with a Fisher's exact test (i.e. with counts of nominal significance in both, one or neither approach). If a small number of loci of major effect contribute to the trait, it might be expected that all three approaches are concordant, especially if major genes are located on small chromosomes.

#### Estimating the number of SNPs contributing to variance

QTL mapping and GWAS aim to identify loci that make a large contribution to trait variation, but do not provide an estimate for the total number of loci contributing to phenotypic variation. The number of SNPs explaining trait variation and the overall phenotypic variance explained by the SNPs were estimated using a Bayesian variable selection regression model (Guan & Stephens 2011) implemented in the software pimass (http://www.haplotype.org/pimass.html). This approach fits a linear model where phenotype is determined by a subset of SNPs, each of which have an estimated effect size on the phenotype. In each iteration, SNPs may be kept, added or removed from the model, with SNPs with strongest associations with the phenotype added to the model with highest probability. The total number of SNPs contributing to the phenotype, and the total amount of variation explained by these SNPs, is calculated from the model at each iteration and defines the posterior distributions on these parameters. While this approach does not explicitly account for population stratification due to the presence of related individuals, the likelihood of false‐positive associations between SNPs and traits caused by this population structure is reduced by estimating SNP effect sizes after controlling for other SNPs in the model (some of which may already account for population structure) (Guan & Stephens 2011; Gompert *et al*. 2013).

The multi‐SNP analysis was performed on EPVs for each trait using pimass to obtain MCMC samples from the joint posterior probability distribution of the model parameters. For each trait, the model was run three times for 110 000 iterations, with parameter values recorded every 10th iteration after discarding the first 10 000 iterations. Results were similar across replicate runs; therefore, results (median values, mean values and 95% equal tail probability credible intervals) are presented across all three replicates for all but two traits, where poor convergence was observed in one replicate, and hence, results are presented for the remaining two replicates.

The estimated number of SNPs contributing to trait variance, and the total proportion of variance explained, was compared for each trait between the two populations, by determining whether 95% credible intervals on the estimates overlapped.

## Results

### Pedigree‐ and marker‐based heritabilities

In agreement with previous studies, there was evidence that all traits were heritable as estimated using either or both pedigree‐ or marker‐based relatedness to partition the additive genetic variance (Table 2; although note that the marker‐based estimate for UK fledgling weight (of individual) when all available random and fixed effects were fitted did not differ significantly from zero). Although pedigree‐ and marker‐based heritabilities were reasonably consistent across and between populations, there were some large differences between estimates in some cases. For example, the UK marker‐based heritability estimate of fledgling weight (of individual) is nearly 0.5 lower than the UK pedigree value when fitting all available random and fixed effects. To test for population‐specific heritabilities, the NL and UK data sets were merged and marker‐based heritabilities estimated, fitting only random and fixed effects measured in both populations. Fledgling weight (of individual) was the only trait for which the marker‐based additive genetic variance significantly differed between populations (Table 2).

**Table 2:** NL and UK heritabilities for great tit quantitative traits. In each cell, NL parameter estimates are shown first, with standard errors shown in parentheses. Note that pedigree‐based heritabilities are estimated using QTL mapping individuals, while marker‐based heritabilities are estimated from all GWAS/chromosome partitioning phenotyped individuals (see Table 1)

	Pedigree‐based heritabilities, full model^b^	Marker‐based heritabilities, full model^b^	Marker‐based heritabilities, restricted model^c^	Significant difference in marker‐based additive genetic variances^c^
Clutch size	0.483 (0.043)^a^	0.237 (0.066)^a^	0.297 (0.086)^a^	No
	0.395 (0.088)^a^	0.424 (0.079)^a^	0.390 (0.075)^a^	
Egg mass	—	—	N/A	N/A
	0.396 (0.042)^a^	0.424 (0.036)^a^		
Fledgling weight (of offspring)	0.376 (0.088)^a^	0.389 (0.091)^a^	0.161 (0.093)^a^	No
	0.365 (0.126)^a^	0.237 (0.108)^a^	0.506 (0.126)^a^	
Adult weight	0.454 (0.086)^a^	0.285 (0.075)^a^	0.298 (0.083)^a^	No
	0.394 (0.042)^a^	0.345 (0.037)^a^	0.381 (0.038)^a^	
Fledgling weight (of individual)	0.698 (0.175)^a^	0.604 (0.120)^a^	0.646 (0.113)^a^	Yes
	0.595 (0.060)^a^	0.114 (0.072)	0.412 (0.066)^a^	
Tarsus length	0.592 (0.073)^a^	0.301 (0.054)^a^	0.630 (0.066)^a^	No
	0.632 (0.072)^a^	0.246 (0.049)^a^	0.571 (0.066)^a^	
Wing length	0.353 (0.081)^a^	0.270 (0.076)^a^	0.316 (0.066)^a^	No
	0.568 (0.040)^a^	0.511 (0.040)^a^	0.520 (0.040)^a^	
Exploratory behaviour	0.284 (0.103)^a^	0.140 (0.066)^a^	0.272 (0.097)^a^	No
	0.185 (0.088)^a^	0.263 (0.080)^a^	0.260 (0.081)^a^	

a *P *<* *0.05 (LRT of >2.706).

b Model where all available fixed and random effects were fitted.

c Model where fixed and random effects were fitted only if they were available for both the NL and UK population.

### Partitioning genetic variation across chromosomes

**Figure 1:**
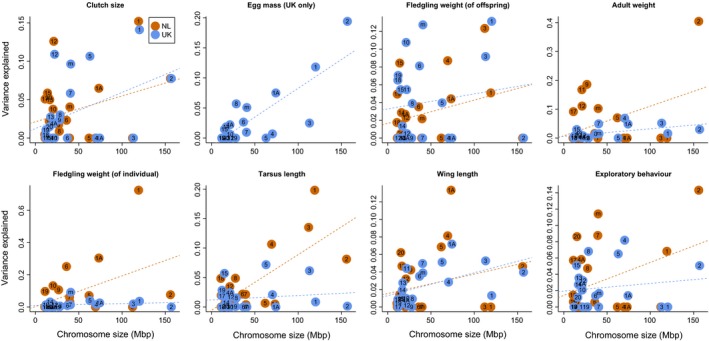
Relationship between chromosome size (Mbp) and variance explained for maternal, morphological and behaviour traits for the NL and UK populations.

For all traits, there was a positive relationship between variance explained and chromosome size (Fig. 1), with a significant relationship (*P *<* *0.05) for fledgling weight (of individual) and tarsus length in the NL population and for clutch size, egg mass, adult weight and wing length in the UK population (Table 3). There were three instances where chromosomes that explained significant variation in one population explained significant variation in the other (Fig. S1, Supporting information); chromosome 12 explained significant variation in both NL and UK clutch size, chromosome 3 explained significant variation in both NL and UK tarsus length, while chromosome 1A explained significant variation in both NL and UK wing length (details for the amount of variation explained by each chromosome, and their significance, are provided in Appendix S3, Supporting information). In addition, chromosome 1 explained a reasonably large proportion of variation in clutch size in both populations, although the proportion was not significant in the NL population (Fig. 1; Fig. S1, Supporting information). However, the three instances of chromosomes explaining significant variation in both populations were not a greater number than expected by chance (binomial test, *P *=* *0.188 for three or more shared chromosomes).

**Table 3:** Relationship between the proportion of variance explained by each chromosome and chromosome size (Mbp) for traits in the NL and UK populations

Trait	NL			UK		
	*R* ^2^	Slope (× 10^−4^)	*P*	*R* ^2^	Slope (× 10^−4^)	*P*
Clutch size	0.104	3.359	0.143	0.182	4.701	0.048^a^
Egg mass	—	—	—	0.648	9.559	0.000^a^
Fledgling weight (of offspring)	0.096	2.620	0.160	0.023	1.693	0.505
Adult weight	0.177	10.268	0.051	0.271	2.615	0.013^a^
Fledgling weight (of individual)	0.214	19.050	0.030^a^	0.046	1.131	0.336
Tarsus length	0.536	9.430	0.000^a^	0.019	0.747	0.540
Wing length	0.062	2.217	0.263	0.257	2.616	0.016^a^
Exploratory behaviour	0.137	4.045	0.090	0.022	0.941	0.510

a *P *<* *0.05.

### QTL analysis

**Figure 2:**
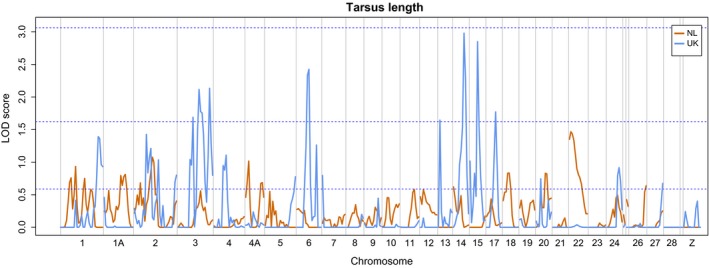
QTL scans for tarsus length in the NL and UK populations. Blue dashed lines show nominal (LOD = 0.588), suggestive (LOD = 1.620) and significant (LOD = 3.062) scores. Chromosome labels are shown beneath the plots; chromosomes 25A and 25B (plotted after chromosome 24) and LGE22 (after 28) are not labelled.

For six of the seven NL traits and all of the eight UK traits, no regions of the genome reached genome‐wide significance (Fig. S2a–h; Appendix S6, Supporting information). There were three genome‐wide significant peaks for NL adult weight, on chromosomes 11, 15 and 28 (Fig. S2d, Supporting information), accounting for 96%, 100% and 100% of the heritability, respectively, when estimated individually, clearly demonstrating the overestimation of effect sizes that reach significance. Fitting all three QTL in the same model accounted for 100% of total heritability, with chromosomes 11, 15 and 28 accounting for 27%, 35% and 38% of heritability, respectively. There were a relatively large number of suggestive peaks for NL and UK adult weight (peaks on 13 chromosomes in the NL and on four chromosomes in the UK) and for UK tarsus length (peaks on a total of six chromosomes, Fig. 2). One false‐positive genome‐wide suggestive peak is expected by chance every time a genome scan is performed, and hence, the excess of suggestive peaks in these traits suggests that while some are likely to be false positives, others may be true QTL that have failed to reach genome significance. All suggestive positions, along with their LOD scores and estimated effect sizes, are provided in Appendix S6 (Supporting information).

There was no evidence of correspondence between the QTL test statistics for each trait obtained from the NL and UK QTL scans (including adult weight). Correlations of test statistics between the populations ranged from −0.083 to 0.175, with all but two negative, and none were significant when tested using the approach of Keightley & Knott (1999) (Appendix S4 and Fig. S3a–g, Supporting information). A number of traits shared nominally significant QTL peaks; eleven genomic regions for adult weight were shared between the NL and UK, two regions were shared for fledgling weight (of individual), one region was shared for tarsus length, and two regions were shared for wing length (Appendix S7, Supporting information).

### QTL mapping power analysis

The QTL power analysis (Appendix S5, Supporting information) suggests that for all NL traits, there was very little power to detect QTL of major effect, with less than 20% of QTL simulated to explain *all* additive genetic variance detected at genome‐wide significance in the highest powered data sets (tarsus and wing length) and close to 0% detected in other traits. At the suggestive linkage threshold, the number of detected QTL that were simulated to explain all additive genetic variance ranged from 2% for exploratory behaviour to 54% for tarsus length. For the UK data set, close to 100% of QTL simulated to explain all additive genetic variance were detected at genome‐wide significance for adult weight, fledgling weight (of individual) and wing length, although very few were detected for fledgling weight (of offspring) and exploratory behaviour (0% and 3%, respectively). At the suggestive threshold, 21% and 26% of QTL explaining all additive genetic variance were detected in the fledgling weight (of offspring) and exploratory behaviour simulations. There is unlikely to be power to detect QTL of more reasonable effect sizes in either the NL or UK data sets; QTL simulated to explain 5% of phenotypic variation were never detected in the NL simulations and were rarely detected in the UK simulations. As expected, the estimated effect sizes for QTL detected in the simulations were highly inflated (Beavis 1994; Slate 2013). Power to detect QTL was dependent on QTL effect size and the number of individuals (*P *<* *0.001 in both cases; tested using an analysis of variance with the lm function in r (R Development Core Team 2012); other effects including trait heritability, population, the number of trait records and whether traits were maternal or individual were not significant. Thus, QTL mapping is only likely to detect loci of very large effect, and even then, interpretation of effect sizes is problematic.

### GWAS

None of the 5591 SNPs tested for association with any of the seven NL or eight UK traits reached genome‐wide significance after adjustment for multiple testing (Fig. S4a–h, Supporting information). There was no evidence for some chromosomes having more nominally significant SNPs than others (chi‐square test on the observed and expected counts of nominally significant SNPs per chromosome, *P *>* *0.05 for all traits).

The correlation between absolute estimated SNP effect sizes from the GWAS analysis was estimated between the NL and UK for each trait (Table 4, Fig. S5, Supporting information). If many shared loci contribute to variation in the two populations and these loci are tagged by the genotyped SNPs, then the same SNPs will have similar effects in both populations, and a significant correlation between SNP effect sizes in the NL and UK would be expected. However, such correlations were not observed, suggesting that either the same SNP markers are not tagging the same causal variants in the two populations (either because there are different causal variants in each population, or perhaps due to differences in LD structure) or, perhaps more likely, that there is limited LD between genotyped SNPs and causal variants in both populations, and hence, SNP effect sizes are generally small and inaccurately estimated. In addition, no SNPs were significantly associated with any of the traits when UK and NL data sets were merged, suggesting that despite increased power, no SNPs had large enough effect across the two data sets to reach genome‐wide significance (Fig. S4a–h, Supporting information).

**Table 4:** Interpopulation correlations between GWAS‐estimated effect sizes per SNP for each trait; none are significant after adjusting for multiple testing

Trait	Correlation	*P*
Clutch size	0.017	0.115
Fledgling weight (of offspring)	0.009	0.254
Adult weight	0.000	0.333
Fledgling weight (of individual)	−0.020	0.068
Tarsus length	−0.029	0.018
Wing length	0.000	0.135
Exploratory behaviour	−0.009	0.257

### Concordance between GWAS, QTL mapping and chromosome partitioning

For all traits in both the UK and NL, there was no significant excess of positions in the genome that were nominally significant in both the QTL and GWAS analyses (chi‐square test on the observed and expected counts of SNPs being nominally significant in both, one or neither the GWAS nor QTL analysis, see Appendix S8, Supporting information for *P* values). There was no evidence that chromosomes that contributed significantly to overall variance (measured as the significance of the LRT, see Appendix S3, Supporting information) were more likely to harbour nominally significant QTL peaks (detected from the QTL mapping approach), compared to chromosomes that did not contribute significantly to overall variance [two‐tailed Fisher's exact test, *P *>* *0.05 for all traits except UK clutch size *P *=* *0.046 (not significant after accounting for multiple testing)].

### Estimating the number of SNPs contributing to variance

There was good agreement in the SNP estimated effect sizes from the GWAS and from the multi‐SNP analysis (Appendix S9, Supporting information), suggesting that the multi‐SNP analysis was not biased by within‐population structure. Estimates for the proportion of variance explained (PVE) differed considerably between traits and between populations in the multi‐SNP association analysis (Table 5), ranging from 0.034 to 0.581. For all traits, the number of SNPs contributing to variation was considerable, with median values ranging from 31 to 310 (Table 5).

**Table 5:** Median proportion of variance explained (PVE) and median number of SNPs (nSNP) explaining trait variation, predicted by the multi‐SNP association analysis

Trait	NL		UK	
	PVE	nSNP	PVE	nSNP
Clutch size	0.141 (0.015, 0.251)	169 (5, 304)	0.646 (0.559, 0.721)	310 (272, 349)
Egg mass	—	—	0.720 (0.619, 0.773)	309 (271, 342)
Fledgling weight (of offspring)	0.034 (0.000, 0.159)	31 (0, 160)	0.086 (0.000, 0.322)	60 (0, 252)
Adult weight	0.394 (0.232, 0.557)	163 (45, 288)	0.279 (0.217, 0.335)	288 (208, 330)
Fledgling weight (of individual)	0.581 (0.393, 0.754)	241 (124, 313)	0.312 (0.225, 0.398)	245 (164, 310)
Tarsus length	0.196 (0.107, 0.291)	243 (109, 309)	0.354 (0.228, 0.477)	269 (168, 330)
Wing length	0.131 (0.039, 0.231)	238 (25, 306)	0.350 (0.256, 0.408)	297 (208, 339)
Exploratory behaviour	0.157 (0.048, 0.257)	94 (26, 255)	0.162 (0.065, 0.252)	200 (87, 295)

Numbers in parentheses are 95% credible intervals.

**Figure 3:**
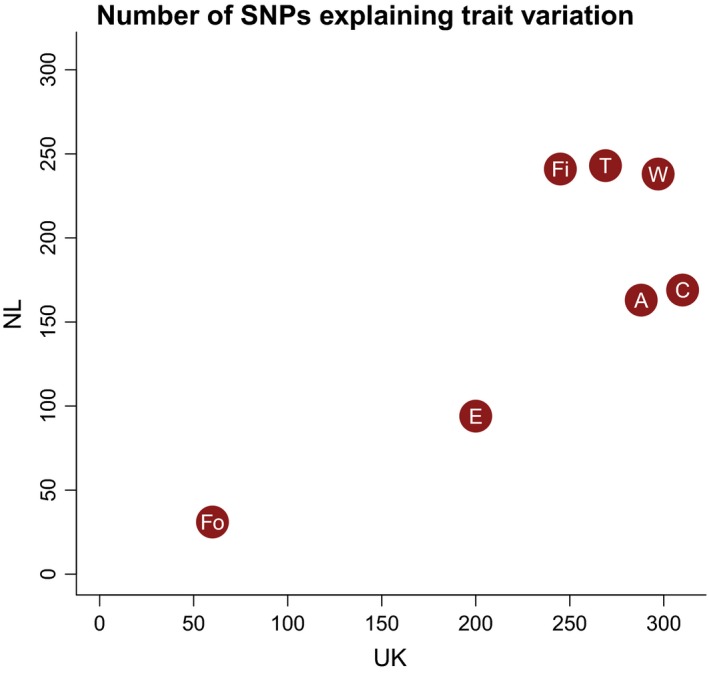
The estimated median number of SNPs explaining trait variation in the NL and UK populations. Trait abbreviations: C = clutch size, Fo = fledgling weight (of offspring), A = adult weight, Fi = fledgling weight (of individual), T = tarsus length, W = wing length, E = exploratory behaviour.

The median number of SNPs explaining variance was lower in the NL, but the credible intervals were large and overlapping for all pairs of traits between the NL and UK (Table 5). There was a significantly positive correlation (*P *=* *0.016) between the estimated median number of SNPs explaining variance in the two populations (Fig. 3). The proportion of phenotypic variance explained by the SNPs differed between populations for clutch size and wing length, with estimates being lower in the NL than in the UK (Table 5).

## Discussion

### Evidence that traits are polygenic

The architecture of maternal, morphological and behaviour traits in two natural populations of great tits was investigated using chromosome partitioning, QTL mapping, GWAS and by estimating the number of SNPs contributing to variation. All four approaches lend some support to the hypothesis that the quantitative traits studied here are influenced by many genes of small effect distributed throughout the genome.

Although not all regressions were significant, it is notable that the relationship between chromosome size and variance explained was positive for all traits in both populations (Table 3, Fig. 1), with no evidence in any trait that a gene of major effect contributes to a large proportion of trait variance (see simulations in Robinson *et al*. 2013). The variation in the relationship between chromosome size and variance explained for each trait is likely to be a consequence of our marker density; more markers in stronger linkage disequilibrium with each other would better capture the true relatedness between individuals across a chromosome and would reduce the error on the estimates for the contribution of each chromosome. However, the chromosome partitioning results are generally consistent with the traits having a polygenic rather than oligogenic architecture; in every trait, more than a handful of chromosomes explain at least some variation.

For the majority of traits, the QTL analyses failed to detect any regions of the genome contributing significantly to trait variation. There was evidence that specific regions of the genome contributed significantly to variation in adult weight in the NL population; however, it is notable that there was no overall similarity in test statistics between the two populations, and there is no agreement between chromosome partitioning, GWAS and QTL analysis within the NL population. It should be noted, however, that the QTL analysis may be identifying QTL that are not in LD with any of the genotyped SNPs and hence not likely to show a signal for association; a higher SNP density in these regions of the genome may be able to dissect the true contribution of these loci to quantitative trait variation. Given the inevitable overestimation of QTL effect sizes (Beavis 1994; Slate 2013), the estimates for the variation explained by each QTL peak (27%, 35% and 38% of the total heritability for peaks on chromosomes 11, 15 and 28, respectively, when all three are included in the same model) are unlikely to reflect the true contribution of these QTL to adult weight. Furthermore, the chromosome partitioning analysis lends no support for loci of large effect to be located on the chromosomes with significant QTL peaks; in the NL population, chromosomes 11, 15 and 28 contain significant QTL peaks, but only one of these, chromosome 11, explained significant variation in the chromosome partitioning analysis. There was no excess of nominally significant peaks from the NL GWAS analysis on the QTL‐significant chromosomes, and the estimates for the number of SNPs explaining variation were 163 and 288 SNPs for the NL and UK, respectively. Therefore, despite the results from the QTL linkage analysis, there appears to be little supporting evidence from partly independent analyses that loci of major effect contribute to adult weight variation in either population. In the absence of genome‐wide significant results, and a lack of support from the GWAS or chromosome partitioning analyses, we suggest that the QTL that are significant at the suggestive threshold are very likely to be overestimated and that some may represent false‐positive associations.

Overall, our results lend support to the hypothesis that quantitative trait architecture is likely to be determined by many loci of small effect distributed throughout the genome, agreeing with recent conclusions from studies in humans, livestock and model organisms (see, for example, Mackay *et al*. 2009; Hayes *et al*. 2010; Yang *et al*. 2011) and other wild populations (Bérénos *et al*. 2015; Husby *et al*. 2015).

### Do different populations share a common genetic architecture?

Given that these traits appear to be influenced by a very large number of genes and that the two study populations have very little genetic differentiation (global *F*_ST_ = 0.01; minor allele frequency correlation = 0.98, van Bers *et al*. 2012), it seems reasonable to ask whether the genetic architectures are shared to some degree between populations. The observation that additive genetic variances between traits are not significantly different between populations for all traits except fledgling weight (of individual) lends some support to this hypothesis. In addition, the number of SNPs estimated to contribute to variation for each trait was similar in the two populations. However, regions of the genome that appeared to explain variation in one population did not explain significant variation in the other population more often than expected by chance. There are several possible explanations. One is that neither population has sufficient power to identify causal loci nor to assign statistical significance to chromosomal contributions to variance. Therefore even if the same loci (or a subset of the causal loci) contribute to trait variation in both populations, the probability of detecting an overlap of significant results is low. Alternatively, different loci could contribute to trait variation in both populations; the data are not inconsistent with any given trait having a polygenic architecture caused by different loci in different populations, although given that allele frequencies are highly correlated between populations (van Bers *et al*. 2012), we regard this as unlikely. Although alternative explanations are possible, we suggest that most of the traits studied here are highly polygenic, and it is likely they share, at least in part, a common genetic architecture.

### Prospects for molecular quantitative genetics in wild populations

The results of four complementary approaches in two populations lend support to the hypothesis that all eight traits are polygenic. However, our efforts highlight the lack of power even a study of this size (within population, between 416 and 1949 phenotyped individuals genotyped at 5591 SNPs) may have to dissect the genetic architecture of quantitative traits. In particular, it is now becoming clear that a QTL linkage analysis approach suffers from very limited power in pedigreed wild populations (Santure *et al*. 2013; Slate 2013; and data presented in this manuscript) and will in most cases only allow the identification of genes of very large effect sizes. Our simulation results (Appendix S5, Supporting information) indicate that for most traits, it would be impossible to make robust conclusions about trait architectures from the QTL results alone. This low power is most strongly influenced by the limited numbers of phenotyped individuals available in such populations (as illustrated by the power analysis, Appendix S5, Supporting information), but is also likely to be affected by the structure of the pedigree. For example, short lifespans and high fledgling mortality in great tits mean that there are likely to be relatively few close relative pairs where IBD can be accurately inferred (compared to, for example, longer‐lived species where half‐ or full‐sib families are more common).

Similarly, although the data set currently represents one of the largest genomic data sets for a wild population, LD between the SNP markers is low (I. De Cauwer, A. W. Santure, K. van Oers, N. E. M. van Bers, R. P. M. A. Crooijmans, B. C. Sheldon, M. E. Visser, M. A. M. Groenen & J. Slate, unpublished data), suggesting that the set of SNP markers provides insufficient power to detect causal variants using genome‐wide association scans. The fact that for many traits the proportion of phenotypic variance explained by the SNPs is substantially lower than the heritability supports the hypothesis that the genome is not adequately tagged by the current set of SNPs. Identifying loci contributing to trait variation, and testing for shared genetic architectures in these populations, will therefore likely require many more markers. With that in mind, we would advise researchers to work towards the development of SNP data sets that will allow GWAS, chromosome partitioning and other molecular quantitative genetic analyses, which are likely to enable much finer‐scale dissection of genetic architectures and trade‐offs than will be possible from QTL mapping. SNP data sets of ~37 000 SNPs (after quality control) enabled the identification of a handful of loci influencing clutch size in collared flycatchers (Husby *et al*. 2015) and leg length traits in Soay sheep (Bérénos *et al*. 2015), although it should be noted that these loci explain a minor proportion of total heritability. Further, the use of genomic relatedness is likely to offer a promising approach for estimating heritabilities in wild populations when pedigrees may be incomplete or incorrect, and may also allow for more accurate estimation of maternal effects (Bérénos *et al*. 2014). For some species such as great tits, which have a very large effective population size and correspondingly low levels of LD, describing the genetic architecture of polygenic traits likely means genotyping many hundreds of thousands of loci. Perhaps more soberingly, regardless of LD, accurate inferences about the genetic architecture of quantitative traits in wild populations may require phenotypic data from considerably larger numbers of individuals than will be possible to sample under most circumstances, and in many cases, the availability of phenotypic data for analysis will depend on decisions taken by investigators in the past.

A.W.S. performed the heritability analysis, chromosome partitioning, QTL mapping, GWAS and multi‐SNP analysis and drafted the manuscript. J.P. performed the QTL power analysis. J.P. and J.S. contributed to manuscript writing with A.W.S., and all authors provided comments on the manuscript. A.W.S., I.D.C., J.P. and M.R.R. prepared the phenotypic and genetic data sets for analysis. B.C.S., K.vO., M.E.V., M.A.M.G. and J.L.Q. provided the phenotypic data and the great tit blood samples. The study was performed as part a collaborative project between J.S., B.C.S., M.E.V. and M.A.M.G. A.W.S., J.S., M.R.R. and J.P. designed the study. All authors read and approved the final manuscript.

## Data accessibility

SNP genotypes, cM locations for all SNP markers, the framework linkage map used in QTL mapping, pedigree information, phenotype files including fixed and random effects and GWAS results are available for both populations in the DRYAD entry doi:10.5061/dryad.5t32v.

## Supporting information


**Fig. S1** Relationship between variance explained by each chromosome for maternal, morphological and personality traits in the NL and UK populations.
**Fig. S2** (a–h) QTL scans for the quantitative traits in the NL (orange) and UK (blue) populations.
**Fig. S3** (a–g) Null distribution of correlations between QTL LOD scores between the UK and NL for each trait.
**Fig. S4** (a–h) GWAS plots for the quantitative traits in the NL and UK populations.
**Fig. S5** Plots of estimated effect size for each SNP in the UK and NL populations.

**Appendix S1** Important fixed and random effects fitted for each trait.
**Appendix S2** Resolving the social pedigree.
**Appendix S3** Contributions of individual chromosomes to heritability of traits in the NL and UK.
**Appendix S4** Correspondence between the test statistics obtained from the QTL scans.
**Appendix S5** QTL mapping power analysis.
**Appendix S6** Suggestive QTL peaks.
**Appendix S7** Concordance of nominally significant QTL peaks between traits.
**Appendix S8** The concordance between QTL mapping and GWAS results for each trait within each population.
**Appendix S9** Concordance of estimated effect sizes for all SNPs from GWAS and multi‐SNP association analyses.
