# Supplementary material for: Replicated analysis of the genetic architecture of quantitative traits in two wild great tit populations
Source: Mol Ecol. 2015 Dec 10;24(24):6148–62. doi: 10.1111/mec.13452 (PMC4738425; doi:10.1111/mec.13452)

Supporting Figure 1

Relationship between variance explained by each chromosome for maternal, morphological and personality traits in the NL and UK populations.

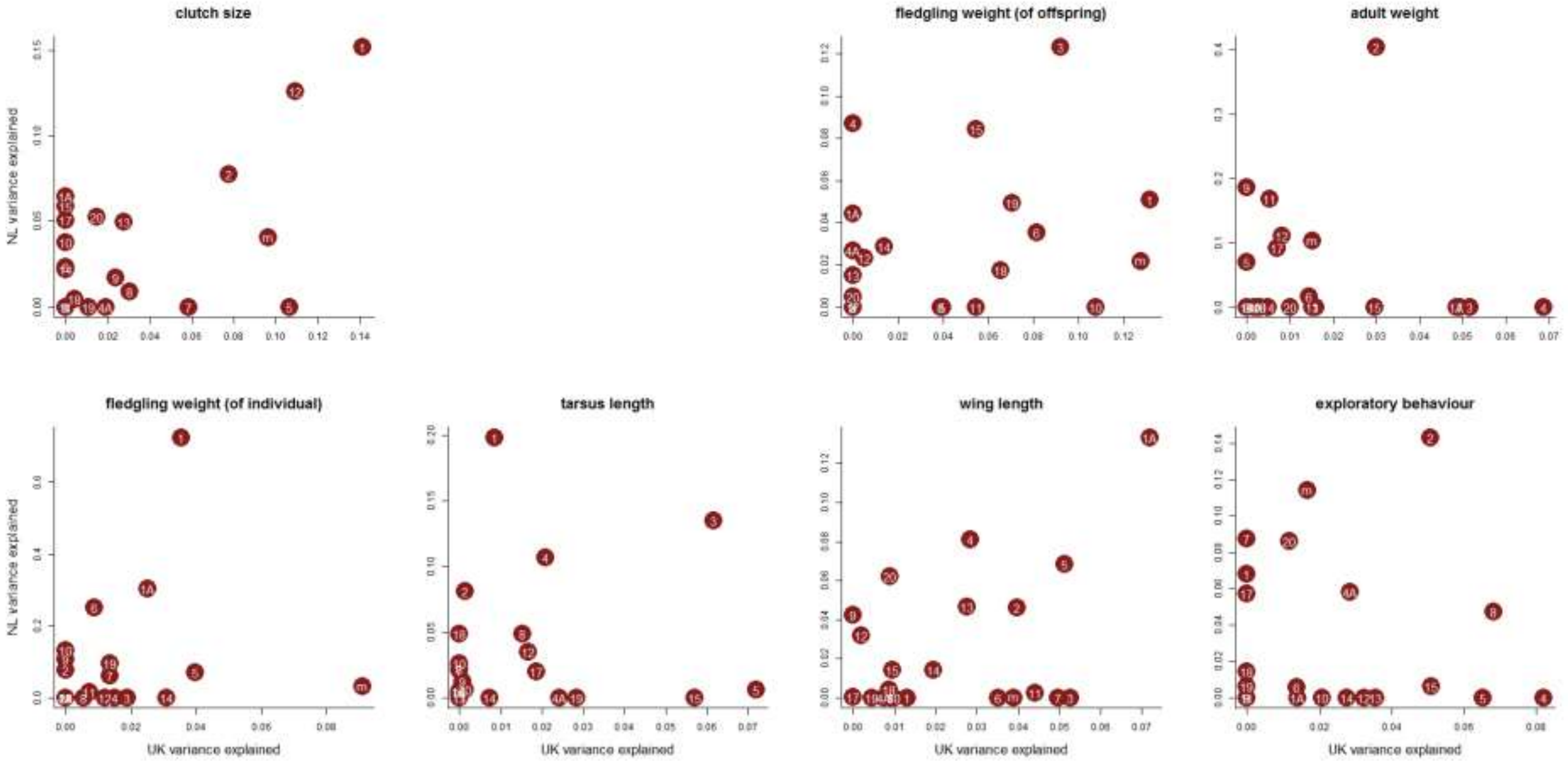

Supporting Figure 2

Figures S3a-S3h: QTL scans for the quantitative traits in the NL and UK populations. Dashed blue lines show nominal (LOD = 0.588), suggestive (LOD = 1.620) and significant (LOD = 3.062) scores. Chromosome labels are shown beneath the plots; chromosomes 25A and 25B (plotted after chromosome 24) and LGE22 (after 28) are not labelled.

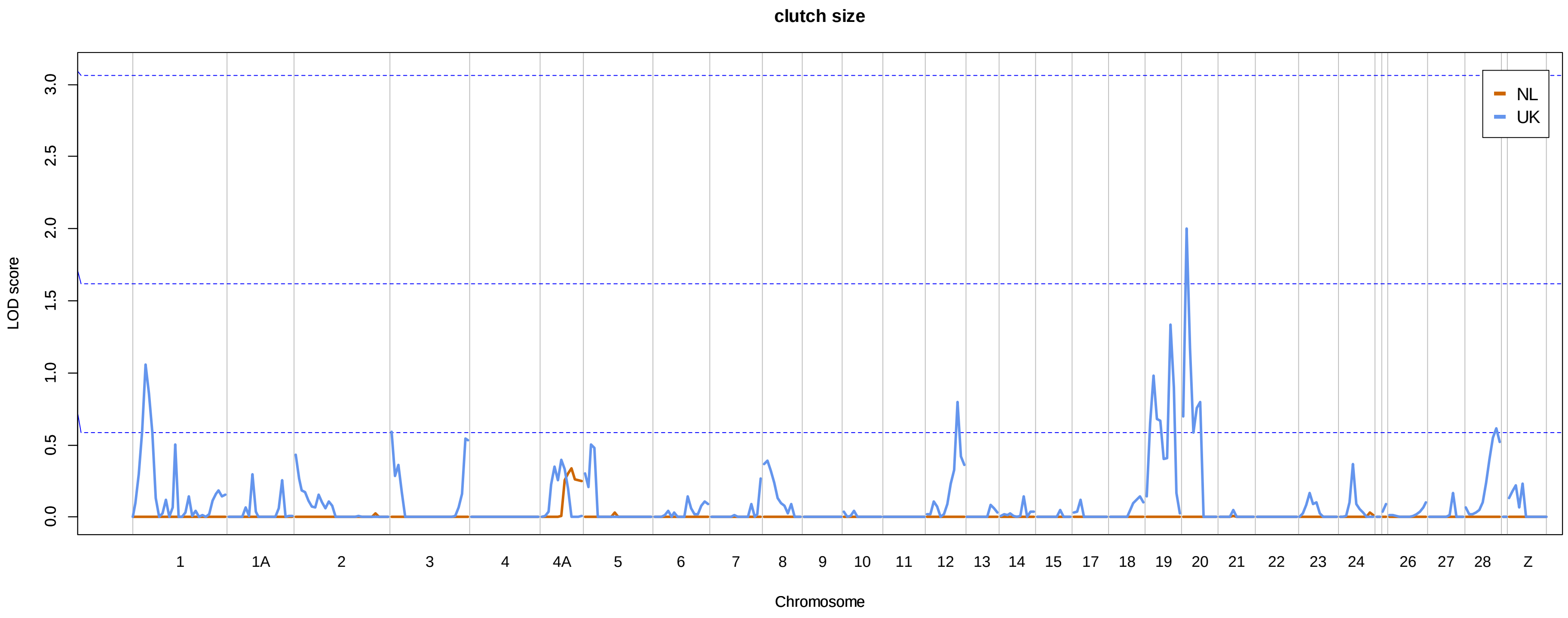

clutch size
LOD score
3.0
2.5
2.0
1.5
1.0
0.5
0.0
NL
UK
1
1A
2
3
4
4A
5
6
7
8
9
10
11
12
13
14
15
17
18
19
20
21
22
23
24
26
27
28
Z
Chromosome

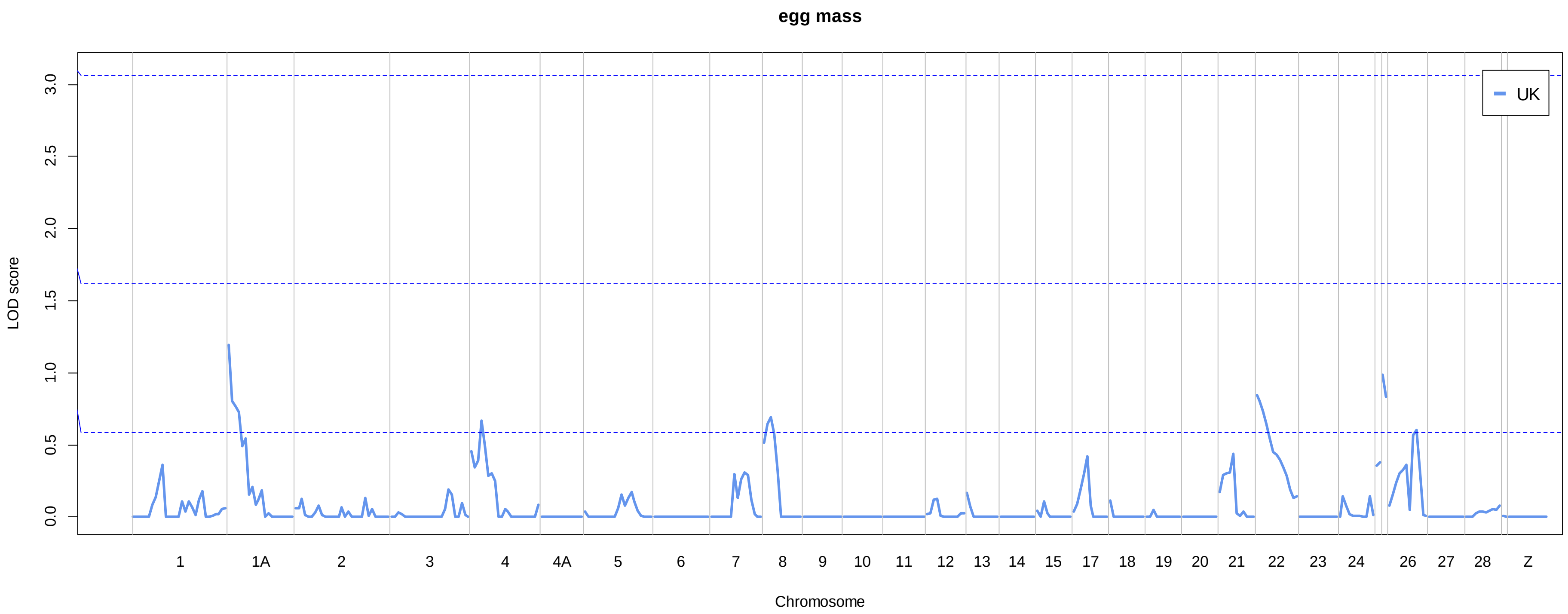
egg mass
LOD score
0.0
0.5
1.0
1.5
2.0
2.5
3.0
UK
1
1A
2
3
4
4A
5
6
7
8
9
10
11
12
13
14
15
17
18
19
20
21
22
23
24
26
27
28
Z
Chromosome

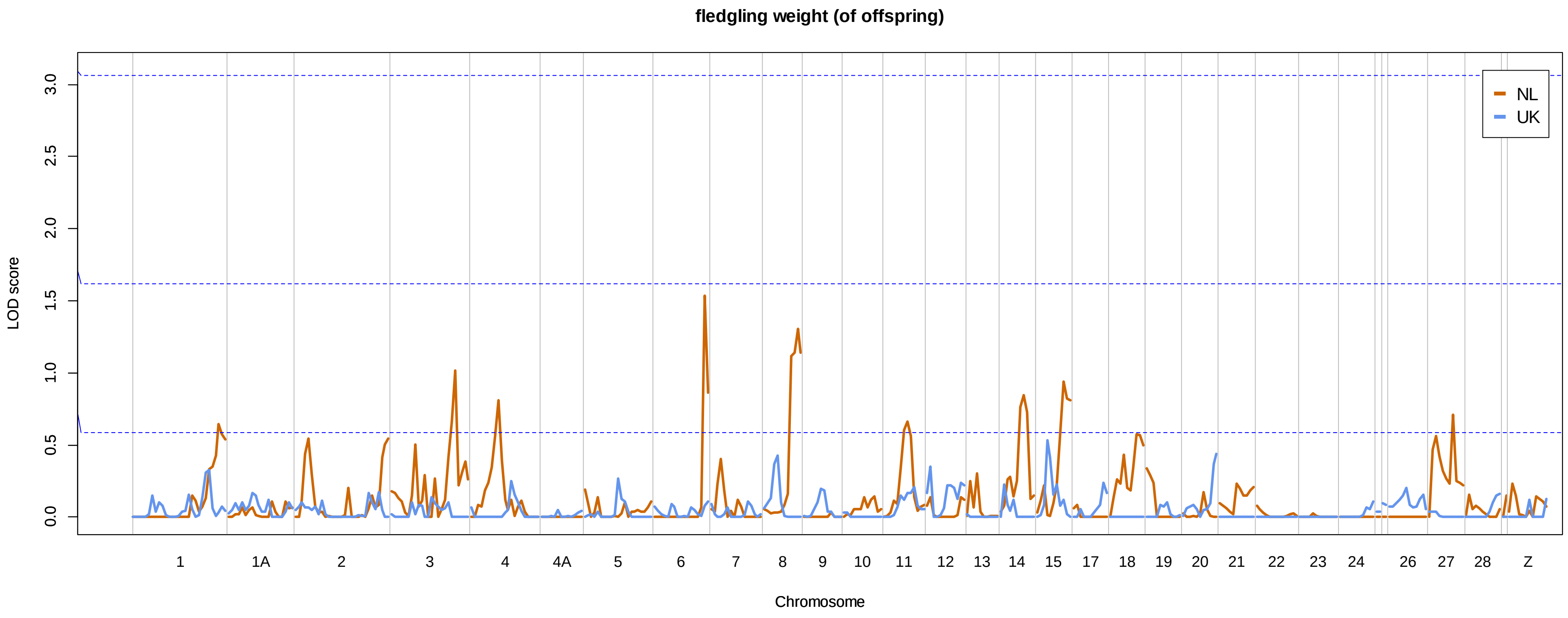

fledgling weight (of offspring)
LOD score
0.0
0.5
1.0
1.5
2.0
2.5
3.0
NL
UK
1
1A
2
3
4
4A
5
6
7
8
9
10
11
12
13
14
15
17
18
19
20
21
22
23
24
26
27
28
Z
Chromosome

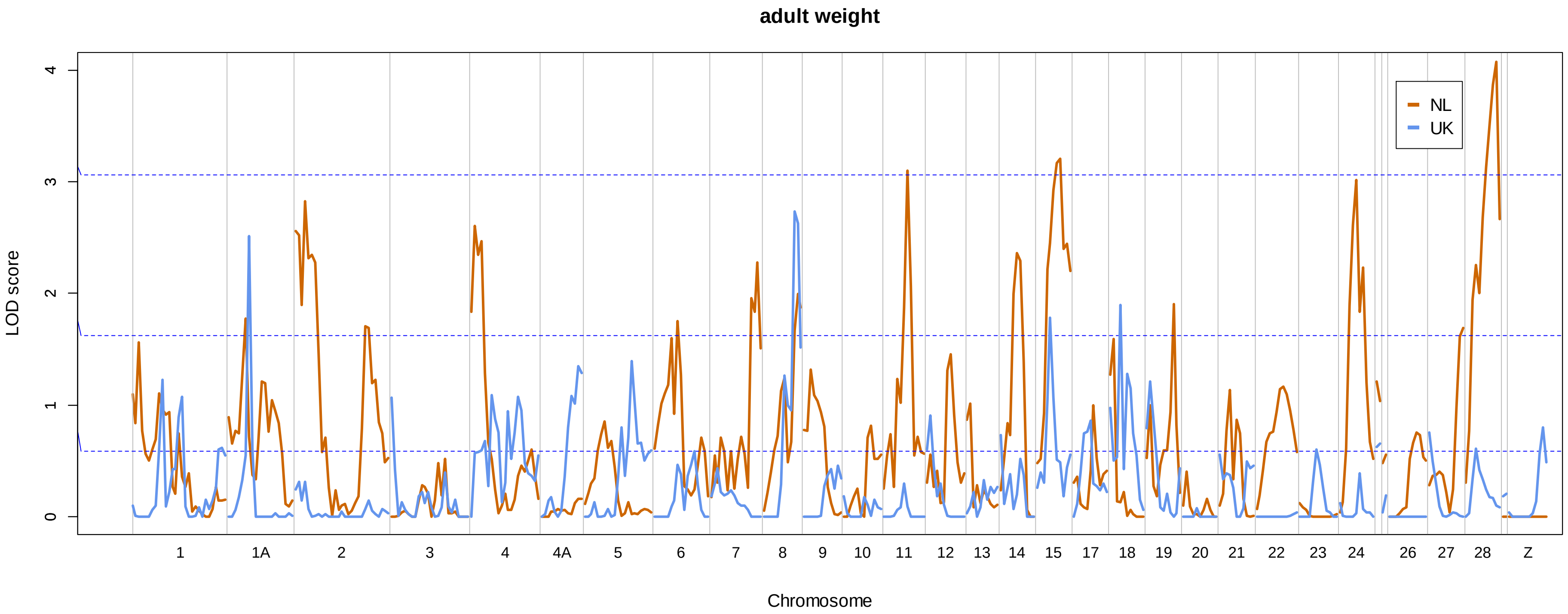
adult weight
LOD score
0
1
2
3
4
NL
UK
1
1A
2
3
4
4A
5
6
7
8
9
10
11
12
13
14
15
17
18
19
20
21
22
23
24
26
27
28
Z
Chromosome

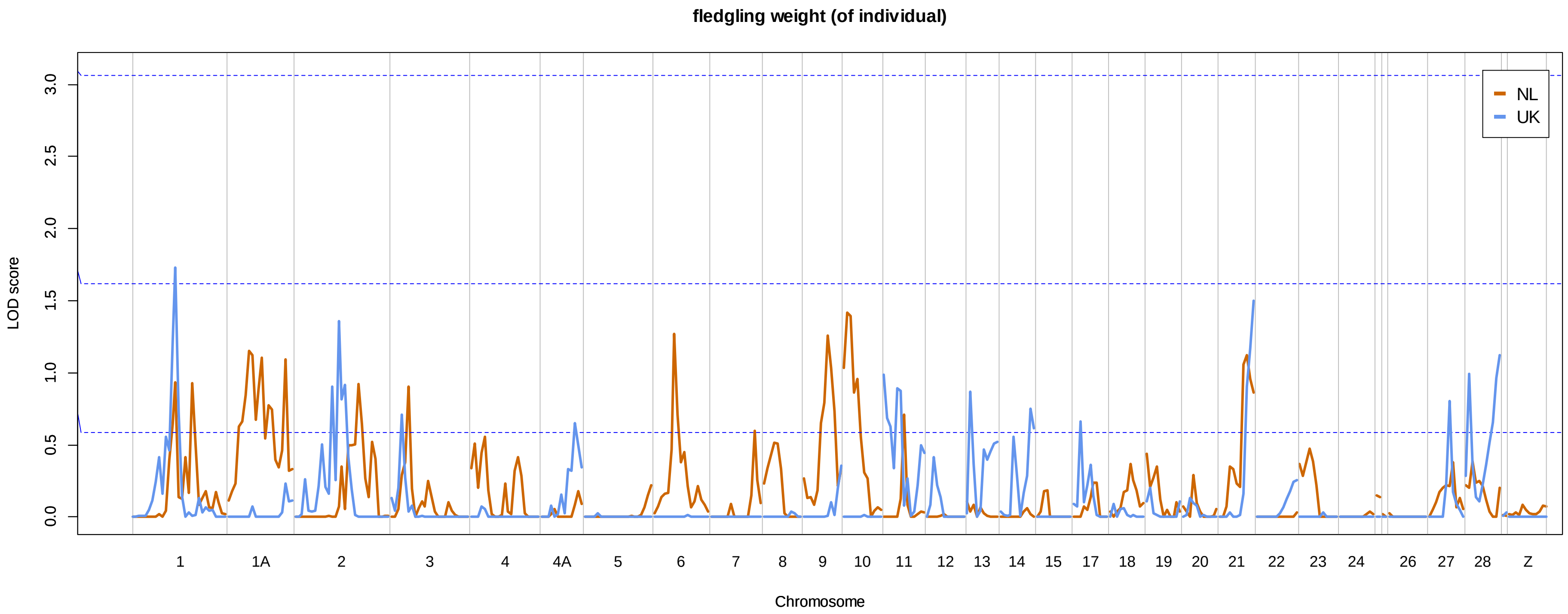

fledgling weight (of individual)
LOD score
0.0
0.5
1.0
1.5
2.0
2.5
3.0
NL
UK
1
1A
2
3
4
4A
5
6
7
8
9
10
11
12
13
14
15
17
18
19
20
21
22
23
24
26
27
28
Z
Chromosome

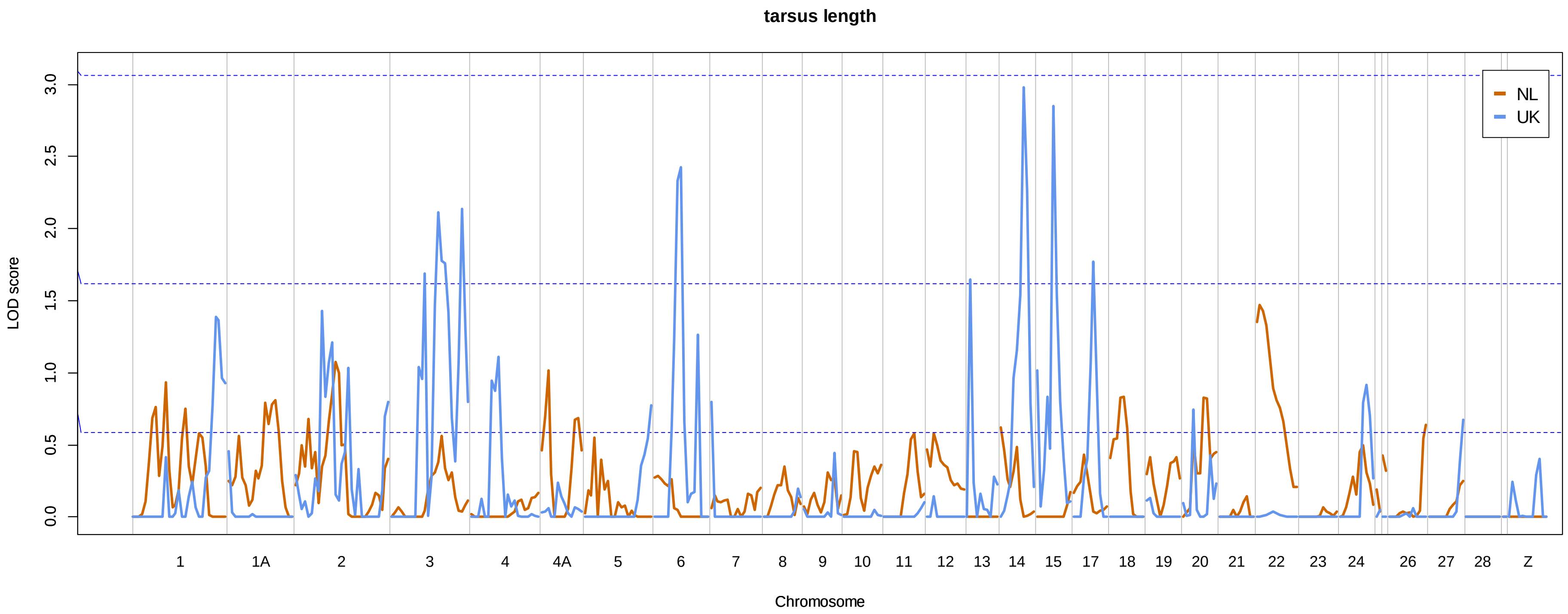

tarsus length
LOD score
0.0
0.5
1.0
1.5
2.0
2.5
3.0
NL
UK
1
1A
2
3
4
4A
5
6
7
8
9
10
11
12
13
14
15
17
18
19
20
21
22
23
24
26
27
28
Z
Chromosome

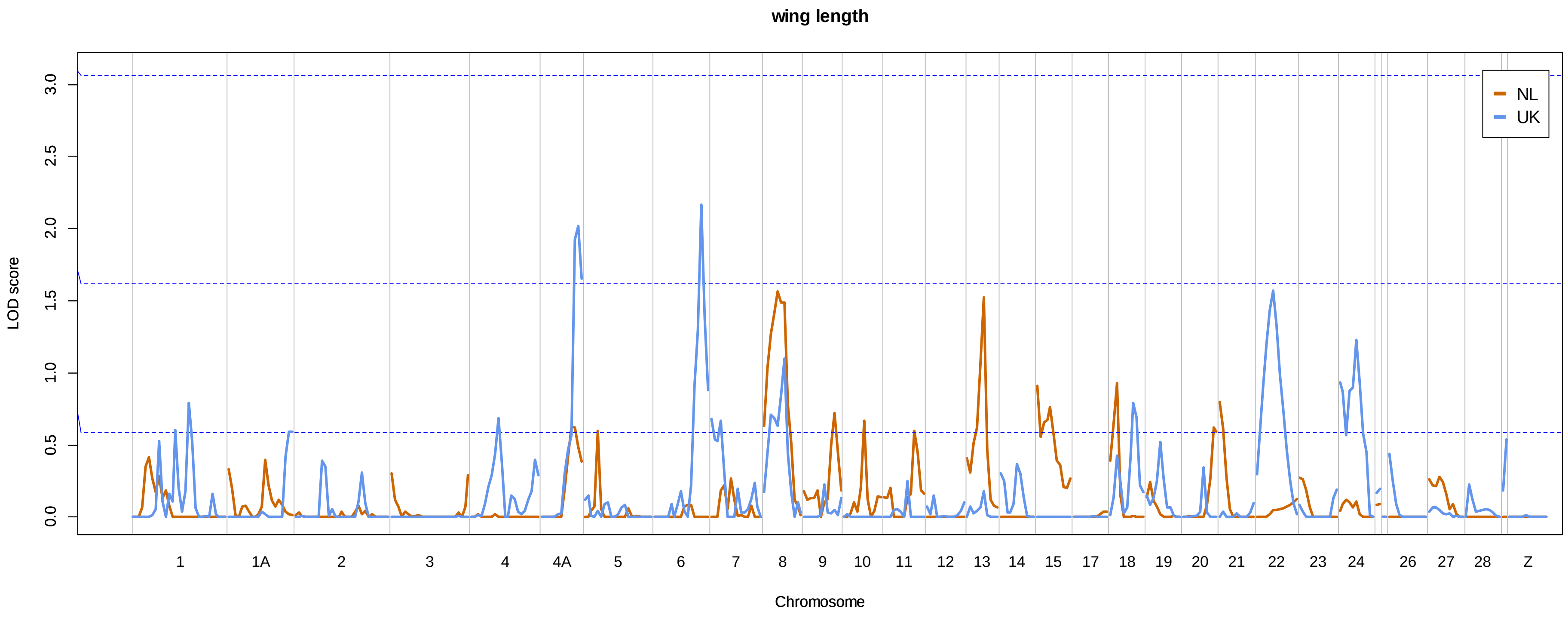
wing length
LOD score
0.0
0.5
1.0
1.5
2.0
2.5
3.0
NL
UK
1
1A
2
3
4
4A
5
6
7
8
9
10
11
12
13
14
15
17
18
19
20
21
22
23
24
26
27
28
Z
Chromosome

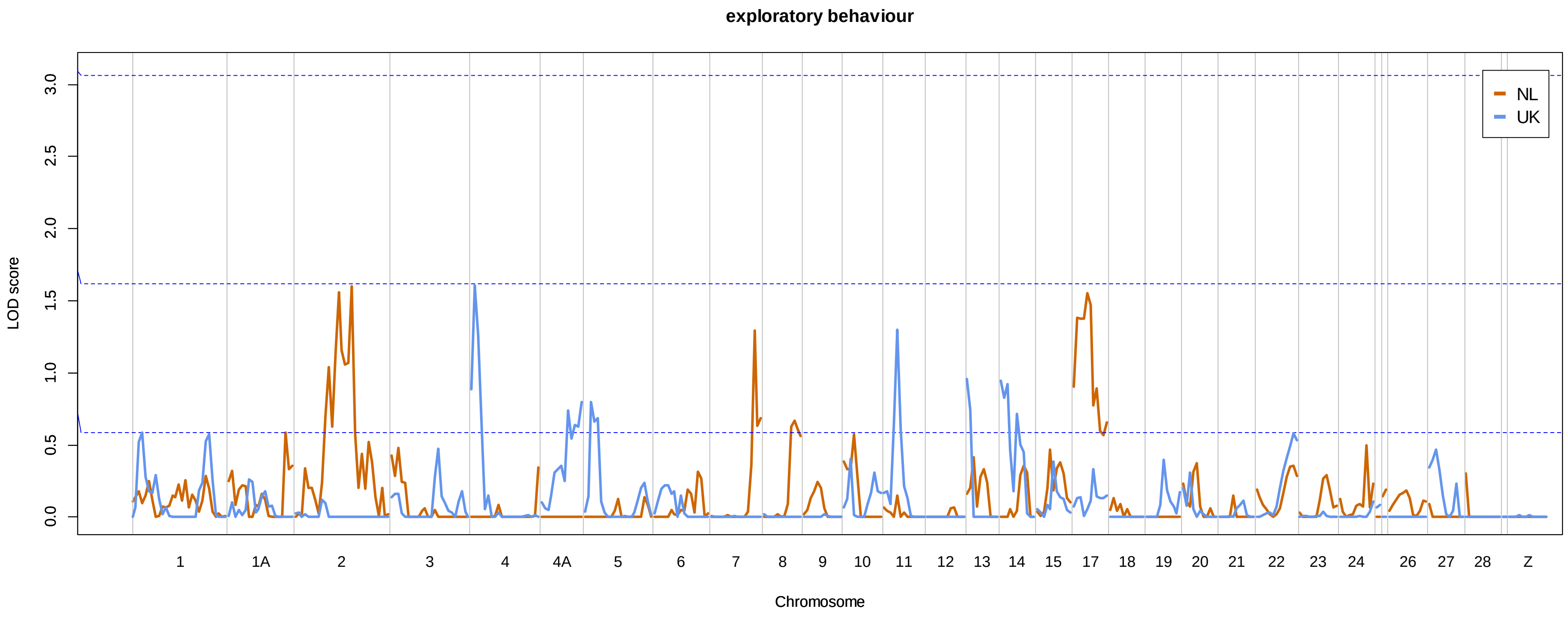
exploratory behaviour
LOD score
0.0
0.5
1.0
1.5
2.0
2.5
3.0
NL
UK
1
1A
2
3
4
4A
5
6
7
8
9
10
11
12
13
14
15
17
18
19
20
21
22
23
24
26
27
28
Z
Chromosome

Supporting Figure 3

Figures S4a-S4g: Null distribution of correlations between QTL LOD scores between the UK and NL for each trait. The observed value for the correlations are indicated by an arrow on each plot. Distributions were generated using the permutation approach of Keightley & Knott (1999), see Supporting Information 3 for more details.

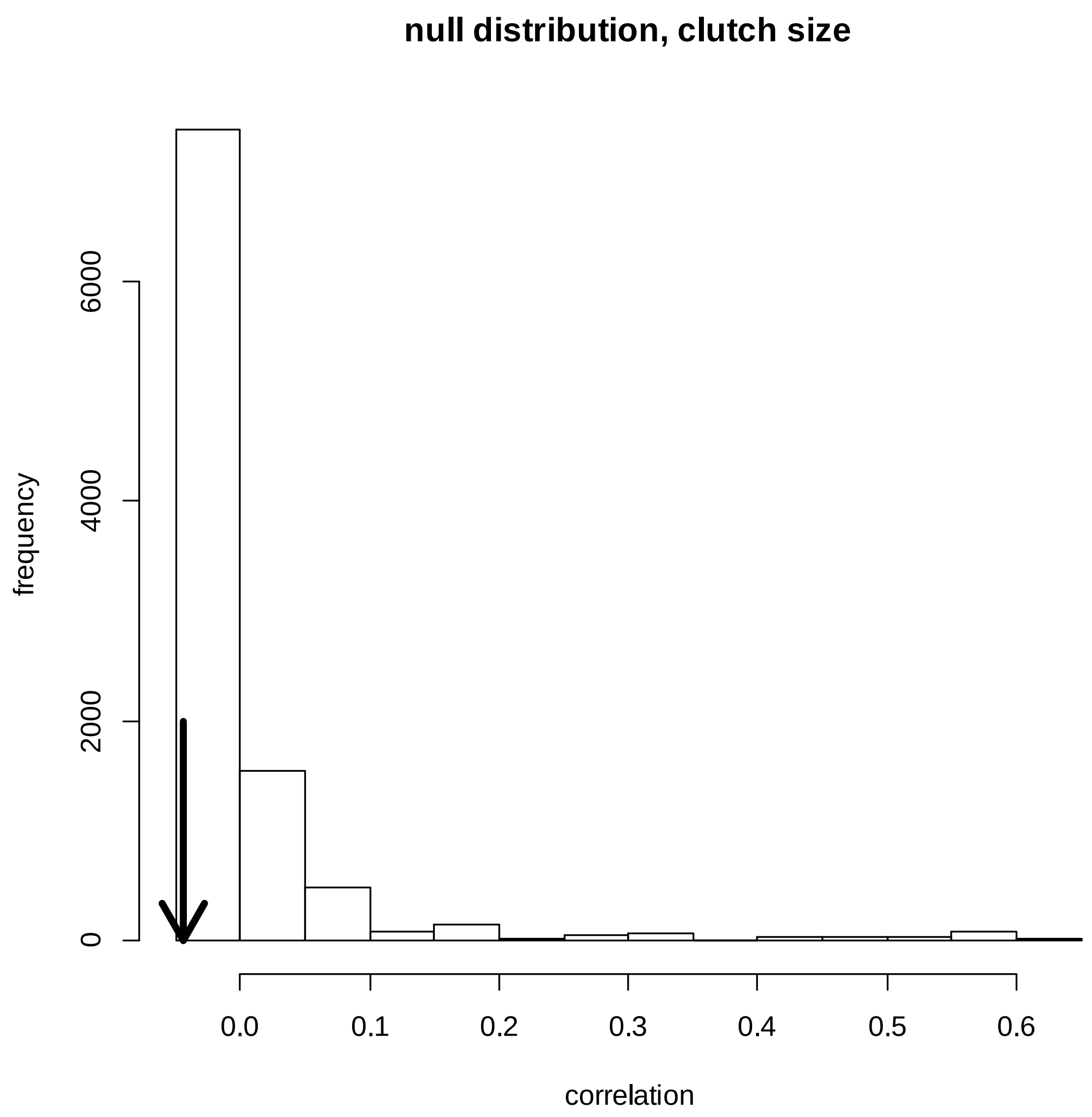

null distribution, clutch size
frequency
0
2000
4000
6000
0.0
0.1
0.2
0.3
0.4
0.5
0.6
correlation

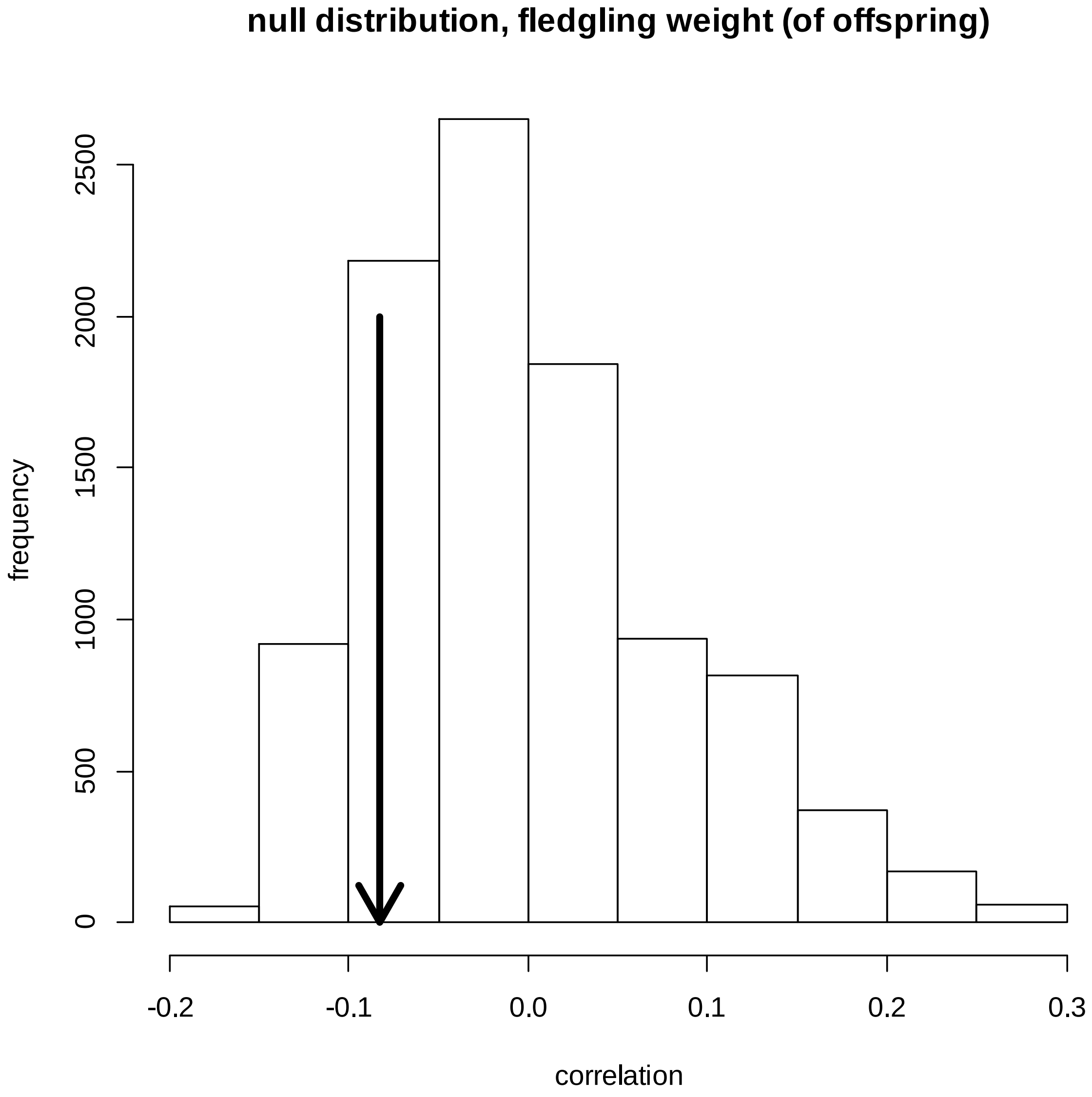
null distribution, fledgling weight (of offspring)
frequency
0
500
1000
1500
2000
2500
-0.2
-0.1
0.0
0.1
0.2
0.3
correlation

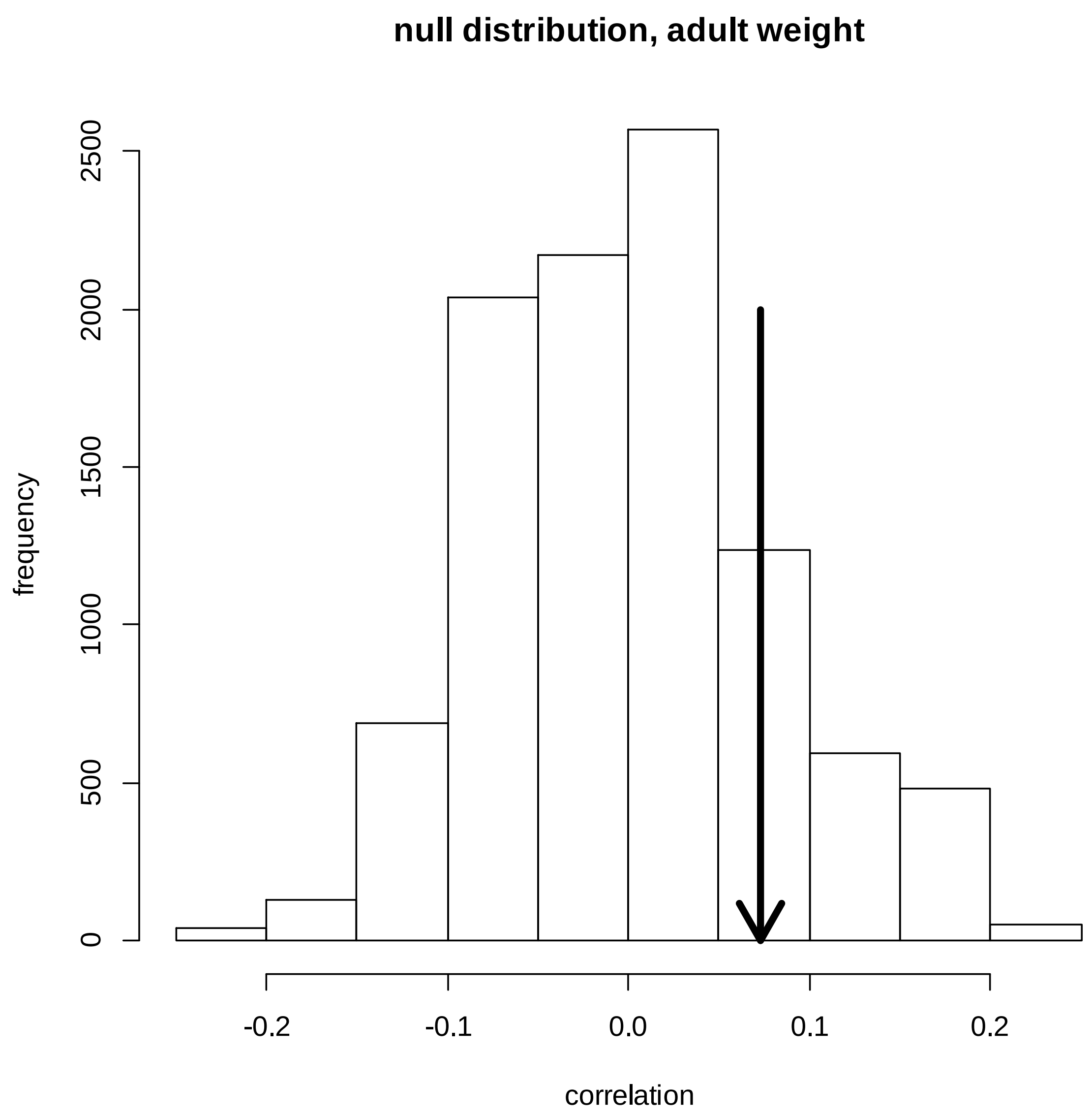
null distribution, adult weight
frequency
0
500
1000
1500
2000
2500
-0.2
-0.1
0.0
0.1
0.2
correlation

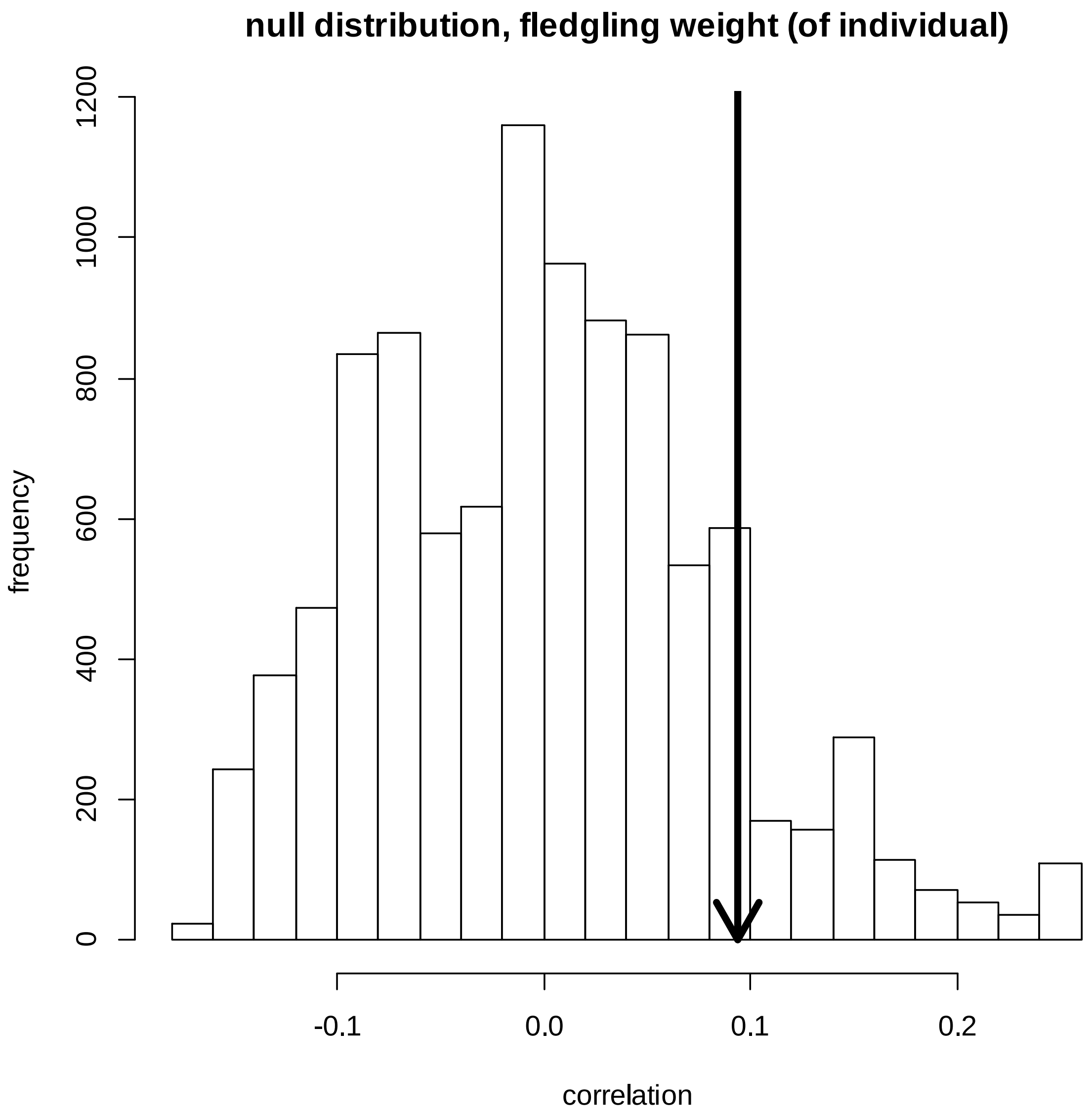
null distribution, fledgling weight (of individual)
frequency
0
200
400
600
800
1000
1200
-0.1
0.0
0.1
0.2
correlation

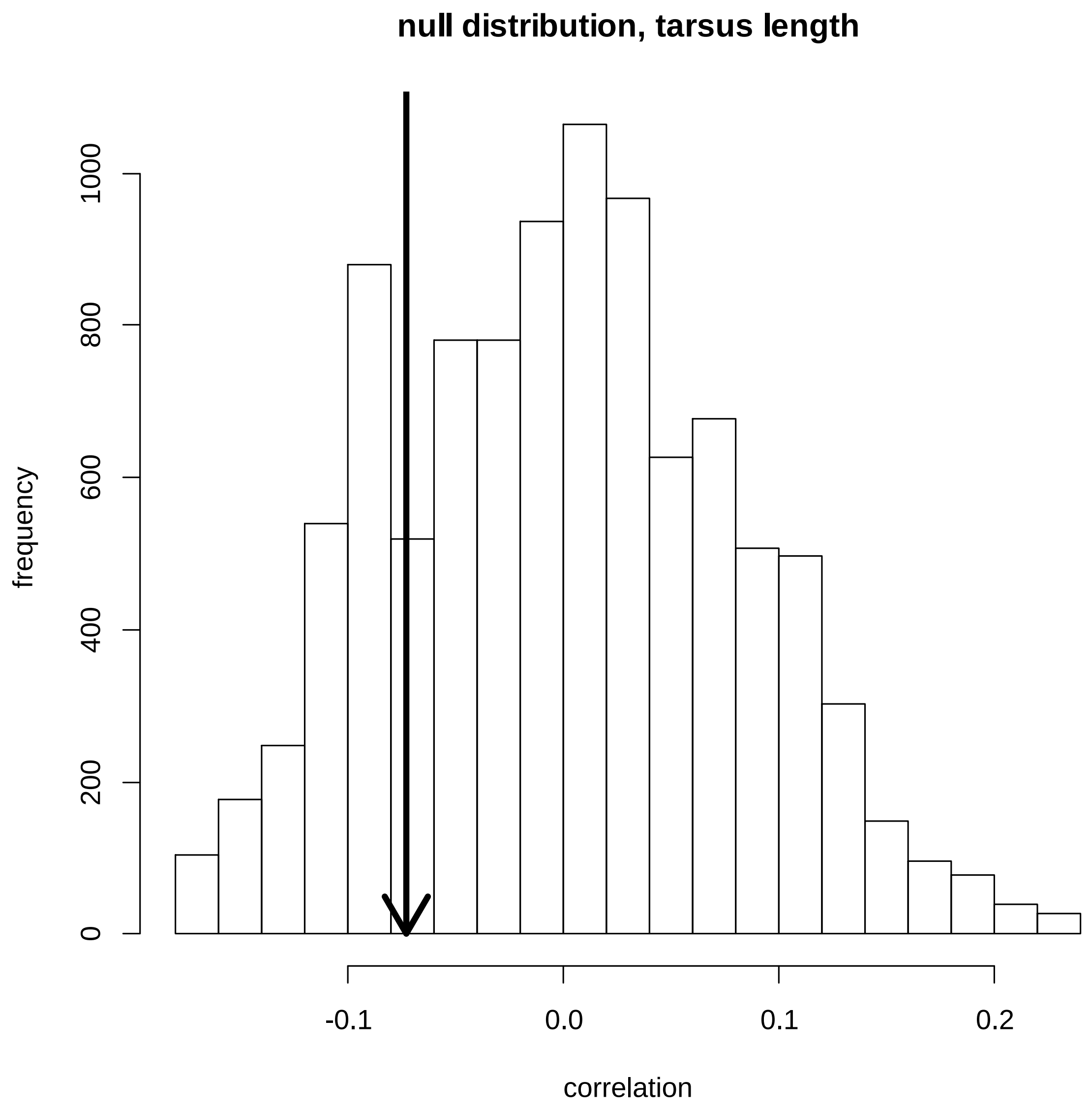
null distribution, tarsus length
frequency
0
200
400
600
800
1000
-0.1
0.0
0.1
0.2
correlation

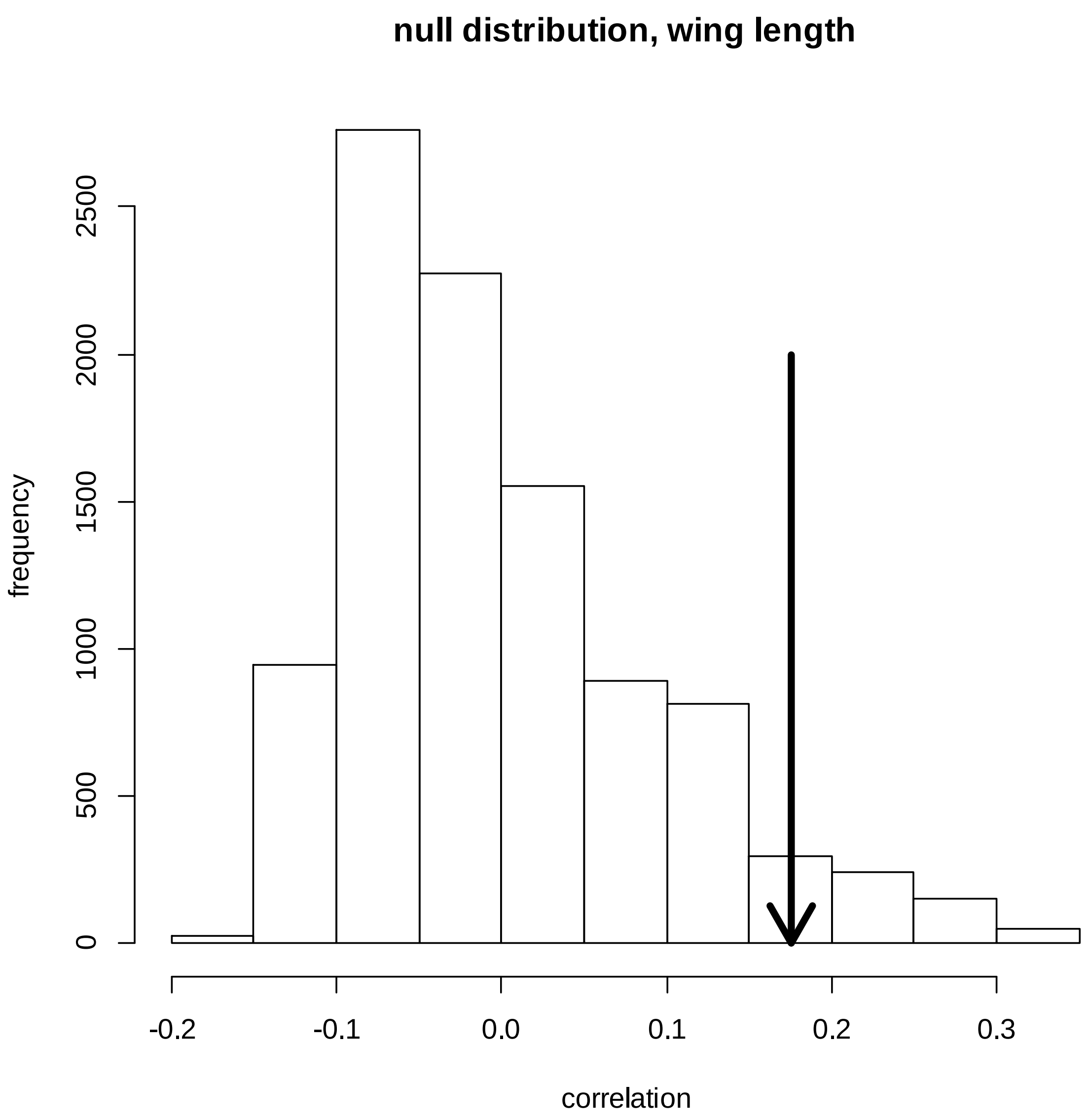
null distribution, wing length
frequency
0
500
1000
1500
2000
2500
-0.2
-0.1
0.0
0.1
0.2
0.3
correlation

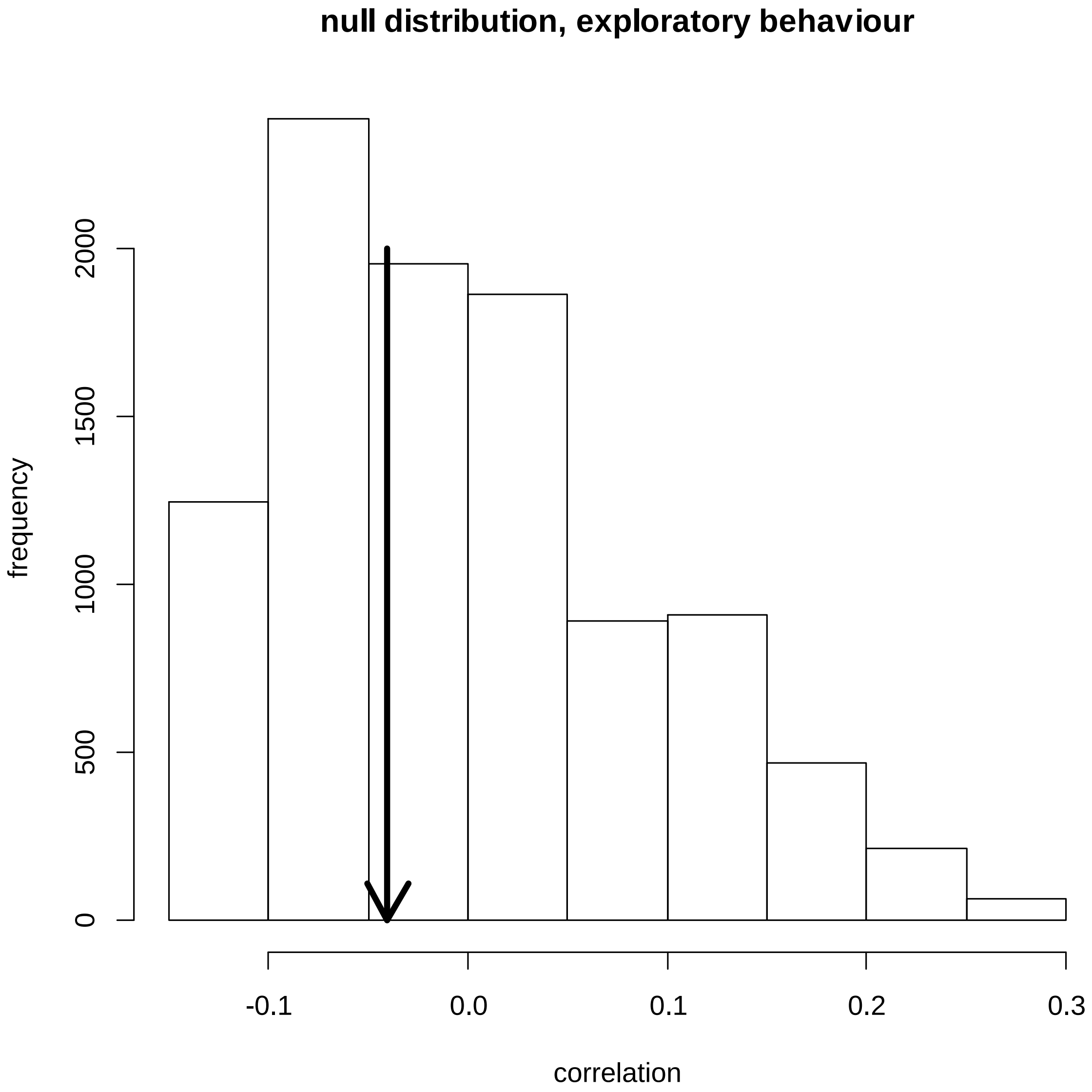
null distribution, exploratory behaviour
frequency
0
500
1000
1500
2000
-0.1
0.0
0.1
0.2
0.3
correlation

Supporting Figure 4

Figures S5a-S5h: GWAS plots for the quantitative traits in the NL and UK populations, and for both populations combined. Dashed blue lines show nominal (p = 0.050, -log10(p) = 1.301) and significant (p = 9.0 x 10-6, -log10(p) = 5.046) scores. Chromosome labels are shown beneath the plots; chromosomes 25A and 25B (plotted after chromosome 24) and LGE22 (after 28) are not labelled.

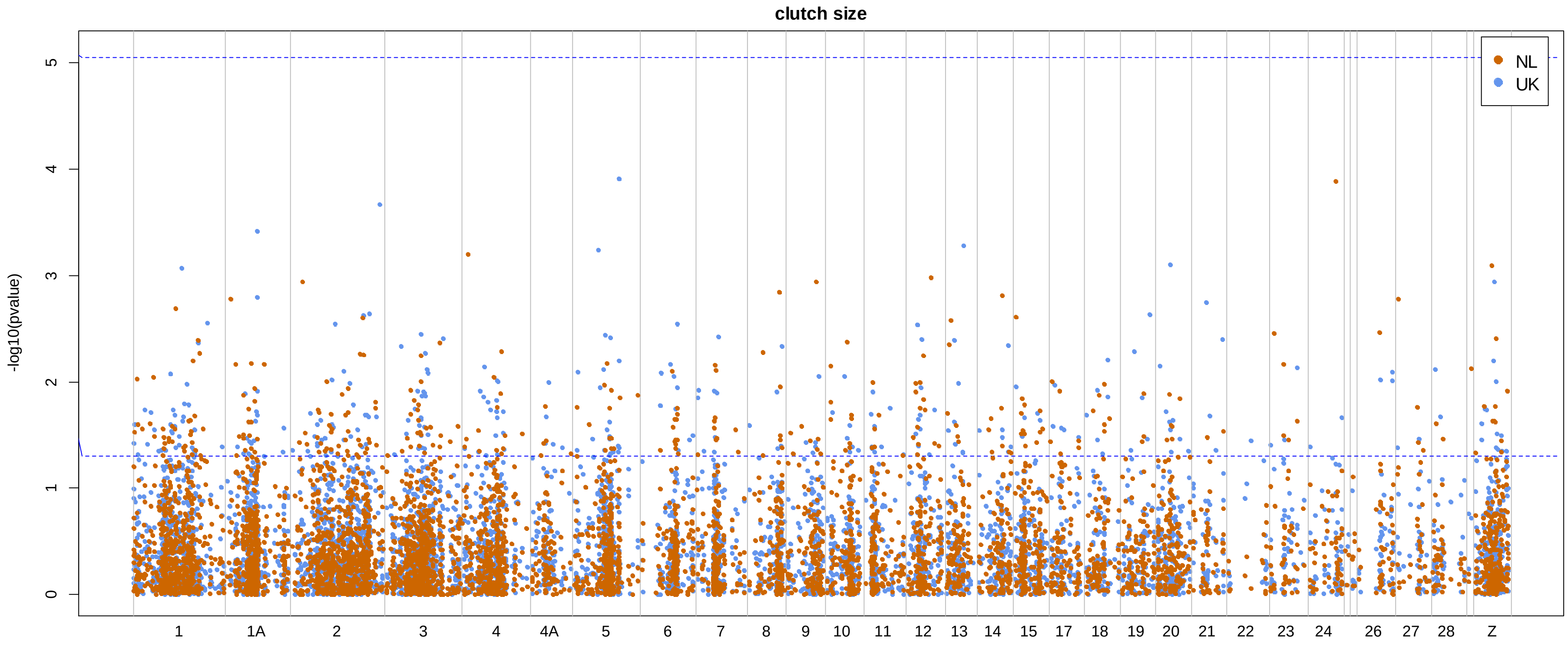
clutch size
-log10(pvalue)
NL
UK
0
1
2
3
4
5
1
1A
2
3
4
4A
5
6
7
8
9
10
11
12
13
14
15
17
18
19
20
21
22
23
24
26
27
28
Z

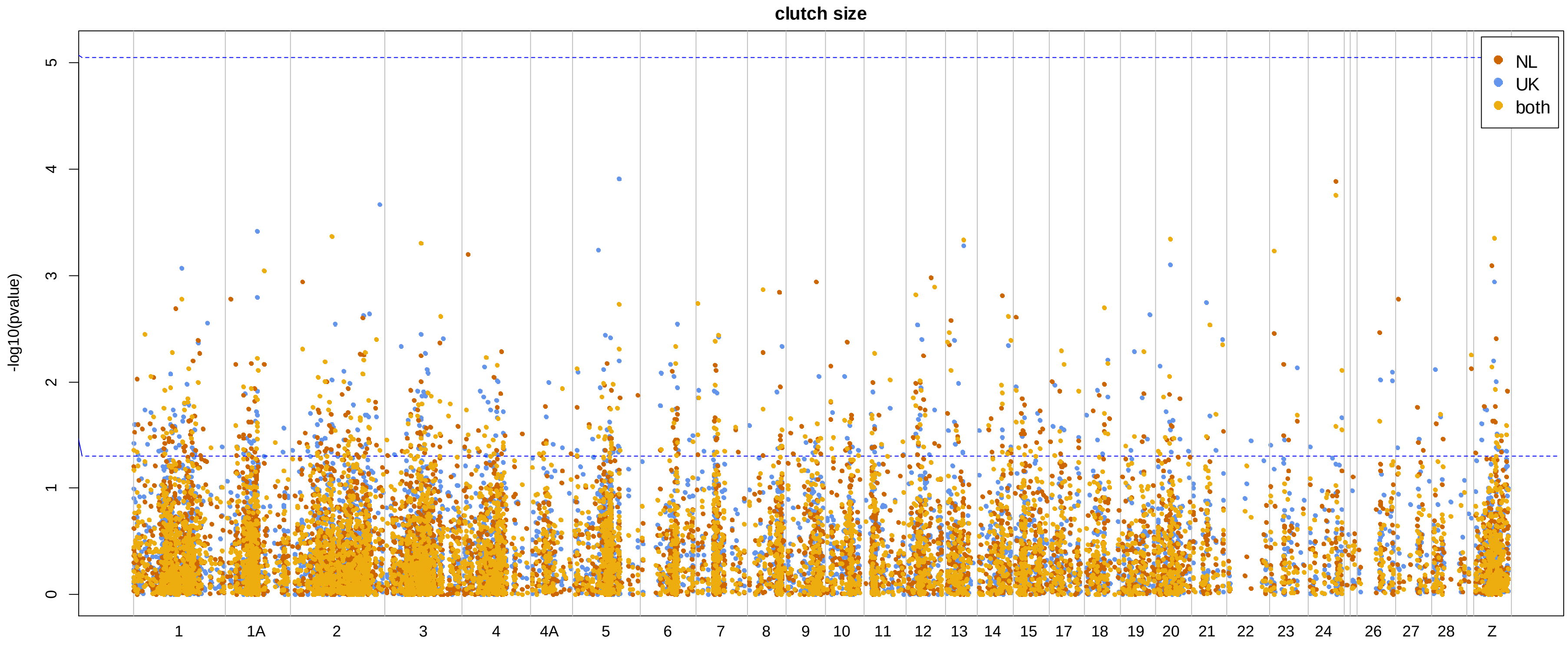
clutch size
-log10(pvalue)
0
1
2
3
4
5
NL
UK
both
1
1A
2
3
4
4A
5
6
7
8
9
10
11
12
13
14
15
17
18
19
20
21
22
23
24
26
27
28
Z

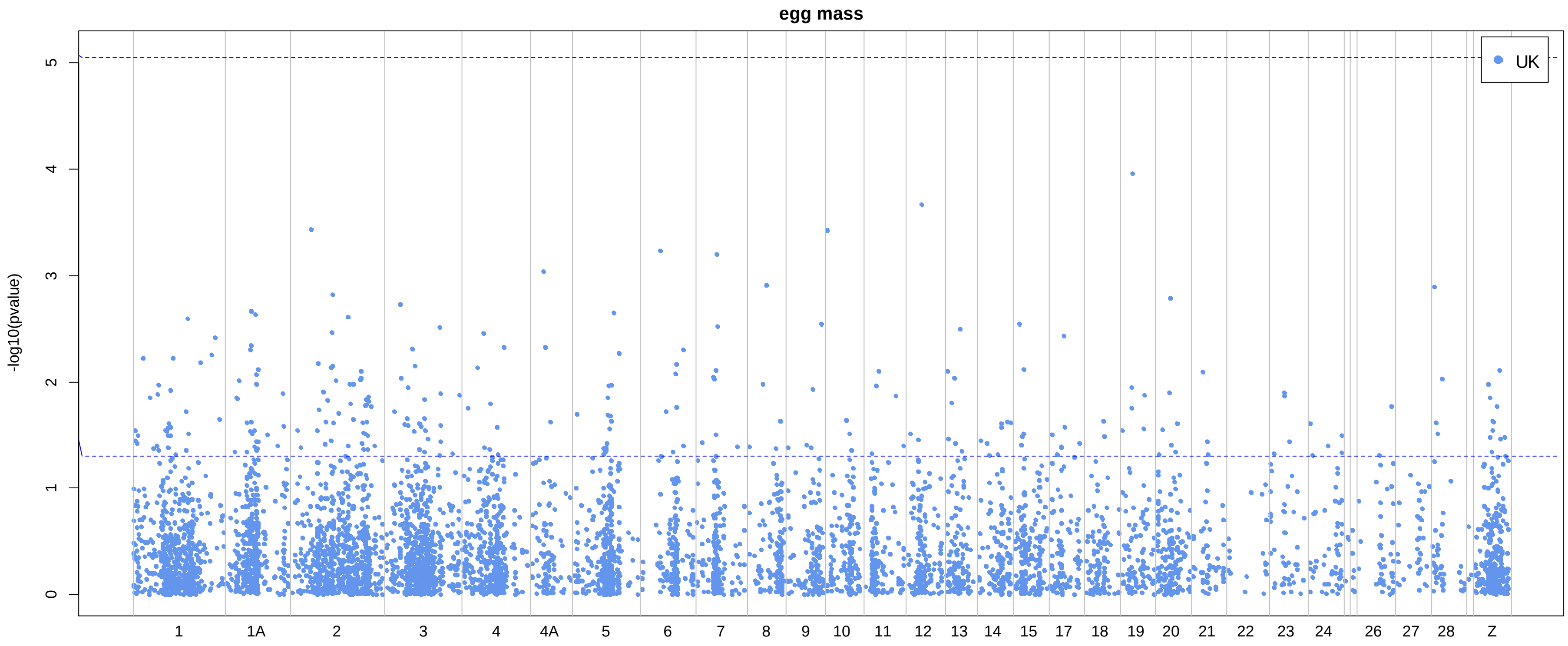
egg mass
-log10(pvalue)
UK
0
1
2
3
4
5
1
1A
2
3
4
4A
5
6
7
8
9
10
11
12
13
14
15
17
18
19
20
21
22
23
24
26
27
28
Z

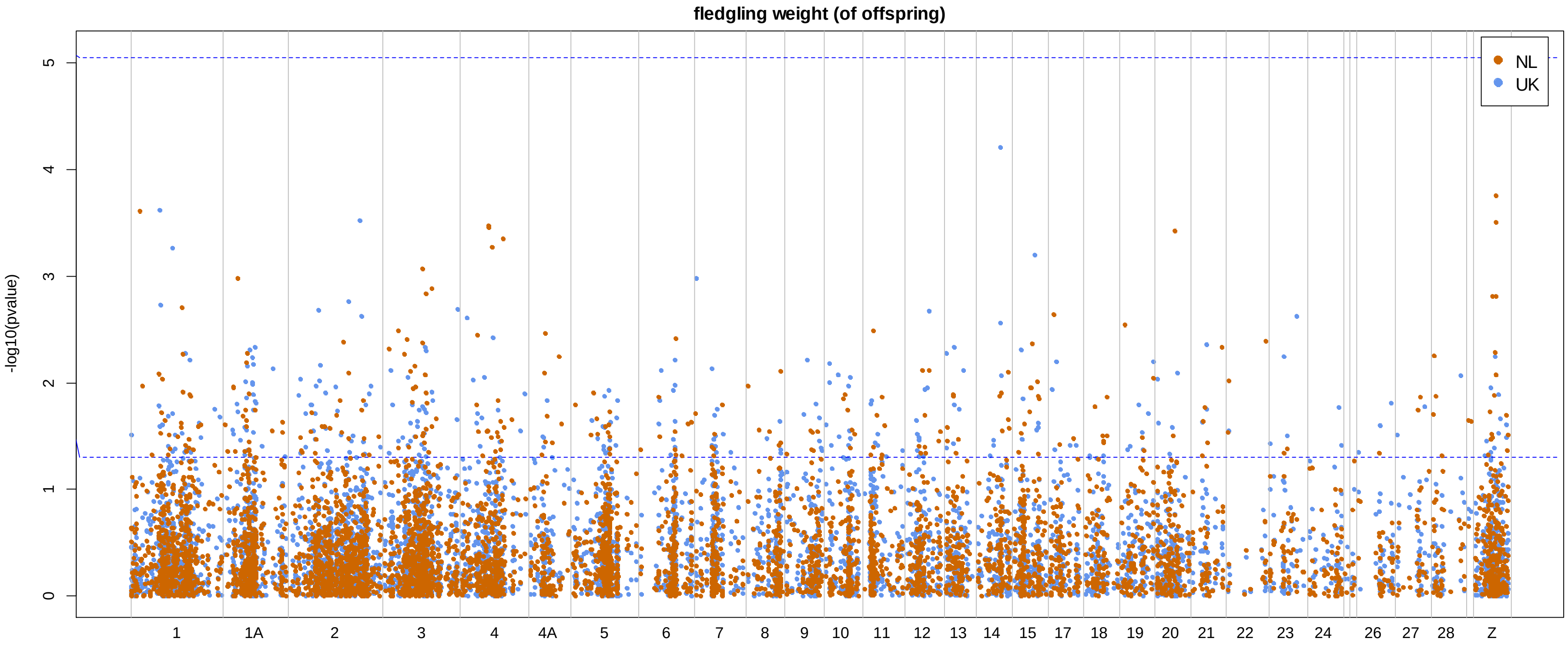

fledgling weight (of offspring)
-log10(pvalue)
0
1
2
3
4
5
NL
UK
1
1A
2
3
4
4A
5
6
7
8
9
10
11
12
13
14
15
17
18
19
20
21
22
23
24
26
27
28
Z

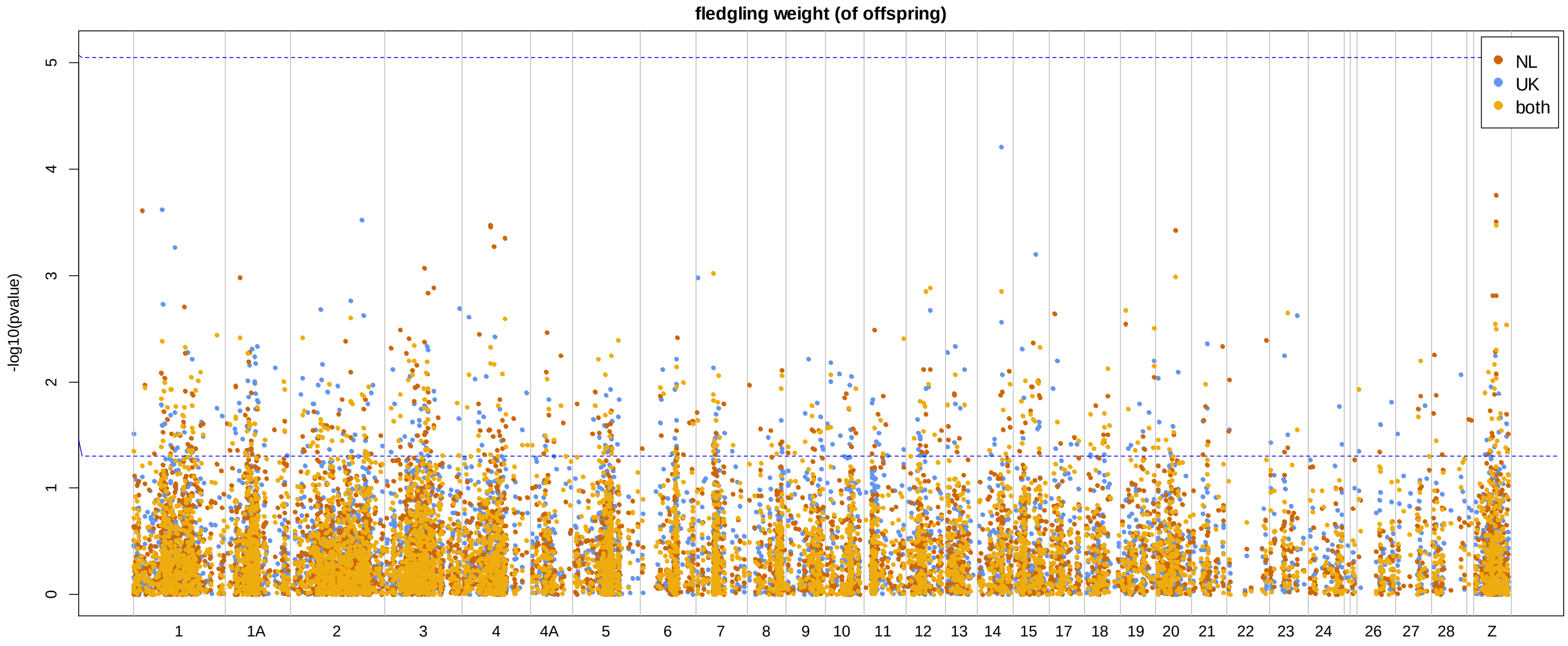

fledgling weight (of offspring)
-log10(pvalue)
0
1
2
3
4
5
NL
UK
both
1
1A
2
3
4
4A
5
6
7
8
9
10
11
12
13
14
15
17
18
19
20
21
22
23
24
26
27
28
Z

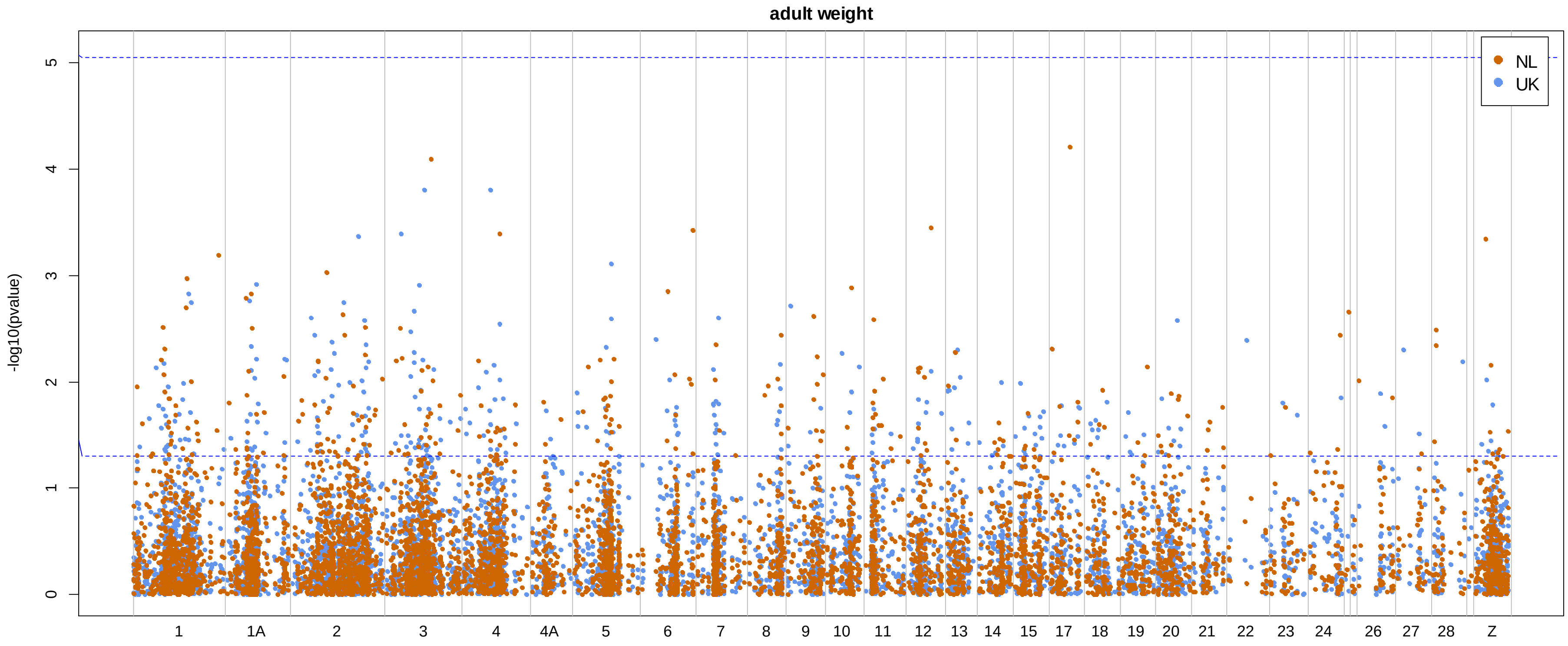

adult weight
NL
UK
-log10(pvalue)
0
1
2
3
4
5
1
1A
2
3
4
4A
5
6
7
8
9
10
11
12
13
14
15
17
18
19
20
21
22
23
24
26
27
28
Z

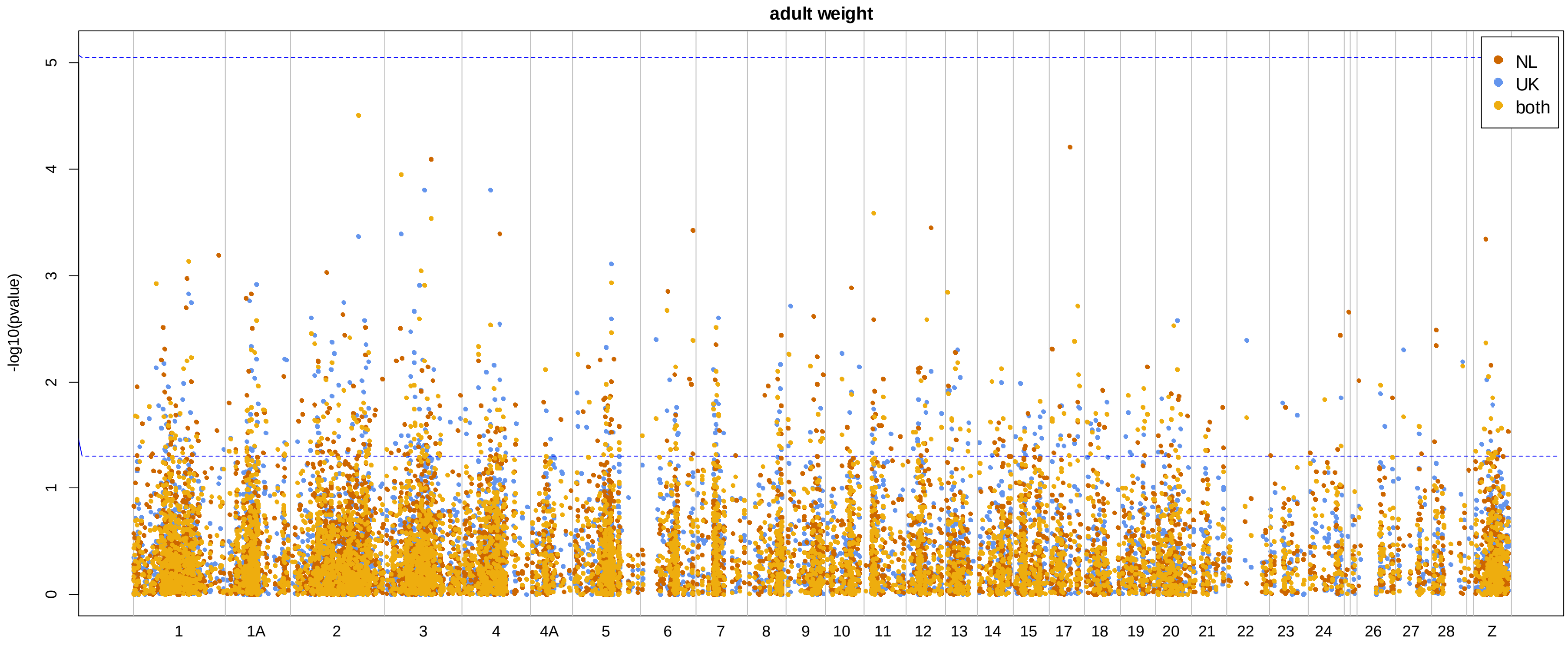
adult weight
-log10(pvalue)
NL
UK
both
0
1
2
3
4
5
1
1A
2
3
4
4A
5
6
7
8
9
10
11
12
13
14
15
17
18
19
20
21
22
23
24
26
27
28
Z

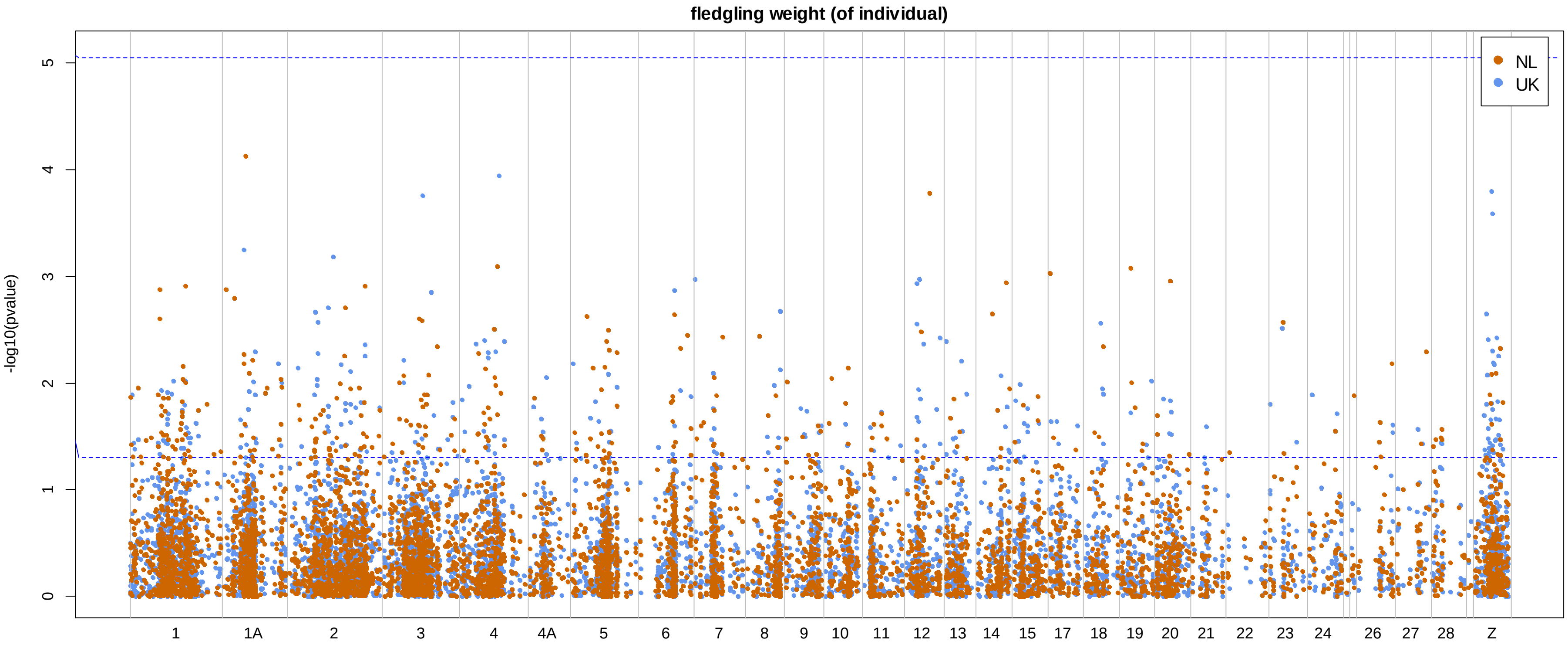

fledgling weight (of individual)
-log10(pvalue)
0
1
2
3
4
5
NL
UK
1
1A
2
3
4
4A
5
6
7
8
9
10
11
12
13
14
15
17
18
19
20
21
22
23
24
26
27
28
Z

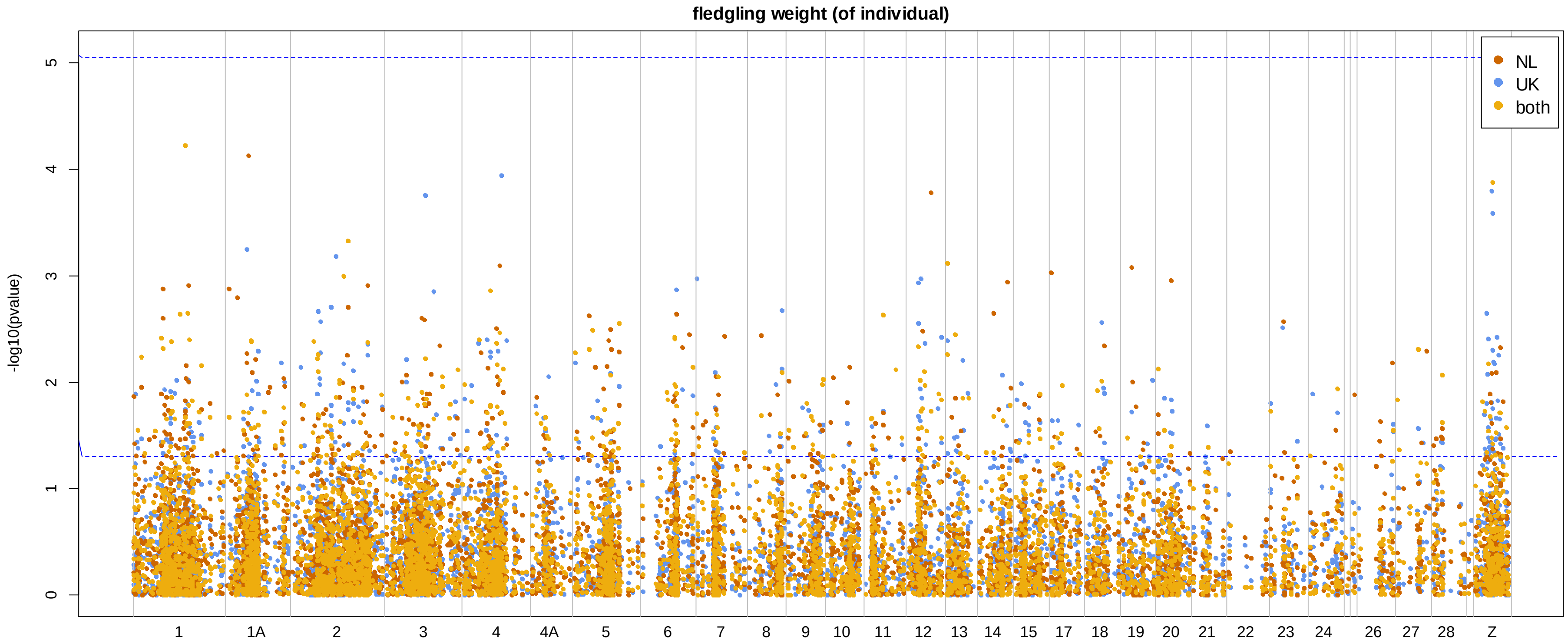
fledgling weight (of individual)
-log10(pvalue)
0
1
2
3
4
5
1
1A
2
3
4
4A
5
6
7
8
9
10
11
12
13
14
15
17
18
19
20
21
22
23
24
26
27
28
Z
NL
UK
both

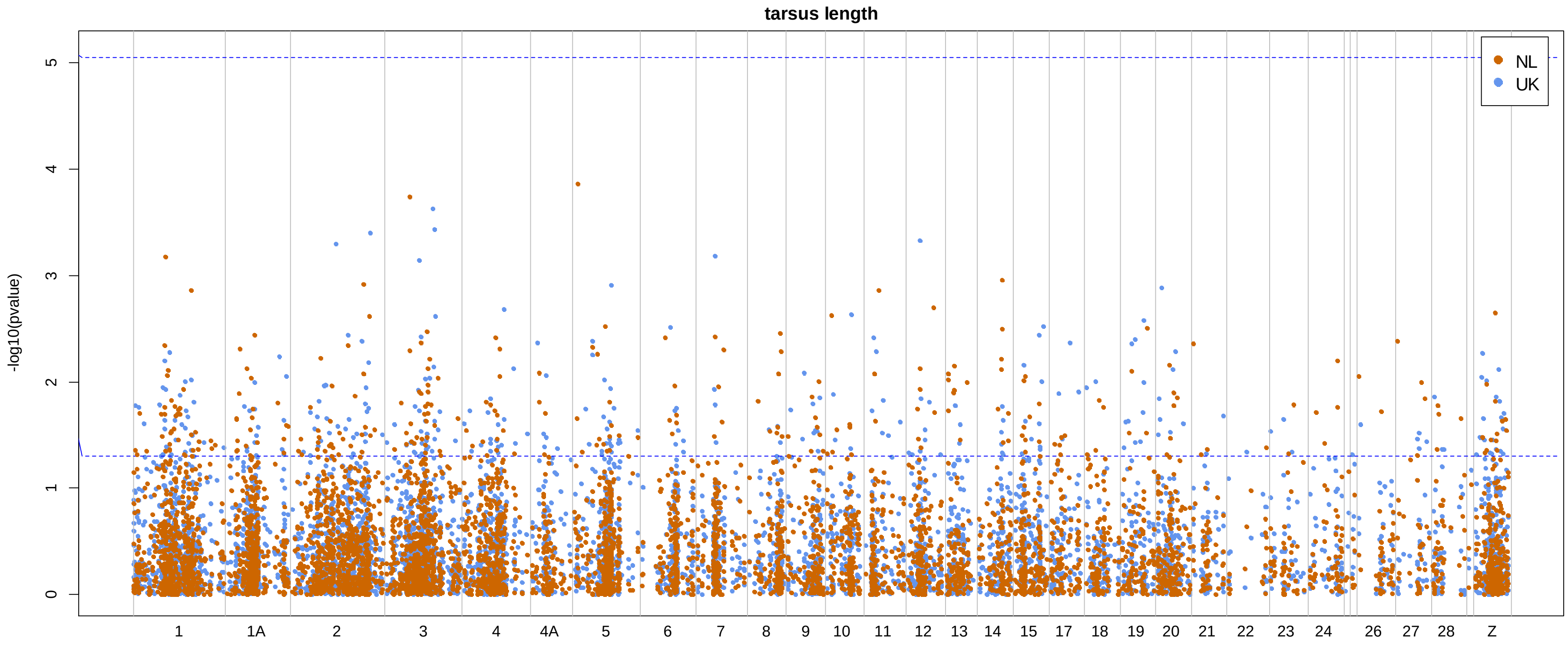

tarsus length
NL
UK
-log10(pvalue)
0
1
2
3
4
5
1
1A
2
3
4
4A
5
6
7
8
9
10
11
12
13
14
15
17
18
19
20
21
22
23
24
26
27
28
Z

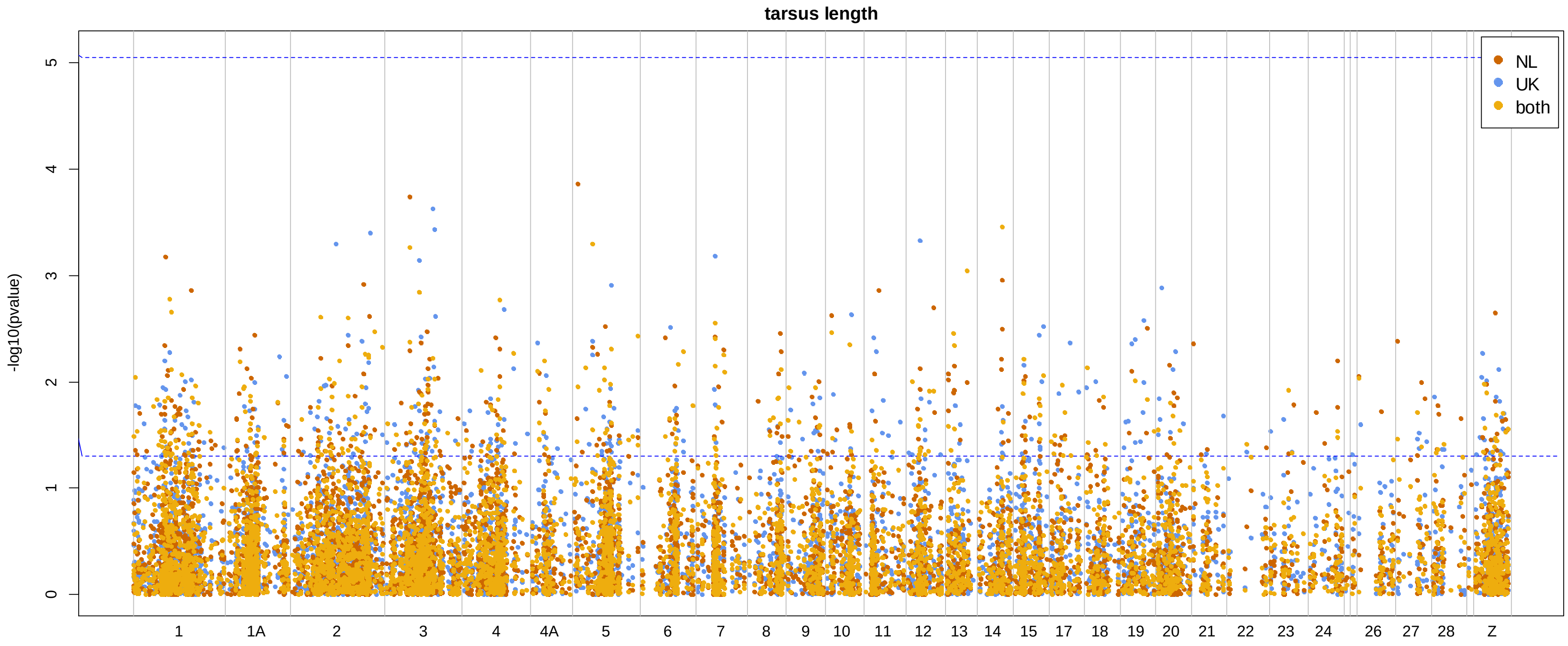

tarsus length
-log10(pvalue)
0
1
2
3
4
5
NL
UK
both
1
1A
2
3
4
4A
5
6
7
8
9
10
11
12
13
14
15
17
18
19
20
21
22
23
24
26
27
28
Z

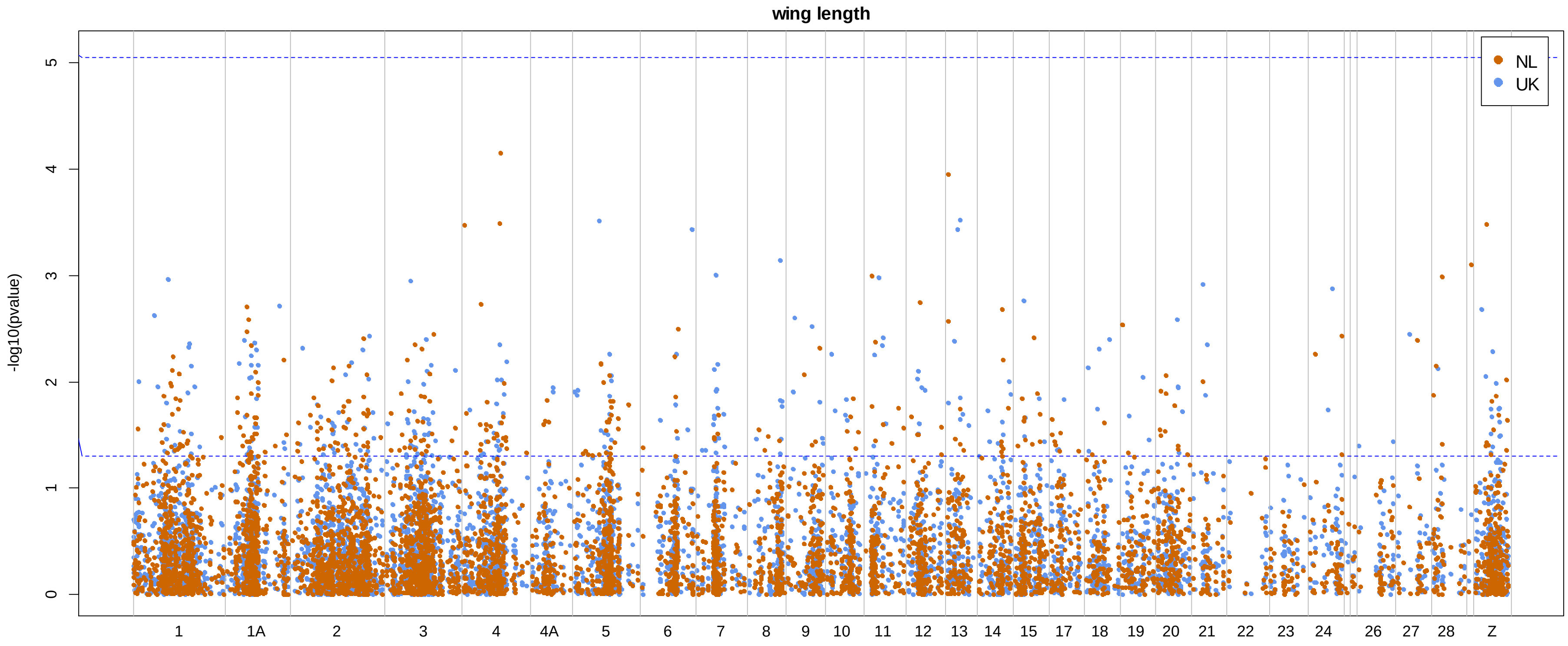

wing length
NL
UK
-log10(pvalue)
0
1
2
3
4
5
1
1A
2
3
4
4A
5
6
7
8
9
10
11
12
13
14
15
17
18
19
20
21
22
23
24
26
27
28
Z

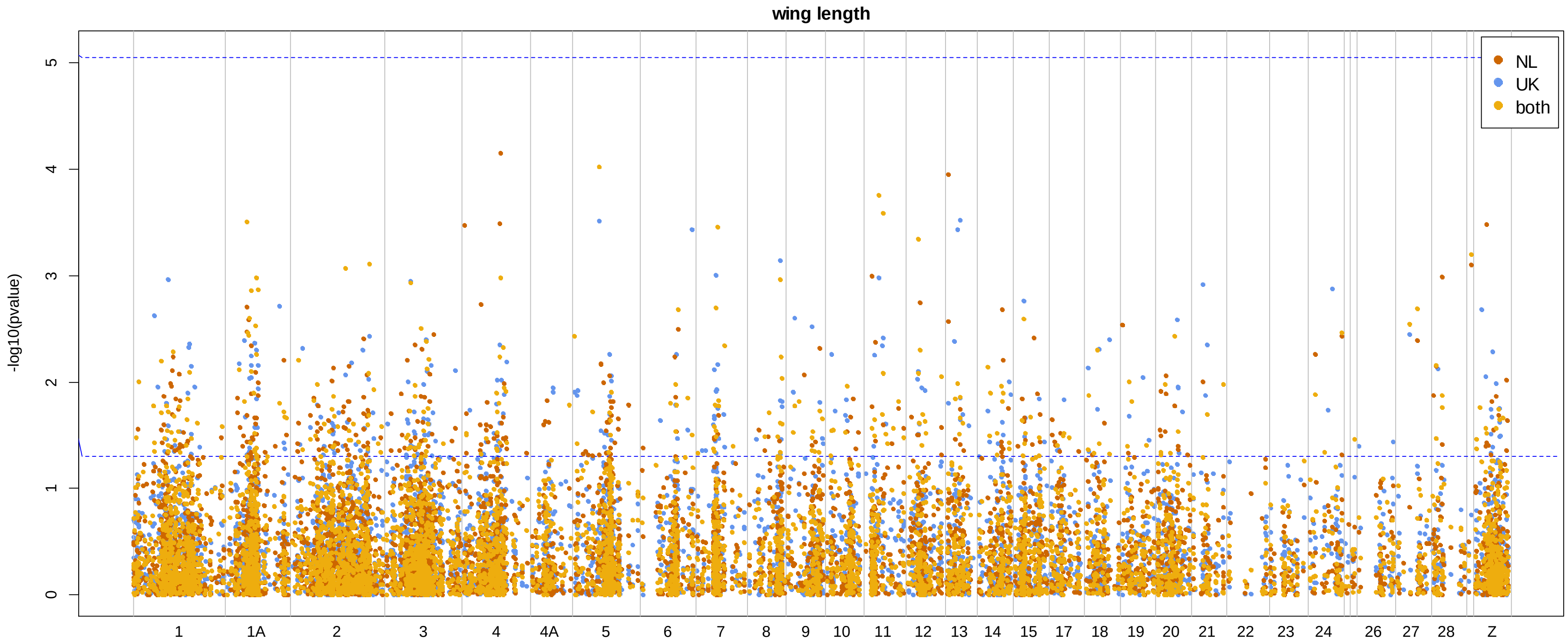

wing length
-log10(pvalue)
0
1
2
3
4
5
NL
UK
both
1
1A
2
3
4
4A
5
6
7
8
9
10
11
12
13
14
15
17
18
19
20
21
22
23
24
26
27
28
Z

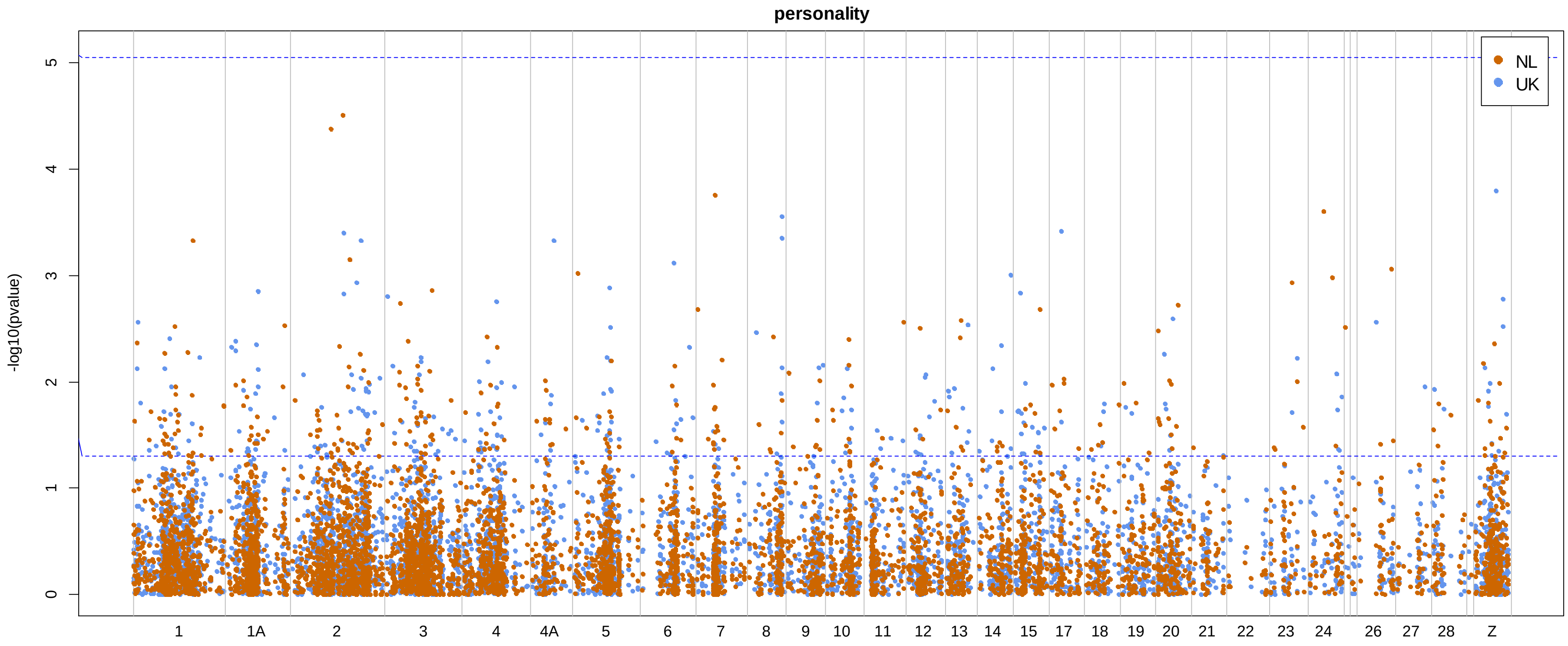
personality
-log10(pvalue)
0
1
2
3
4
5
NL
UK
1
1A
2
3
4
4A
5
6
7
8
9
10
11
12
13
14
15
17
18
19
20
21
22
23
24
26
27
28
Z

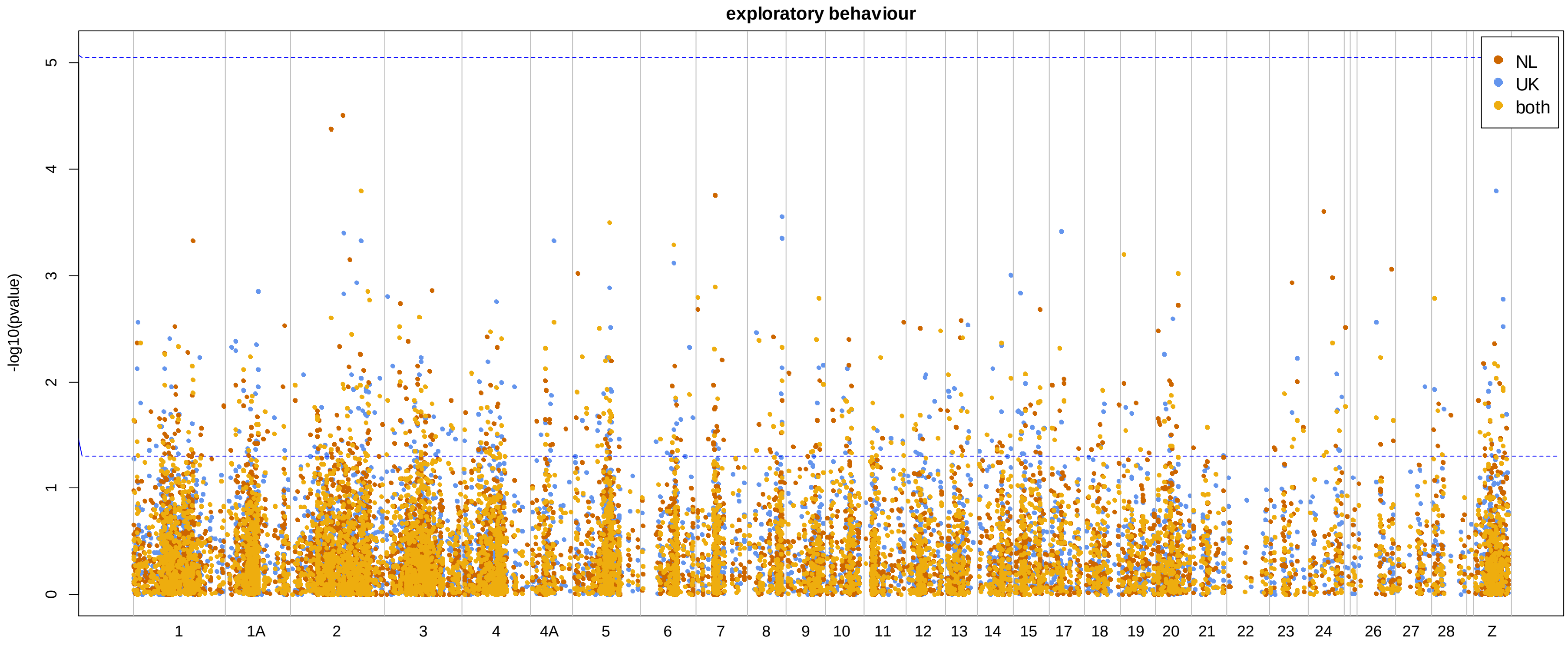

exploratory behaviour
-log10(pvalue)
0
1
2
3
4
5
NL
UK
both
1
1A
2
3
4
4A
5
6
7
8
9
10
11
12
13
14
15
17
18
19
20
21
22
23
24
26
27
28
Z

Supporting Figure 5

Plots of estimated effect size for each SNP in the UK and NL populations.

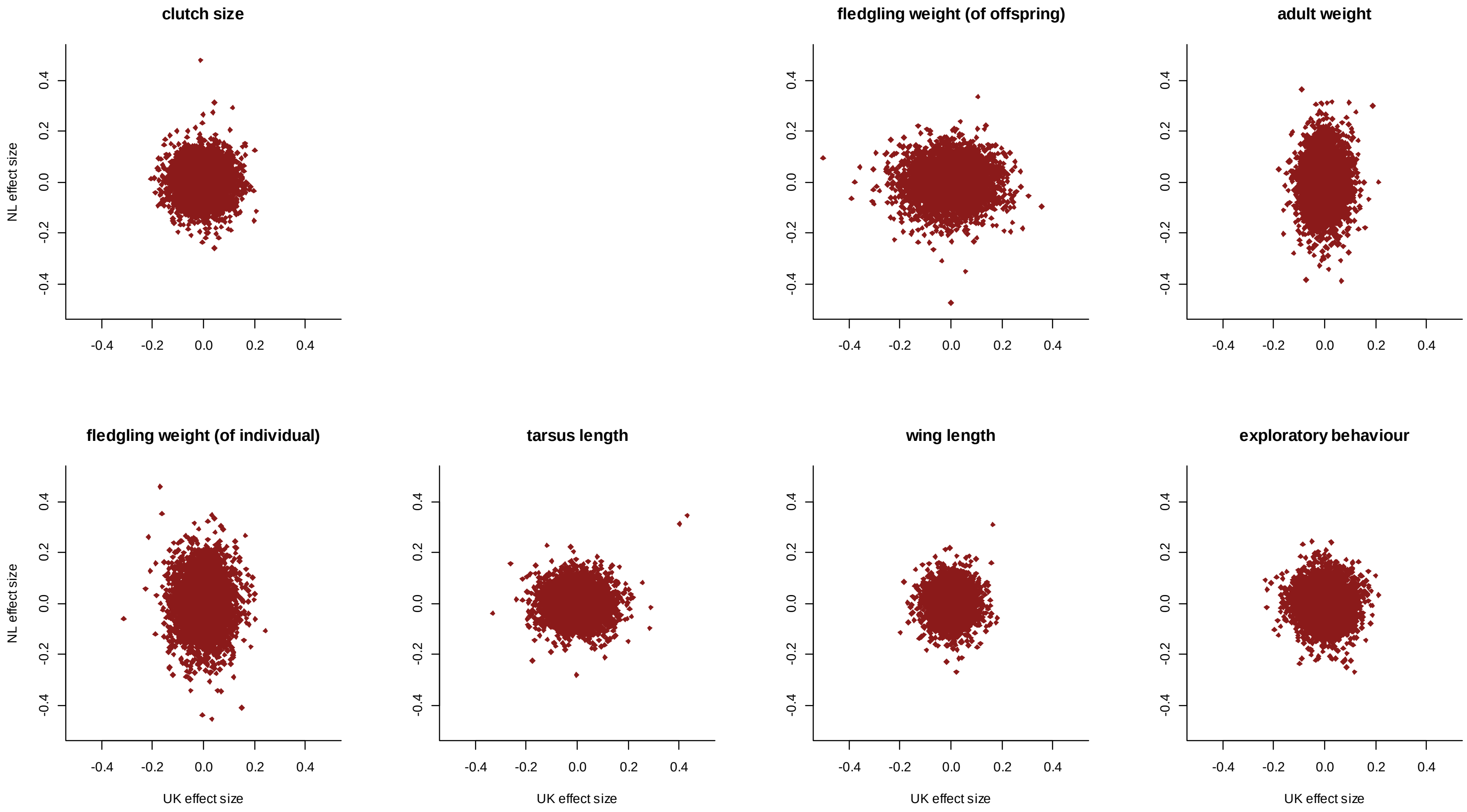

Supplement: Supplementary file 1 — Fig. S1 Relationship between variance explained by each chromosome for maternal, morphological and personality traits in the NL and UK populations. Fig. S2 (a–h) QTL scans for the quantitative traits in the NL (orange) and UK (blue) populations. Fig. S3 (a–g) Null distribution of correlations between QTL LOD scores between the UK and NL for each trait. Fig. S4 (a–h) GWAS plots for the quantitative traits in the NL and UK populations. Fig. S5 Plots of estimated effect size for each SNP in the UK and NL populations. [file MEC-24-6148-s001.pdf]
