# Supplementary material for: Replicated analysis of the genetic architecture of quantitative traits in two wild great tit populations
Source: Mol Ecol. 2015 Dec 10;24(24):6148–62. doi: 10.1111/mec.13452 (PMC4738425; doi:10.1111/mec.13452)
Supplement: Supplementary file 2 — Appendix S1 Important fixed and random effects fitted for each trait. Appendix S2 Resolving the social pedigree. Appendix S3 Contributions of individual chromosomes to heritability of traits in the NL and UK. Appendix S4 Correspondence between the test statistics obtained from the QTL scans. Appendix S5 QTL mapping power analysis. Appendix S6 Suggestive QTL peaks. Appendix S7 Concordance of nominally significant QTL peaks between traits. Appendix S8 The concordance between QTL mapping and GWAS results for each trait within each population. Appendix S9 Concordance of estimated effect sizes for all SNPs from GWAS and multi‐SNP association analyses. [file MEC-24-6148-s002.docx]

**Supporting Information 1 - important fixed and random effects fitted for each trait**

Phenotypes were extracted for all genotyped individuals for each trait. Phenotypes were merged with a number of fixed and random effects measured in the populations, and the effects of these variables on the trait tested. For fixed effects, a linear model was constructed using the ‘lm’ function in R ([R Development Core Team 2012](#_ENREF_9)) and the significance of fixed terms tested using the ‘dropterm’ function to identify variables with a significant effect on the trait. The significance of each random effect was tested by constructing a linear mixed model with and without the random effect, using the ‘lmer’ function in the lme4 package ([Bates *et al.* 2011](#_ENREF_1)) in R. The log likelihood of the models was compared with a chi-squared statistic with one degree of freedom to assess the significance of each random effect. Note that due to differences in the information available from each population, random and fixed effects may be different between the two populations (Tables S1a and S1b).

Table S1a: Models fitted for each trait in the Netherlands (NL) and United Kingdom (UK) populations, with a description of each term in Table S1b.

| trait | fixed effects | random effects |
| --- | --- | --- |
| **NL:** | | |
| clutch size | overall mean + lay date | animal + permanent environment  + year + age |
| fledgling weight  (of offspring) | overall mean | animal + permanent environment + year + age + mother age + nest box + clutch size |
| adult weight | overall mean + sex + age | animal + permanent environment  + month + year |
| fledgling weight  (of individual) | overall mean + sex +  clutch size + age | animal + permanent environment  + day + month + year |
| tarsus length | overall mean + sex + age | animal + permanent environment  + month + year |
| wing length | overall mean + sex + age | animal + permanent environment  + month + year |
| exploratory behaviour * | overall mean | animal |
| **UK:** | | |
| clutch size | overall mean +  lay date + area | animal + permanent environment  + nest box + year + section + age |
| egg mass | overall mean + age +  lay date + area | animal + permanent environment  + nest box + year + section |
| fledgling weight  (of offspring) | overall mean + area + latitude + longitude + distance to edge + altitude | animal + permanent environment + year + nest box + lay date + clutch size + age |
| adult weight | overall mean + sex + age | animal + permanent environment  + month + section + year |
| fledgling weight  (of individual) | overall mean + sex + distance to edge + altitude + area + latitude + longitude | animal + day + month + year + nest box |
| tarsus length | overall mean + sex + age | animal + permanent environment  + month + section+ year |
| wing length | overall mean + sex + age | animal + permanent environment  + month + section+ year |
| exploratory behaviour | overall mean + age + sex  + assay number | animal + day |

*Note that the exploratory behaviour measurements were previously standardised for the effects of sex and age in the NL, and year in the UK.

Table S1b: An explanation of the individual and environmental variables contributing to trait variation. Continuous variables are indicated, others are fitted as categorical values.

| variable | description |
| --- | --- |
| age | age (in years) of individual |
| altitude | altitude (in metres) of nest box above sea level; continuous |
| animal | individual identity, fitted to estimate the additive genetic variance of the trait |
| area | area of the polygon (in hectares) of habitat surrounding the nest box; continuous |
| assay number | assay number |
| day | day of measurement |
| distance to edge | distance (in metres) to the edge of the woodland; continuous |
| latitude | latitude of nest box; continuous |
| lay date | date of first egg laid in nest; measured as days since March 31; continuous |
| longitude | longitude of nest box; continuous |
| month | month of measurement |
| mother age | age of mother (in years) |
| nest box | identity of nest box |
| permanent environment | effects on phenotype that are constant across  the individual's lifetime |
| section | location in Wytham Woods |
| sex | sex of individual |
| year | year of measurement |

**Supporting Information 2 - resolving the social pedigree**

The social pedigrees of the NL and UK genotyped individuals were determined from field observations. After excluding individuals with poor genotyping rates, genetic relationships in the pedigree were confirmed using a number of methods ([van Oers *et al.* 2014](#_ENREF_7); [Santure *et al.* 2013](#_ENREF_10)):

(i) Z-linked SNPs were used to confirm the sex of the genotyped individuals; homozygosity by locus (HL) of Z-linked markers was calculated using the 'GENHET' package ([Coulon 2010](#_ENREF_2)) in R version 2.11.1 ([R Development Core Team 2012](#_ENREF_9)). Individuals with HL values of 0-0.7 were assigned as male. Individuals with HL values of 0.9-1 were assigned as female to allow for the possibility of rare typing errors and the possibility of SNPs in pseudoautosomal regions that could therefore be heterozygous in ZW females. The distribution of HL values is bimodal, with no overlap between the sexes. Individuals whose genetic sex did not match their recorded sex were excluded from the datasets (8 NL and 9 UK individuals excluded).

(ii) genotyped individuals were checked for genetic compatibility with their genotyped parents and offspring by counting the number of Mendelian inheritance errors for autosomal SNPs. Inheritance errors were summed for each parent individually to determine whether mismatches were predominantly due to mismatches with the social father, likely caused by extra pair paternity (i.e. inconsistency between the social and genetic pedigree), in which case the pedigree link to the father was removed (29 NL and 109 UK individuals). In general, individuals who raised approximately equal numbers of mismatches with each parent were removed from analysis (25 NL and 82 UK individuals excluded).

(iii) identity by state (IBS) allele sharing at the autosomal markers was calculated between all pairs of individuals using PLINK ([Purcell *et al.* 2007](#_ENREF_8)). Individuals with identical genotypes (IBS values of >0.99) but different sample IDs were investigated and those that were incompatible with genotyped relatives were removed from the dataset (2 duplicate NL and 16 duplicate UK individuals removed). In instances where neither ID had genotyped relatives both individuals were removed from analysis (6 pairs of NL individuals and 14 pairs of UK individuals removed).

**Supporting Information 3 – contributions of individual chromosomes (chr) to heritability (*h*^2^) of traits in the NL and UK**. Numbers in parentheses indicate standard errors. The likelihood ratio test, LRT, tests whether the chromosome explains significant variation while the heritability explained can be used to test whether there is a linear relationship between chromosome size and contribution to overall additive genetic variance (see Figures 1a-1h). * indicates *p* < 0.05 (LRT of > 2.705544)

Table S3a: clutch size, egg mass and fledgling weight (of offspring)

|  |  |  |  |  | clutch size | | | | egg mass |  | fledgling weight (of offspring) | | | |
| --- | --- | --- | --- | --- | --- | --- | --- | --- | --- | --- | --- | --- | --- | --- |
|  |  |  |  |  | NL | | UK | | UK |  | NL | | UK | |
| chr | N markers | length (cM) | N genes | size (Mbp) | *h*^2^ | LRT | *h*^2^ | LRT | *h*^2^ | LRT | *h*^2^ | LRT | *h*^2^ | LRT |
| **1** | 579 | 139.9 | 1,254 | 119.6 | 0.152 (0.098) | 2.54 | 0.141 (0.073) | 4.51* | 0.118 (0.080) | 2.34 | 0.051 (0.076) | 0.46 | 0.131 (0.114) | 1.36 |
| **1A** | 415 | 93.6 | 972 | 73.7 | 0.065 (0.077) | 0.76 | 0 | 0 | 0.075 (0.063) | 1.83 | 0.044 (0.064) | 0.5 | 0 | 0 |
| **2** | 700 | 139.7 | 1,450 | 156.4 | 0.078 (0.093) | 0.78 | 0.078 (0.073) | 1.3 | 0.194 (0.085) | 6.39* | 0 | 0 | 0 | 0 |
| **3** | 596 | 114.9 | 1,290 | 112.6 | 0 | 0 | 0 | 0 | 0.025 (0.068) | 0.15 | 0.123 (0.081) | 2.44 | 0.092 (0.111) | 0.64 |
| **4** | 356 | 97.6 | 811 | 69.8 | 0 | 0 | 0 | 0 | 0.007 (0.052) | 0.02 | 0.087 (0.067) | 1.78 | 0 | 0 |
| **4A** | 103 | 59.4 | 39 | 20.7 | 0 | 0 | 0.019 (0.030) | 0.54 | 0.022 (0.035) | 0.47 | 0.027 (0.037) | 0.62 | 0 | 0 |
| **5** | 346 | 98.6 | 998 | 62.4 | 0 | 0 | 0.107 (0.062) | 3.14* | 0 | 0 | 0 | 0 | 0.040 (0.083) | 0.22 |
| **6** | 177 | 78 | 596 | 36.3 | 0.023 (0.053) | 0.2 | 0 | 0 | 0.027 (0.041) | 0.54 | 0.035 (0.044) | 0.78 | 0.081 (0.072) | 1.44 |
| **7** | 176 | 72.6 | 562 | 39.8 | 0 | 0 | 0.058 (0.043) | 2.51 | 0.010 (0.038) | 0.08 | 0 | 0 | 0 | 0 |
| **8** | 134 | 53.8 | 575 | 28 | 0.009 (0.045) | 0.04 | 0.030 (0.039) | 0.67 | 0.057 (0.044) | 2.2 | 0 | 0 | 0.039 (0.065) | 0.3 |
| **9** | 130 | 54.2 | 497 | 27.2 | 0.017 (0.043) | 0.2 | 0.024 (0.036) | 0.52 | 0 | 0 | 0 | 0 | 0 | 0 |
| **10** | 148 | 50.5 | 444 | 20.8 | 0.038 (0.051) | 0.6 | 0 | 0 | 0.006 (0.038) | 0.03 | 0 | 0 | 0.108 (0.070) | 3.18* |
| **11** | 135 | 58.2 | 397 | 21.4 | 0 | 0 | 0 | 0 | 0 | 0 | 0 | 0 | 0.055 (0.061) | 0.96 |
| **12** | 152 | 51.9 | 369 | 21.6 | 0.126 (0.065) | 4.3* | 0.109 (0.048) | 8.27* | 0 | 0 | 0.023 (0.042) | 0.34 | 0.005 (0.059) | 0.02 |
| **13** | 117 | 40.9 | 379 | 17 | 0.050 (0.048) | 1.32 | 0.028 (0.034) | 0.93 | 0 | 0 | 0.015 (0.036) | 0.18 | 0 | 0 |
| **14** | 126 | 49.2 | 426 | 16.4 | 0.022 (0.046) | 0.24 | 0 | 0 | 0.019 (0.037) | 0.3 | 0.029 (0.042) | 0.46 | 0.014 (0.055) | 0.08 |
| **15** | 173 | 49.1 | 381 | 14.4 | 0.059 (0.056) | 1.28 | 0 | 0 | 0.015 (0.042) | 0.14 | 0.084 (0.051) | 3.54* | 0.055 (0.070) | 0.62 |
| **17** | 96 | 45.4 | 336 | 11.6 | 0.051 (0.046) | 1.56 | 0 | 0 | 0 | 0 | 0 | 0 | 0 | 0 |
| **18** | 93 | 49.9 | 334 | 11.2 | 0.005 (0.038) | 0.02 | 0.004 (0.030) | 0.02 | 0 | 0 | 0.018 (0.036) | 0.22 | 0.065 (0.057) | 1.66 |
| **19** | 97 | 49.4 | 348 | 11.6 | 0 | 0 | 0.011 (0.033) | 0.1 | 0 | 0 | 0.050 (0.040) | 1.96 | 0.071 (0.061) | 1.56 |
| **20** | 155 | 49.4 | 356 | 15.7 | 0.053 (0.053) | 1.18 | 0.015 (0.039) | 0.15 | 0 | 0 | 0.005 (0.040) | 0.02 | 0 | 0 |
| **micros** | 308 | 411 | 1,808 | 40.2 | 0.041 (0.070) | 0.36 | 0.096 (0.061) | 2.8* | 0.051 (0.058) | 0.91 | 0.022 (0.059) | 0.14 | 0.127 (0.097) | 1.76 |

Table S3b: adult weight and fledgling weight (of individual)

|  |  |  |  |  | adult weight | | | | fledgling weight (of individual) | | | |
| --- | --- | --- | --- | --- | --- | --- | --- | --- | --- | --- | --- | --- |
|  |  |  |  |  | NL | | UK | | NL | | UK | |
| chr | N markers | length (cM) | N genes | size (Mbp) | *h*^2^ | LRT | *h*^2^ | LRT | *h*^2^ | LRT | *h*^2^ | LRT |
| **1** | 579 | 139.9 | 1,254 | 119.6 | 0 | 0 | 0.016 (0.028) | 0.32 | 0.725 (0.248) | 10.24* | 0.035 (0.060) | 0.35 |
| **1A** | 415 | 93.6 | 972 | 73.7 | 0 | 0 | 0.049 (0.028) | 3.74* | 0.304 (0.201) | 2.54 | 0.025 (0.050) | 0.26 |
| **2** | 700 | 139.7 | 1,450 | 156.4 | 0.405 (0.170) | 5.72* | 0.030 (0.032) | 0.96 | 0.076 (0.225) | 0.12 | 0 | 0 |
| **3** | 596 | 114.9 | 1,290 | 112.6 | 0 | 0 | 0.052 (0.030) | 3.7* | 0 | 0 | 0.019 (0.059) | 0.1 |
| **4** | 356 | 97.6 | 811 | 69.8 | 0 | 0 | 0.069 (0.028) | 8.38* | 0 | 0 | 0.015 (0.046) | 0.12 |
| **4A** | 103 | 59.4 | 39 | 20.7 | 0 | 0 | 0.002 (0.013) | 0.02 | 0 | 0 | 0 | 0 |
| **5** | 346 | 98.6 | 998 | 62.4 | 0.070 (0.107) | 0.52 | 0 | 0 | 0.071 (0.170) | 0.16 | 0.040 (0.046) | 0.87 |
| **6** | 177 | 78 | 596 | 36.3 | 0.016 (0.080) | 0.04 | 0.014 (0.019) | 0.58 | 0.251 (0.159) | 2.88* | 0.009 (0.032) | 0.09 |
| **7** | 176 | 72.6 | 562 | 39.8 | 0 | 0 | 0.049 (0.023) | 6.86* | 0.062 (0.135) | 0.2 | 0.014 (0.037) | 0.12 |
| **8** | 134 | 53.8 | 575 | 28 | 0 | 0 | 0 | 0 | 0 | 0 | 0.005 (0.029) | 0.04 |
| **9** | 130 | 54.2 | 497 | 27.2 | 0.186 (0.093) | 5.8* | 0 | 0 | 0.106 (0.123) | 0.86 | 0 | 0 |
| **10** | 148 | 50.5 | 444 | 20.8 | 0 | 0 | 0.002 (0.015) | 0.02 | 0.132 (0.144) | 0.8 | 0 | 0 |
| **11** | 135 | 58.2 | 397 | 21.4 | 0.168 (0.096) | 4.22* | 0.005 (0.015) | 0.14 | 0.015 (0.106) | 0.02 | 0.007 (0.031) | 0.05 |
| **12** | 152 | 51.9 | 369 | 21.6 | 0.111 (0.094) | 1.52 | 0.008 (0.016) | 0.32 | 0 | 0 | 0.012 (0.035) | 0.12 |
| **13** | 117 | 40.9 | 379 | 17 | 0 | 0 | 0.015 (0.017) | 0.98 | 0 | 0.02 | 0 | 0 |
| **14** | 126 | 49.2 | 426 | 16.4 | 0 | 0 | 0.005 (0.014) | 0.12 | 0 | 0 | 0.031 (0.036) | 0.82 |
| **15** | 173 | 49.1 | 381 | 14.4 | 0 | 0 | 0.030 (0.020) | 2.64 | 0 | 0 | 0 | 0 |
| **17** | 96 | 45.4 | 336 | 11.6 | 0.092 (0.076) | 1.94 | 0.007 (0.013) | 0.36 | 0 | 0 | 0 | 0 |
| **18** | 93 | 49.9 | 334 | 11.2 | 0 | 0 | 0 | 0 | 0 | 0 | 0 | 0 |
| **19** | 97 | 49.4 | 348 | 11.6 | 0 | 0 | 0.003 (0.013) | 0.06 | 0.097 (0.112) | 0.86 | 0.013 (0.029) | 0.23 |
| **20** | 155 | 49.4 | 356 | 15.7 | 0 | 0 | 0.010 (0.017) | 0.42 | 0 | 0 | 0 | 0 |
| **micros** | 308 | 411 | 1,808 | 40.2 | 0.102 (0.120) | 0.78 | 0.015 (0.024) | 0.46 | 0.031 (0.173) | 0.04 | 0.091 (0.054) | 3.53* |

Table S3c: tarsus length and wing length

|  |  |  |  |  | tarsus length | | | | wing length | | | |
| --- | --- | --- | --- | --- | --- | --- | --- | --- | --- | --- | --- | --- |
|  |  |  |  |  | NL | | UK | | NL | | UK | |
| chr | N markers | length (cM) | N genes | size (Mbp) | *h*^2^ | LRT | *h*^2^ | LRT | *h*^2^ | LRT | *h*^2^ | LRT |
| **1** | 579 | 139.9 | 1,254 | 119.6 | 0.198 (0.080) | 7.34* | 0.009 (0.034) | 0.06 | 0 | 0 | 0.013 (0.030) | 0.19 |
| **1A** | 415 | 93.6 | 972 | 73.7 | 0.005 (0.056) | 0.02 | 0 | 0 | 0.133 (0.059) | 9.44* | 0.072 (0.032) | 6.78* |
| **2** | 700 | 139.7 | 1,450 | 156.4 | 0.081 (0.078) | 1.14 | 0.001 (0.035) | 0 | 0.046 (0.074) | 0.4 | 0.040 (0.034) | 1.57 |
| **3** | 596 | 114.9 | 1,290 | 112.6 | 0.135 (0.077) | 3.46* | 0.062 (0.038) | 3.53* | 0 | 0 | 0.053 (0.034) | 2.75* |
| **4** | 356 | 97.6 | 811 | 69.8 | 0.107 (0.063) | 3.58* | 0.021 (0.029) | 0.6 | 0.081 (0.061) | 1.96 | 0.028 (0.025) | 1.62 |
| **4A** | 103 | 59.4 | 39 | 20.7 | 0 | 0 | 0.024 (0.021) | 1.84 | 0 | 0 | 0.008 (0.014) | 0.34 |
| **5** | 346 | 98.6 | 998 | 62.4 | 0.006 (0.054) | 0.02 | 0.072 (0.034) | 6.53* | 0.069 (0.058) | 1.54 | 0.051 (0.030) | 3.14* |
| **6** | 177 | 78 | 596 | 36.3 | 0.022 (0.041) | 0.32 | 0 | 0 | 0 | 0 | 0.035 (0.022) | 3.22* |
| **7** | 176 | 72.6 | 562 | 39.8 | 0.021 (0.043) | 0.24 | 0 | 0 | 0 | 0 | 0.050 (0.024) | 6.59* |
| **8** | 134 | 53.8 | 575 | 28 | 0.049 (0.043) | 1.4 | 0.015 (0.021) | 0.59 | 0 | 0 | 0.009 (0.016) | 0.34 |
| **9** | 130 | 54.2 | 497 | 27.2 | 0.013 (0.038) | 0.1 | 0.001 (0.018) | 0 | 0.042 (0.040) | 1.36 | 0 | 0 |
| **10** | 148 | 50.5 | 444 | 20.8 | 0.026 (0.042) | 0.36 | 0 | 0 | 0 | 0 | 0.010 (0.017) | 0.4 |
| **11** | 135 | 58.2 | 397 | 21.4 | 0 | 0 | 0 | 0 | 0.002 (0.033) | 0 | 0.044 (0.023) | 5.15* |
| **12** | 152 | 51.9 | 369 | 21.6 | 0.035 (0.042) | 0.78 | 0.017 (0.020) | 0.89 | 0.032 (0.039) | 0.8 | 0.002 (0.017) | 0.01 |
| **13** | 117 | 40.9 | 379 | 17 | 0 | 0 | 0 | 0 | 0.047 (0.039) | 1.68 | 0.028 (0.019) | 2.67 |
| **14** | 126 | 49.2 | 426 | 16.4 | 0 | 0 | 0.007 (0.019) | 0.14 | 0.014 (0.036) | 0.16 | 0.019 (0.018) | 1.36 |
| **15** | 173 | 49.1 | 381 | 14.4 | 0 | 0 | 0.057 (0.029) | 6.37* | 0.014 (0.040) | 0.12 | 0.009 (0.019) | 0.26 |
| **17** | 96 | 45.4 | 336 | 11.6 | 0.020 (0.034) | 0.38 | 0.019 (0.020) | 0.88 | 0 (0.03) | 0 | 0 | 0 |
| **18** | 93 | 49.9 | 334 | 11.2 | 0.049 (0.038) | 1.98 | 0 | 0 | 0.004 (0.029) | 0.02 | 0.009 (0.015) | 0.35 |
| **19** | 97 | 49.4 | 348 | 11.6 | 0 | 0 | 0.028 (0.020) | 3.45* | 0 | 0 | 0.004 (0.014) | 0.12 |
| **20** | 155 | 49.4 | 356 | 15.7 | 0.006 (0.038) | 0.02 | 0.001 (0.019) | 0.01 | 0.062 (0.044) | 2.46 | 0.009 (0.019) | 0.22 |
| **micros** | 308 | 411 | 1,808 | 40.2 | 0.004 (0.056) | 0.02 | 0 | 0 | 0 | 0 | 0.039 (0.027) | 2.46 |

Table S3d: exploratory behaviour

|  |  |  |  |  | exploratory behaviour | | | |
| --- | --- | --- | --- | --- | --- | --- | --- | --- |
|  |  |  |  |  | NL | | UK | |
| chr | N markers | length (cM) | N genes | size (Mbp) | *h*^2^ | LRT | *h*^2^ | LRT |
| **1** | 579 | 139.9 | 1,254 | 119.6 | 0.068 (0.103) | 0.52 | 0 | 0 |
| **1A** | 415 | 93.6 | 972 | 73.7 | 0 | 0 | 0.014 (0.059) | 0.05 |
| **2** | 700 | 139.7 | 1,450 | 156.4 | 0.143 (0.116) | 1.78 | 0.051 (0.075) | 0.52 |
| **3** | 596 | 114.9 | 1,290 | 112.6 | 0 | 0 | 0 | 0 |
| **4** | 356 | 97.6 | 811 | 69.8 | 0 | 0 | 0.082 (0.062) | 2.12 |
| **4A** | 103 | 59.4 | 39 | 20.7 | 0.058 (0.060) | 1.04 | 0.029 (0.038) | 0.67 |
| **5** | 346 | 98.6 | 998 | 62.4 | 0 | 0 | 0.065 (0.060) | 1.41 |
| **6** | 177 | 78 | 596 | 36.3 | 0.006 (0.058) | 0 | 0.014 (0.042) | 0.11 |
| **7** | 176 | 72.6 | 562 | 39.8 | 0.088 (0.071) | 1.86 | 0 | 0 |
| **8** | 134 | 53.8 | 575 | 28 | 0.047 (0.062) | 0.62 | 0.068 (0.043) | 4.31* |
| **9** | 130 | 54.2 | 497 | 27.2 | 0 | 0 | 0 | 0 |
| **10** | 148 | 50.5 | 444 | 20.8 | 0 | 0 | 0.021 (0.044) | 0.21 |
| **11** | 135 | 58.2 | 397 | 21.4 | 0 | 0 | 0 | 0 |
| **12** | 152 | 51.9 | 369 | 21.6 | 0 | 0 | 0.032 (0.044) | 0.6 |
| **13** | 117 | 40.9 | 379 | 17 | 0 | 0 | 0.035 (0.040) | 0.94 |
| **14** | 126 | 49.2 | 426 | 16.4 | 0 | 0 | 0.028 (0.040) | 0.57 |
| **15** | 173 | 49.1 | 381 | 14.4 | 0.006 (0.060) | 0 | 0.051 (0.047) | 1.47 |
| **17** | 96 | 45.4 | 336 | 11.6 | 0.057 (0.059) | 0.98 | 0 | 0 |
| **18** | 93 | 49.9 | 334 | 11.2 | 0.015 (0.049) | 0.1 | 0 | 0 |
| **19** | 97 | 49.4 | 348 | 11.6 | 0.006 (0.049) | 0.02 | 0 | 0 |
| **20** | 155 | 49.4 | 356 | 15.7 | 0.086 (0.067) | 2.22 | 0.012 (0.043) | 0.07 |
| **micros** | 308 | 411 | 1,808 | 40.2 | 0.114 (0.095) | 1.54 | 0.017 (0.058) | 0.09 |

**Supporting Information 4 - correspondence between the test statistics obtained from the QTL scans**

The permutation approach of Keightley & Knott (1999) was used to determine whether the LOD score for a QTL at a position in the genome were correlated (i) across traits within populations and (ii) within traits between populations. Because LOD scores at adjacent genome positions are correlated, the appropriate null distribution for the correlation between the two traits can be generated by permuting the data while maintaining the autocorrelation between adjacent positions ([Keightley & Knott 1999](#_ENREF_5)). The permutation approach illustrates that the observed correlations between cross-population trait LOD scores all lie within the sampling distribution for the correlation (Table S4a; Supporting Information Figures S3a-S3g). Thus, there is no evidence to suggest that there was any correspondence between populations for the test statistics obtained from the QTL scans.

Table S4a: correlations between LOD scores across the genome, with *p* values, between populations for each trait

| trait | correlation | *p* value |
| --- | --- | --- |
| clutch size | -0.045 | 0.850 |
| fledgling weight (of offspring) | -0.083 | 0.830 |
| adult weight | -0.003 | 0.173 |
| fledgling weight (of individual) | 0.094 | 0.127 |
| tarsus length | -0.073 | 0.791 |
| wing length | 0.175 | 0.055 |
| exploratory behaviour | -0.040 | 0.589 |

There was also no evidence that correlations between traits within populations were significant (Tables S4b and S4c).

**Supporting Information 5 - QTL mapping power analysis**

Simulations were used to assess the power to map QTL in the NL and UK great tit populations using the genotype/phenotype datasets presented in the manuscript. These analyses followed the procedure outlined in Slate ([2013](#_ENREF_11)) and Santure *et al*. ([2013](#_ENREF_10)) with minor modifications. Because such simulations are computationally demanding, analysis was limited to a 100 cM segment of chromosome 1, and SNPs were removed to obtain a density of ~1 SNP per cM. Simulating a higher SNP density would have increased computational time considerably, made little (if any) difference to power, and led to difficulties when estimating IBD matrices.

**Methods**

Simulating SNP and QTL genotypes

Genotypes were simulated for a single representative chromosome in the NL and UK QTL mapping pedigrees using the genomesim command from the R package Pedantics ([Morrissey & Wilson 2010](#_ENREF_6)). 200 and 100 replicate chromosomes were simulated for the NL and UK respectively; fewer replicates were simulated for the UK compared to the NL because the larger pedigree in the UK meant that QTL scans took considerably longer. UK allele frequencies and positions of 100 SNPs distributed along 100 cM of chromosome 1 were used. Genotypes were simulated for all individuals present in the pedigrees, but information was only retained for the animals genotyped in the empirical datasets. For each simulated chromosome, a QTL with additive allelic substitutions was also simulated at a random location (minor allele frequency = 0.4).

Estimating IBD matrices

For each of the simulated chromosomes, IBD coefficients between all pairs of individuals were derived at every cM using the software LOKI v2.4.5 ([Heath 1997](#_ENREF_3); [Heath *et al.* 1997](#_ENREF_4)), with 10,000 iterations for each position.

Simulating phenotypes

Phenotypes were simulated using the phensim command of Pedantics. Heritability (QTL + genome-wide polygenic effects) was set to values reflect empirical estimates in the UK population (clutch size = 0.40, egg mass = 0.4, fledgling weight (of offspring) = 0.60, adult weight = 0.40, fledgling weight (of individual) = 0.40, tarsus length = 0.60, wing length = 0.60, exploratory behaviour = 0.20). For each trait in each population, five separate phenotypic datasets were generated with a QTL explaining 0%, 5%, 10%, 20% and 40% of the phenotypic variance. Phenotypes were generated for all individuals present in the pedigree but only retained for the exact individuals for whom we had phenotypic information in the empirical datasets.

QTL mapping

QTL mapping was performed as described in the main article. To mimic the analysis performed in the main study, the presence of QTL every 5cM was tested from positions 0 cM to 100cM. Because these analyses are computationally intensive, analyses were limited to positions up to 55 cM on either side of a simulated QTL.

**Results**

Table S5a: Summary statistics for detection of simulated QTL in the NL great tit population. Proportion detected is the proportion of simulated chromosomes for which the largest LOD score exceeded the nominal, suggestive or significant threshold (up to 54.99 cM on either side of the true QTL position). Simulated and estimated QTL effect sizes are expressed as the proportion of trait variation explained by the QTL. Root mean square error (RMSE) across replicates are also presented. Note that, as expected, the mean effect size of detected QTL was highly inflated ([Slate 2013](#_ENREF_11)).

| trait | Simulated QTL effect | Proportion nominally significant | Mean effect size | RMSE | Proportion significant at ‘suggestive’ threshold | Mean effect size | RMSE | Proportion significant at ‘genomewide’ threshold’ | Mean effect size | RMSE |
| --- | --- | --- | --- | --- | --- | --- | --- | --- | --- | --- |
| clutch size | 0 | 0.24 | 0.42 (0.08) | 0.43 | 0 | 0.49 (NA) | 0.49 | 0 | NA (NA) | NA |
|  | 5 | 0.26 | 0.45 (0.09) | 0.41 | 0.02 | 0.55 (0.09) | 0.51 | 0 | NA (NA) | NA |
|  | 10 | 0.28 | 0.44 (0.10) | 0.35 | 0.04 | 0.57 (0.11) | 0.48 | 0 | NA (NA) | NA |
|  | 20 | 0.50 | 0.44 (0.10) | 0.27 | 0.06 | 0.59 (0.13) | 0.41 | 0.01 | 0.9 (NA) | 0.70 |
|  | 40 | 0.63 | 0.44 (0.09) | 0.10 | 0.10 | 0.54 (0.07) | 0.15 | 0 | NA (NA) | NA |
| fledgling weight (of offspring) | 0 | 0.28 | 0.57 (0.11) | 0.58 | 0.02 | 0.67 (0.09) | 0.68 | 0 | NA (NA) | NA |
|  | 5 | 0.22 | 0.55 (0.13) | 0.51 | 0 | 0.71 (NA) | 0.66 | 0 | NA (NA) | NA |
|  | 10 | 0.30 | 0.56 (0.13) | 0.48 | 0.04 | 0.68 (0.03) | 0.58 | 0 | NA (NA) | NA |
|  | 20 | 0.38 | 0.59 (0.12) | 0.40 | 0.08 | 0.71 (0.01) | 0.52 | 0 | NA (NA) | NA |
|  | 40 | 0.65 | 0.60 (0.11) | 0.23 | 0.18 | 0.71 (0.01) | 0.32 | 0.03 | 0.84 (0.06) | 0.44 |
| adult weight | 0 | 0.27 | 0.37 (0.09) | 0.38 | 0.02 | 0.60 (0.11) | 0.61 | 0 | NA (NA) | NA |
|  | 5 | 0.31 | 0.36 (0.08) | 0.32 | 0.02 | 0.47 (0.04) | 0.42 | 0 | NA (NA) | NA |
|  | 10 | 0.38 | 0.36 (0.09) | 0.28 | 0.04 | 0.54 (0.06) | 0.44 | 0 | NA (NA) | NA |
|  | 20 | 0.48 | 0.39 (0.08) | 0.21 | 0.06 | 0.49 (0.08) | 0.3 | 0 | NA (NA) | NA |
|  | 40 | 0.78 | 0.43 (0.09) | 0.09 | 0.32 | 0.5 (0.07) | 0.12 | 0.04 | 0.59 (0.06) | 0.20 |
| fledgling weight (of individual) | 0 | 0.18 | 0.40 (0.07) | 0.4 | 0 | NA (NA) | NA | 0 | NA (NA) | NA |
|  | 5 | 0.31 | 0.41 (0.09) | 0.37 | 0.02 | 0.54 (0.1) | 0.49 | 0 | NA (NA) | NA |
|  | 10 | 0.30 | 0.44 (0.09) | 0.36 | 0.04 | 0.56 (0.06) | 0.46 | 0 | NA (NA) | NA |
|  | 20 | 0.41 | 0.42 (0.09) | 0.24 | 0.06 | 0.51 (0.05) | 0.32 | 0 | NA (NA) | NA |
|  | 40 | 0.66 | 0.45 (0.10) | 0.11 | 0.16 | 0.55 (0.08) | 0.17 | 0.02 | 0.66 (0.08) | 0.27 |
| tarsus length | 0 | 0.26 | 0.32 (0.09) | 0.33 | 0.06 | 0.43 (0.05) | 0.43 | 0 | NA (NA) | NA |
|  | 5 | 0.30 | 0.33 (0.08) | 0.29 | 0.02 | 0.41 (0.07) | 0.37 | 0 | NA (NA) | NA |
|  | 10 | 0.47 | 0.34 (0.08) | 0.25 | 0.06 | 0.48 (0.09) | 0.39 | 0.01 | 0.49 (NA) | 0.39 |
|  | 20 | 0.58 | 0.36 (0.09) | 0.18 | 0.14 | 0.47 (0.07) | 0.27 | 0.02 | 0.54 (0.02) | 0.34 |
|  | 40 | 0.92 | 0.44 (0.10) | 0.11 | 0.54 | 0.50 (0.07) | 0.12 | 0.18 | 0.55 (0.06) | 0.16 |
| wing length | 0 | 0.24 | 0.37 (0.09) | 0.38 | 0.02 | 0.53 (0.08) | 0.53 | 0.01 | 0.63 (NA) | 0.63 |
|  | 5 | 0.30 | 0.37 (0.11) | 0.33 | 0.06 | 0.52 (0.08) | 0.48 | 0 | NA (NA) | NA |
|  | 10 | 0.35 | 0.40 (0.09) | 0.32 | 0.08 | 0.51 (0.07) | 0.41 | 0.01 | 0.52 (NA) | 0.42 |
|  | 20 | 0.60 | 0.42 (0.11) | 0.24 | 0.19 | 0.53 (0.07) | 0.34 | 0.02 | 0.65 (0.02) | 0.45 |
|  | 40 | 0.89 | 0.47 (0.11) | 0.13 | 0.48 | 0.55 (0.08) | 0.17 | 0.16 | 0.61 (0.05) | 0.22 |
| exploratory behaviour | 0 | 0.08 | 0.34 (0.07) | 0.35 | 0 | NA (NA) | NA | 0 | NA (NA) | NA |
|  | 5 | 0.15 | 0.34 (0.08) | 0.30 | 0.01 | 0.37 (NA) | 0.32 | 0 | NA (NA) | NA |
|  | 10 | 0.20 | 0.36 (0.08) | 0.27 | 0.01 | 0.56 (NA) | 0.46 | 0.01 | 0.56 (NA) | 0.46 |
|  | 20 | 0.30 | 0.35 (0.09) | 0.17 | 0.02 | 0.45 (0.03) | 0.25 | 0 | NA (NA) | NA |

Table S5b: Summary statistics for detection of simulated QTL in the UK great tit population. Proportion detected is the proportion of simulated chromosomes for which the largest LOD score exceeded the nominal, suggestive or significant threshold (up to 54.99 cM on either side of the true QTL position). Simulated and estimated QTL effect sizes are expressed as the proportion of trait variation explained by the QTL. Root mean square error (RMSE) across replicates are also presented.

| trait | Simulated QTL effect | Proportion nominally significant | Mean effect size | RMSE | Proportion significant at ‘suggestive’ threshold | Mean effect size | RMSE | Proportion significant at ‘genomewide’ threshold’ | Mean effect size | RMSE |
| --- | --- | --- | --- | --- | --- | --- | --- | --- | --- | --- |
| clutch size | 0 | 0.25 | 0.28 (0.06) | 0.29 | 0.02 | 0.37 (0.13) | 0.38 | 0 | NA (NA) | NA |
|  | 5 | 0.36 | 0.29 (0.06) | 0.24 | 0.02 | 0.32 (0.07) | 0.28 | 0 | NA (NA) | NA |
|  | 10 | 0.46 | 0.29 (0.06) | 0.2 | 0.09 | 0.36 (0.08) | 0.27 | 0 | NA (NA) | NA |
|  | 20 | 0.65 | 0.32 (0.07) | 0.14 | 0.15 | 0.40 (0.06) | 0.21 | 0.01 | 0.49 (NA) | 0.29 |
|  | 40 | 0.93 | 0.39 (0.08) | 0.08 | 0.65 | 0.43 (0.06) | 0.07 | 0.22 | 0.46 (0.05) | 0.08 |
| fledgling weight (of offspring) | 0 | 0.29 | 0.55 (0.14) | 0.57 | 0.02 | 0.83 (0.12) | 0.83 | 0 | NA (NA) | NA |
|  | 5 | 0.33 | 0.55 (0.12) | 0.52 | 0.03 | 0.64 (0.02) | 0.59 | 0 | NA (NA) | NA |
|  | 10 | 0.41 | 0.56 (0.14) | 0.47 | 0.04 | 0.71 (0.20) | 0.63 | 0.01 | 0.99 (NA) | 0.89 |
|  | 20 | 0.38 | 0.57 (0.14) | 0.4 | 0.03 | 0.81 (0.09) | 0.61 | 0 | NA (NA) | NA |
|  | 40 | 0.65 | 0.61 (0.12) | 0.24 | 0.21 | 0.71 (0.07) | 0.32 | 0.01 | 0.78 (NA) | 0.38 |
| adult weight | 0 | 0.27 | 0.13 (0.03) | 0.14 | 0.01 | 0.18 (NA) | 0.18 | 0 | NA (NA) | NA |
|  | 5 | 0.48 | 0.14 (0.03) | 0.1 | 0.09 | 0.19 (0.01) | 0.14 | 0 | NA (NA) | NA |
|  | 10 | 0.76 | 0.16 (0.04) | 0.07 | 0.25 | 0.20 (0.03) | 0.11 | 0.02 | 0.25 (0.03) | 0.15 |
|  | 20 | 0.96 | 0.22 (0.06) | 0.06 | 0.71 | 0.24 (0.05) | 0.07 | 0.31 | 0.29 (0.03) | 0.09 |
|  | 40 | 1 | 0.38 (0.05) | 0.05 | 1 | 0.38 (0.05) | 0.05 | 0.97 | 0.38 (0.04) | 0.04 |
| fledgling weight (of individual) | 0 | 0.3 | 0.13 (0.04) | 0.14 | 0.02 | 0.23 (0.08) | 0.24 | 0.01 | 0.29 (NA) | 0.29 |
|  | 5 | 0.42 | 0.15 (0.03) | 0.1 | 0.11 | 0.19 (0.02) | 0.14 | 0 | NA (NA) | NA |
|  | 10 | 0.76 | 0.16 (0.04) | 0.07 | 0.31 | 0.20 (0.03) | 0.1 | 0.05 | 0.24 (0.03) | 0.14 |
|  | 20 | 0.97 | 0.21 (0.05) | 0.06 | 0.73 | 0.23 (0.04) | 0.05 | 0.33 | 0.27 (0.03) | 0.08 |
|  | 40 | 1 | 0.39 (0.06) | 0.06 | 1 | 0.39 (0.06) | 0.06 | 0.98 | 0.39 (0.05) | 0.05 |
| tarsus length | 0 | 0.21 | 0.24 (0.04) | 0.24 | 0.02 | 0.33 (0.03) | 0.33 | 0 | NA (NA) | NA |
|  | 5 | 0.35 | 0.26 (0.06) | 0.21 | 0.04 | 0.37 (0.08) | 0.32 | 0 | NA (NA) | NA |
|  | 10 | 0.43 | 0.26 (0.05) | 0.17 | 0.08 | 0.34 (0.03) | 0.24 | 0 | NA (NA) | NA |
|  | 20 | 0.71 | 0.31 (0.08) | 0.13 | 0.37 | 0.36 (0.07) | 0.18 | 0.04 | 0.47 (0.04) | 0.27 |
|  | 40 | 0.96 | 0.38 (0.08) | 0.08 | 0.8 | 0.41 (0.06) | 0.06 | 0.39 | 0.44 (0.06) | 0.07 |
| wing length | 0 | 0.32 | 0.13 (0.03) | 0.13 | 0.04 | 0.19 (0.01) | 0.19 | 0 | NA (NA) | NA |
|  | 5 | 0.47 | 0.14 (0.04) | 0.09 | 0.1 | 0.19 (0.04) | 0.14 | 0.02 | 0.24 (0.05) | 0.19 |
|  | 10 | 0.73 | 0.15 (0.04) | 0.06 | 0.24 | 0.19 (0.04) | 0.1 | 0.03 | 0.27 (0.03) | 0.17 |
|  | 20 | 1 | 0.21 (0.05) | 0.05 | 0.84 | 0.23 (0.04) | 0.05 | 0.39 | 0.26 (0.03) | 0.07 |
|  | 40 | 1 | 0.39 (0.06) | 0.06 | 1 | 0.39 (0.06) | 0.06 | 1 | 0.39 (0.06) | 0.06 |
| exploratory behaviour | 0 | 0.2 | 0.21 (0.05) | 0.22 | 0.02 | 0.28 (0.02) | 0.28 | 0 | NA (NA) | NA |
|  | 5 | 0.35 | 0.22 (0.05) | 0.18 | 0.06 | 0.26 (0.07) | 0.22 | 0 | NA (NA) | NA |
|  | 10 | 0.45 | 0.21 (0.04) | 0.12 | 0.04 | 0.22 (0.03) | 0.12 | 0 | NA (NA) | NA |
|  | 20 | 0.71 | 0.23 (0.06) | 0.06 | 0.26 | 0.28 (0.05) | 0.09 | 0.03 | 0.34 (0.04) | 0.14 |

**Supporting Information 6 – suggestive QTL peaks**

Table S6a: all suggestive (LOD score > 1.620) peaks from the QTL mapping genome scans, with their corresponding LOD score. Peaks exceeding a LOD score of 3.062 are classified as genome wide significant and are indicated with an asterisk (*). For the maximum of each QTL peak, *q*^2^ indicates the proportion of phenotypic variance explained by the QTL, while *h*^2^ is the residual heritability after having fitted the QTL effect. Note that QTL effect sizes are likely to be substantially overestimated ([Slate 2013](#_ENREF_11)).

| trait | chromosome | position (cM) | LOD score | *q*^2^ | *h*^2^ |
| --- | --- | --- | --- | --- | --- |
| UK clutch size | 20 | 5 | 2.002 | 0.307 | 0.094 |
| NL adult weight | 1A | 25 | 1.772 | 0.371 | 0.094 |
| NL adult weight | 2 | 0 | 2.562 | 0.445 | 0.000 |
| NL adult weight | 2 | 5 | 2.519 |  |  |
| NL adult weight | 2 | 10 | 1.898 |  |  |
| NL adult weight | 2 | 15 | 2.823 | 0.424 | 0.027 |
| NL adult weight | 2 | 20 | 2.319 |  |  |
| NL adult weight | 2 | 25 | 2.350 |  |  |
| NL adult weight | 2 | 30 | 2.276 |  |  |
| NL adult weight | 2 | 105 | 1.707 | 0.348 | 0.116 |
| NL adult weight | 2 | 110 | 1.694 |  |  |
| NL adult weight | 4 | 0 | 1.833 |  |  |
| NL adult weight | 4 | 5 | 2.601 | 0.427 | 0.033 |
| NL adult weight | 4 | 10 | 2.345 |  |  |
| NL adult weight | 4 | 15 | 2.471 |  |  |
| NL adult weight | 6 | 35 | 1.755 | 0.464 | 0.000 |
| NL adult weight | 7 | 60 | 1.959 | 0.419 | 0.043 |
| NL adult weight | 7 | 65 | 1.837 |  |  |
| NL adult weight | 7 | 70 | 2.276 | 0.455 | 0.012 |
| NL adult weight | 8 | 45 | 1.672 |  |  |
| NL adult weight | 8 | 50 | 1.993 | 0.357 | 0.093 |
| NL adult weight | 8 | 55 | 1.876 |  |  |
| NL adult weight | 11 | 30 | 1.876 |  |  |
| NL adult weight | 11 | 35 | 3.101* | 0.440 | 0.018 |
| NL adult weight | 11 | 40 | 2.063 |  |  |
| NL adult weight | 14 | 20 | 1.993 |  |  |
| NL adult weight | 14 | 25 | 2.363 | 0.439 | 0.023 |
| NL adult weight | 14 | 30 | 2.293 |  |  |
| NL adult weight | 15 | 15 | 2.219 |  |  |
| NL adult weight | 15 | 20 | 2.454 |  |  |
| NL adult weight | 15 | 25 | 2.927 |  |  |
| NL adult weight | 15 | 30 | 3.170* |  |  |
| NL adult weight | 15 | 35 | 3.209* | 0.459 | 0.000 |
| NL adult weight | 15 | 40 | 2.402 |  |  |
| NL adult weight | 15 | 45 | 2.445 |  |  |
| NL adult weight | 15 | 50 | 2.202 |  |  |
| NL adult weight | 19 | 40 | 1.902 | 0.380 | 0.081 |
| NL adult weight | 24 | 15 | 1.889 |  |  |
| NL adult weight | 24 | 20 | 2.610 |  |  |
| NL adult weight | 24 | 25 | 3.014 | 0.462 | 0.000 |
| NL adult weight | 24 | 30 | 1.833 |  |  |
| NL adult weight | 24 | 35 | 2.232 | 0.445 | 0.008 |
| NL adult weight | 27 | 50 | 1.689 | 0.435 | 0.027 |
| NL adult weight | 28 | 10 | 1.946 |  |  |
| NL adult weight | 28 | 15 | 2.254 |  |  |
| NL adult weight | 28 | 20 | 2.002 |  |  |
| NL adult weight | 28 | 25 | 2.680 |  |  |
| NL adult weight | 28 | 30 | 3.131* |  |  |
| NL adult weight | 28 | 35 | 3.500* |  |  |
| NL adult weight | 28 | 40 | 3.865* |  |  |
| NL adult weight | 28 | 45 | 4.074* | 0.466 | 0.000 |
| NL adult weight | 28 | 50 | 2.662 |  |  |
| UK adult weight | 1A | 30 | 2.515 | 0.208 | 0.189 |
| UK adult weight | 8 | 45 | 2.736 | 0.200 | 0.186 |
| UK adult weight | 8 | 50 | 2.627 |  |  |
| UK adult weight | 15 | 20 | 1.781 | 0.164 | 0.239 |
| UK adult weight | 18 | 15 | 1.894 | 0.155 | 0.245 |
| UK fledgling weight (of individual) | 1 | 65 | 1.728 | 0.215 | 0.390 |
| UK tarsus length | 3 | 50 | 1.689 | 0.412 | 0.207 |
| UK tarsus length | 3 | 70 | 2.116 | 0.428 | 0.198 |
| UK tarsus length | 3 | 75 | 1.777 |  |  |
| UK tarsus length | 3 | 80 | 1.757 |  |  |
| UK tarsus length | 3 | 105 | 2.135 | 0.425 | 0.199 |
| UK tarsus length | 6 | 35 | 2.334 |  |  |
| UK tarsus length | 6 | 40 | 2.426 | 0.430 | 0.193 |
| UK tarsus length | 13 | 5 | 1.646 | 0.557 | 0.089 |
| UK tarsus length | 14 | 35 | 2.980 | 0.525 | 0.131 |
| UK tarsus length | 14 | 40 | 2.266 |  |  |
| UK tarsus length | 15 | 25 | 2.850 | 0.627 | 0.000 |
| UK tarsus length | 17 | 30 | 1.771 | 0.380 | 0.286 |
| UK wing length | 4A | 50 | 1.926 |  |  |
| UK wing length | 4A | 55 | 2.020 | 0.203 | 0.362 |
| UK wing length | 4A | 60 | 1.652 |  |  |
| UK wing length | 6 | 70 | 2.168 | 0.176 | 0.394 |

**Supporting Information 7 – concordance of nominally significant QTL peaks between traits**

Table S7a: all QTL peaks that are nominally significant in the same trait in both the NL and UK populations (LOD score > 0.588). A value of “y” in a column indicates that the position is nominally significant in both populations.

| chromosome | position (cM) | adult weight | fledgling weight | tarsus length | wing length |
| --- | --- | --- | --- | --- | --- |
|  |  |  | (of individual) |  |  |
| 1 | 40 | y |  |  |  |
| 1 | 45 | y |  |  |  |
| 1 | 60 |  | y |  |  |
| 1 | 65 |  | y |  |  |
| 1 | 70 | y |  |  |  |
| 1A | 30 | y |  |  |  |
| 2 | 50 |  |  | y |  |
| 2 | 55 |  |  | y |  |
| 4 | 15 | y |  |  |  |
| 4 | 20 | y |  |  |  |
| 4A | 50 |  |  |  | y |
| 8 | 10 |  |  |  | y |
| 8 | 15 |  |  |  | y |
| 8 | 20 |  |  |  | y |
| 8 | 25 |  |  |  | y |
| 8 | 30 | y |  |  | y |
| 8 | 40 | y |  |  |  |
| 8 | 45 | y |  |  |  |
| 8 | 50 | y |  |  |  |
| 8 | 55 | y |  |  |  |
| 15 | 15 | y |  |  |  |
| 15 | 20 | y |  |  |  |
| 15 | 25 | y |  |  |  |
| 18 | 0 | y |  |  |  |
| 19 | 5 | y |  |  |  |
| 21 | 40 |  | y |  |  |
| 21 | 45 |  | y |  |  |
| 21 | 50 |  | y |  |  |
| 25A | 0 | y |  |  |  |
| 25A | 5 | y |  |  |  |
| 28 | 15 | y |  |  |  |

**Supporting Information 8:** The concordance between QTL mapping and GWAS results for each trait within each population was tested by comparing the observed and expected counts of SNPs being nominally significant in both, one or neither of the GWAS and QTL analysis with a χ^2^ test. There was no concordance evident in any trait. Traits not tested were those where no QTL positions reached nominal significance.

|  | NL *p* value | UK *p* value |
| --- | --- | --- |
| clutch size | not tested | 0.558 |
| egg mass | **-** | 0.916 |
| fledgling weight (of offspring) | 0.347 | not tested |
| adult weight | 0.492 | 0.965 |
| fledgling weight (of individual) | 0.873 | 0.728 |
| tarsus length | 0.984 | 0.646 |
| wing length | 0.993 | 0.760 |
| exploratory behaviour | 0.901 | 0.915 |

**Supporting Information 9 - concordance of estimated effect sizes for all SNPs from GWAS and multi-SNP association analyses** (Table S9a). There was good agreement in the SNP estimated effect sizes from the GWAS and from the multi-SNP analysis, suggesting that the multi-SNP analysis was not biased by population structure

Table S9a: concordance of estimated SNP effect sizes. Numbers are correlations between the estimated effect sizes from the GWAS analysis and the first, second and third runs of the multi-SNP analysis.

| trait | NL | UK |
| --- | --- | --- |
| clutch size | 0.807, 0.770, 0.756 | 0.520, 0.542, 0.515 |
| egg mass | - | 0.449, 0.493, 0.484 |
| fledgling weight (of offspring) | 0.504, 0.491, 0.439 | 0.568, 0.541, 0.667 |
| adult weight | 0.516, 0.646, 0.630 | 0.640, 0.632, 0.651 |
| fledgling weight (of individual) | 0.671, 0.678, 0.653 | 0.607, 0.629, 0.593 |
| tarsus length | 0.690, 0.704, 0.710 | 0.717, 0.706, 0.702 |
| wing length | 0.661, 0.787, 0.444* | 0.607, 0.611, 0.615 |
| exploratory behaviour | 0.239*, 0.457, 0.541 | 0.741, 0.784, 0.762 |

*poor convergence, results not used

**Supporting Information References**

Bates D, Maechler M, Bolker B (2011) lme4: Linear mixed-effects models using S4 classes. <http://CRAN.R-project.org/package=lme4>.

Coulon A (2010) GENHET: an easy-to-use R function to estimate individual heterozygosity. *Molecular Ecology Resources* **10**, 167–169.

Heath SC (1997) Markov chain Monte Carlo segregation and linkage analysis for oligogenic models. *American Journal of Human Genetics* **61**, 748-760.

Heath SC, Snow GL, Thompson EA, Tseng C, Wijsman EM (1997) MCMC segregation and linkage analysis. *Genetic Epidemiology* **14**, 1011-1015.

Keightley PD, Knott SA (1999) Testing the correspondence between map positions of quantitative trait loci. *Genetical Research* **74**, 323-328.

Morrissey MB, Wilson A (2010) pedantics: an r package for pedigree-based genetic simulation and pedigree manipulation, characterization and viewing. *Molecular Ecology Resources* **10**, 711–719.

van Oers K, Santure AW, De Cauwer I*, et al.* (2014) Replicated independent high-density genetic maps of two great tit populations reveal new insights into avian genome structure and evolution. *Heredity* **112**, 307-316.

Purcell S, Neale B, Todd-Brown K*, et al.* (2007) PLINK: a toolset for whole-genome association and population-based linkage analysis. *American Journal of Human Genetics* **81**, 559-575.

R Development Core Team (2012) *R: A Language and Environment for Statistical Computing* R Foundation for Statistical Computing, Vienna, Austria.

Santure AW, De Cauwer I, Robinson MR*, et al.* (2013) Genomic dissection of variation in clutch size and egg mass in a wild great tit (*Parus major*) population. *Molecular Ecology* **22**, 3949–3962.

Slate J (2013) From Beavis to beak colour: a simulation study to examine how much QTL mapping can reveal about the genetic architecture of quantitative traits within populations. *Evolution* **67**, 1251–1262.
